# Supplementary material for: Accelerated brain ageing during the COVID-19 pandemic
Source: Nat Commun. 2025 Jul 22;16:6411. doi: 10.1038/s41467-025-61033-4 (PMC12284169; doi:10.1038/s41467-025-61033-4)
Supplement: Supplementary file 1 — Suppkementary Information [file 41467_2025_61033_MOESM1_ESM.pdf]

Supplementary Materials

Accelerated Brain Ageing During the COVID-19 Pandemic

Ali-Reza Mohammadi-Nejad[1,2,3], Martin Craig[2], Eleanor F. Cox[1,4], Xin Chen[5], R. Gisli Jenkins[6,7,8], Susan Francis[1,4], Stamatios N. Sotiropoulos[1,2,3*#], Dorothee P. Auer[1,2,3*#]

[1] National Institute for Health Research (NIHR) Nottingham Biomedical Research Centre, Queen's Medical Centre, Nottingham, United Kingdom

[2] Sir Peter Mansfield Imaging Centre, School of Medicine, University of Nottingham, Nottingham, United Kingdom

[3] Mental Health and Clinical Neurosciences, School of Medicine, University of Nottingham, Nottingham, United Kingdom

[4] Sir Peter Mansfield Imaging Centre, School of Physics and Astronomy, University of Nottingham, Nottingham, United Kingdom

[5] School of Computer Science, University of Nottingham, Nottingham, United Kingdom

[6] National Heart and Lung Institute, Imperial College London, London, United Kingdom

[7] National Institute for Health Research (NIHR) Imperial Biomedical Research Centre, Imperial College London, London, United Kingdom

[8] Department of Interstitial Lung Disease, Royal Brompton and Harefield Hospital, Guys and St Thomas' NHS Foundation Trust, London, United Kingdom

[*] Corresponding authors

[#] *Equal Contribution*

Health Outcomes and Clinical Characterization

To mitigate potential bias in brain age caused by disease effects, we excluded subjects who had any type of chronic disorders (such as dementia, parkinsonism, multiple sclerosis (MS), schizophrenia, depression, bipolar disorder, or cancer)[1,2] before their scans. The full list of 17 chronic diseases is in Table S1. These conditions have been identified using main and secondary ICD-10 codes (Data-Fields 41202, 41204, 41270, and 41271) and self-reports (Data-Fields 20001 and 20002) available in the UK Biobank dataset. For each individual, the recorded date of diagnosis has been compared across self-report (Data-Fields 20006 and 20008) and healthcare (Data-Fields 41262, 41280, and 41281) sources for each disease category, to determine whether the illness onset/diagnosis preceded or occurred after the first brain imaging scan.

Dimensionality Reduction Using Singular Value Decomposition (SVD)

To improve model performance and reduce noise, we applied dimensionality reduction to the predictor variables using Singular Value Decomposition (SVD) within each cross-validation fold. During each iteration, SVD was first performed on the training data, retaining the top 50 components. This selection was based on the work of Smith et al.[3], who demonstrated that retaining 50 components provides an optimal trade-off between model accuracy and interpretability. In our study, these retained components captured approximately 58% of the total variance for grey matter (GM) models and 79% for white matter (WM) models, preserving critical patterns in the data while minimising noise and redundancy.

The dimensionality reduction was applied exclusively to the training data. The derived component space defined by the SVD matrices was then used to project the test data onto the same lower-dimensional space. This standardised projection ensured that model

parameters estimated from the training data were applicable to the test data, maintaining a consistent feature space across both datasets. Such an approach enabled us to evaluate model performance reliably while minimising potential test set bias.

Additionally, SVD reduction was embedded within a 20-fold cross-validation framework to prevent inflated model success estimates stemming from data dependencies. In each fold, the data was partitioned into 20 roughly equal-sized groups. The model parameters, including confound removal, SVD reduction, and model fitting stages, were estimated using 95% of the data, while the remaining 5% served as an independent test set. This cross-validation approach ensured a robust, unbiased evaluation of the model's performance on unseen data. Even when using the full (no SVD) model or retaining the maximum number of components, as noted by Smith et al.[3], our results were not substantially influenced by overfitting.

To further illustrate the stability of our dimensionality reduction approach, we observed consistent variance explained by the first 50 components across the 20-fold cross-validation:

- Female GM: Mean variance explained = 57.8% ± 0.014
- Male GM: Mean variance explained = 57.9% ± 0.028
- Female WM: Mean variance explained = 79.0% ± 0.019
- Male WM: Mean variance explained = 78.7% ± 0.010

These results demonstrate minimal variation across cross-validation folds, underscoring the robustness and stability of our dimensionality reduction approach. By focusing on the most informative components, our approach balances computational efficiency, predictive power, and interpretability, ensuring robust generalisability across unseen data.

Deprivation Indices

In this study, we examined how variations in the brain age gap relate to different measures of deprivation, including education, employment, health, housing, and income scores. These deprivation metrics were derived from studies conducted in British local councils, presented separately for England, Wales, and Scotland. Relevant data fields include: education scores (Data-Fields: 26414, 26421, 26431), employment scores (Data-Fields: 26412, 26419, 26429), health scores (Data-Fields: 26413, 26420, 26430), housing scores (Data-Fields: 26415, 26423, 26432), and income scores (Data-Fields: 26411, 26418, 26428).

These deprivation indices capture various dimensions of socio-economic hardship across small geographic areas. Factors such as income insufficiency, unemployment, poor health, limited educational attainment, challenges in accessing adequate housing or essential services, exposure to crime, and a degraded living environment are each assessed through distinct indicators. These granular measurements enable the identification of regions facing the greatest socio-economic challenges and offer a framework for understanding the diverse hardships encountered by residents. By aggregating these measures, it is possible to create rankings that reflect varying levels of deprivation and highlight socio-economic disparities within the UK.

To categorise participants, we classified deprivation scores into 'high' and 'low' levels across Wales, Scotland, and England. Participants scoring above the 70th percentile for education, employment, health, housing, and income were assigned to the 'high' category, whereas those scoring below the 30th percentile were placed in the 'low' category. Any participants with missing information were excluded from the analysis. Importantly, these deprivation scores were derived solely from baseline data, as no longitudinal socio-economic measures are available in the UK Biobank dataset. Specifically,

the indices of multiple deprivation were collected in 2008, 2009, and 2010 for Wales, Scotland, and England, respectively, and subsequent follow-up measures for these indices do not exist.

Supplementary Table 1. The full list of 17 chronic diseases.

No.	Disease
1	Ischemic heart disease
2	Hypertensive diseases
3	Stroke
4	COPD
5	CKD
6	Dementia
7	Parkinsonism
8	Multiple Sclerosis
9	Diabetes
10	Cirrhosis
11	Osteoarthritis
12	Osteoporosis
13	Schizophrenia
14	Depression
15	Bipolar disorder
16	Cancer
17	Fibrosis

Supplementary Table 2. Main participant characteristics in different groups. P-values reflect paired t-tests across groups (uncorrected).

	**Pandemic (G1)**	**No Pandemic (G2)**	**Pandemic – subgroup COVID-19 (G3)**	**Pandemic – subgroup No COVID-19 (G4)**	**P-uncorr:** G1 vs G2	**P-uncorr:** G2 vs G3 vs G4
Number of participants	432	564	134	298	---	---
Sex (F/M)	255/177	297/267	78/56	177/121	0.05	0.13
Age 1st scan	58.53 (6.68)	58.93 (5.80)	58.73 (7.00)	58.44 (6.54)	0.05	0.24
Age 2nd scan	62.18 (6.40)	61.19 (5.79)	62.49 (6.83)	62.04 (6.19)	0.07	0.2
Inter-scan intervals (months)	43.78 (17.53)	27.11 (1.42)	45.02 (17.71)	43.22 (17.42)	2E-15	9.60E-87
Ethnicity (White/Non-white)	419/13	549/15	134/0	287/11	0.63	0.08
Weight 1st scan (kg)	74.30 (13.82)	74.07 (14.27)	74.17 (13.58)	74.36 (13.93)	0.8	0.97
Weight 2nd scan (kg)	74.40 (14.28)	74.23 (14.12)	74.78 (14.13)	74.23 (14.34)	0.85	0.87
Waist/Hip ratio 1st scan	0.86 (0.08)	0.86 (0.08)	0.85 (0.08)	0.86 (0.08)	0.37	0.66
Waist/Hip ratio 2nd scan	0.88 (0.08)	0.87 (0.09)	0.88 (0.09)	0.88 (0.08)	0.05	0.15
BMI 1st scan (kg m^{-2})	25.74 (4.05)	25.46 (3.97)	25.86 (4.10)	25.69 (4.03)	0.27	0.54
BMI 2nd scan (kg m^{-2})	25.79 (4.27)	25.57 (3.96)	26.00 (4.40)	25.69 (4.21)	0.41	0.75
BP – systolic 1st scan (mmHg)	131.14 (17.21)	133.24 (18.62)	129.23 (19.80)	132.29 (15.36)	0.46	0.89
BP – systolic 2nd scan (mmHg)	126.86 (16.29)	127.46 (16.23)	123.74 (16.89)	128.71 (15.62)	0.81	0.61
BP – diastolic 1st scan (mmHg)	77.94 (9.36)	77.94 (8.92)	76.81 (11.20)	78.62 (7.99)	1	0.89
BP – diastolic 2nd scan (mmHg)	77.77 (9.15)	77.67 (9.15)	77.06 (10.32)	78.19 (8.34)	0.94	0.98
Alcohol intake freq. score 1st scan	3.05 (1.27)	3.15 (1.28)	2.90 (1.29)	3.11 (1.25)	0.07	0.05
Alcohol intake freq. score 2nd scan	3.05 (1.28)	3.18 (1.28)	2.95 (1.28)	3.09 (1.28)	0.14	0.13
Smoking status 1st scan	0.36 (0.55)	0.33 (0.56)	0.40 (0.60)	0.35 (0.52)	0.4	0.44
Smoking status 2nd scan	0.35 (0.53)	0.31 (0.53)	0.36 (0.54)	0.35 (0.52)	0.17	24
Age completed education	17.65 (2.48)	17.42 (2.26)	17.70 (2.93)	17.63 (2.20)	0.28	0.41
Index of multiple deprivation	15.53 (12.47)	14.39 (12.02)	15.31 (11.99)	15.63 (12.69)	0.15	0.34
Number in household 1st scan	3.24 (1.14)	3.16 (1.08)	3.10 (1.04)	3.31 (1.17)	0.26	0.07
Number in household 2nd scan	2.16 (1.14)	3.17 (1.10)	3.14 (1.07)	3.17 (1.17)	0.9	0.97
Daily moderate activity (min) 1st scan	56.11 (66.69)	58.91 (62.62)	67.82 (79.48)	50.71 (59.10)	0.52	0.08
Daily moderate activity (min) 2nd scan	54.42 (55.14)	54.53 (56.06)	68.24 (63.55)	51.65 (50.39)	0.98	0.53
General health (1 – 4) 1st scan	1.82 (0.60)	1.78 (0.58)	1.84 (0.60)	1.80 (0.60)	0.06	0.18
General health (1 – 4) 2nd scan	1.87 (0.62)	1.82 (0.65)	1.92 (0.65)	1.84 (0.61)	0.05	0.07
Hand grips mean (kg) 1st scan	31.34 (10.13)	32.05 (10.51)	31.18 (10.23)	31.41 (10.08)	0.1	0.27
Hand grips mean (kg) 2nd scan	28.89 (9.99)	30.09 (11.00)	28.82 (10.17)	28.92 (9.91)	0.12	0.06

BMI: Body mass index, BP: Blood pressure.

- For variables not normally distributed, we employed nonparametric tests.
- Age and time intervals between scans were compared using two-sample Kolmogorov-Smirnov tests (two-sided) between "Pandemic" and "No Pandemic" groups.
- Sex and ethnicity were compared using $\chi 2$ tests (two-sided) between "Pandemic" and "No Pandemic" groups, as well as between "Pandemic – COVID-19", "Pandemic – No COVID-19", and "No Pandemic" groups.
- Characteristics such as weight, waist/hip ratio, BMI, blood pressure (systolic and diastolic), alcohol intake frequency score, smoking status, age completed education, number in household, daily moderate activity, general health, and hand grips mean were compared between "Pandemic" and "No Pandemic" groups using two-sample t-tests (two-sided).
- Similar characteristics were also compared between "Pandemic – COVID-19", "Pandemic – No COVID-19", and "No Pandemic" groups using Kruskal-Wallis tests (two-sided).

Supplementary Table 3. List of grey matter IDPs used for brain age prediction models.

No.	UKBB Data Field	IDP name
1	25000	Volumetric scaling from the T1 head image to standard space
2	25001	Volume of peripheral cortical grey matter (from T1 brain image, normalised for head size)
3	25002	Volume of peripheral cortical grey matter (from T1 brain image)
4	25005	Volume of grey matter (from T1 brain image, normalised for head size)
5	25006	Volume of grey matter (from T1 brain image)
6	25009	Volume of brain (grey+white, from T1 brain image, normalised for head size)
7	25010	Volume of brain (grey+white, from T1 brain image)
8	25011	Volume of left thalamus (from T1 brain image)
9	25012	Volume of right thalamus (from T1 brain image)
10	25013	Volume of left caudate (from T1 brain image)
11	25014	Volume of right caudate (from T1 brain image)
12	25015	Volume of left putamen (from T1 brain image)
13	25016	Volume of right putamen (from T1 brain image)
14	25017	Volume of left pallidum (from T1 brain image)
15	25018	Volume of right pallidum (from T1 brain image)
16	25019	Volume of left hippocampus (from T1 brain image)
17	25020	Volume of right hippocampus (from T1 brain image)
18	25021	Volume of left amygdala (from T1 brain image)
19	25022	Volume of right amygdala (from T1 brain image)
20	25023	Volume of left accumbens (from T1 brain image)
21	25024	Volume of right accumbens (from T1 brain image)
22	25025	Volume of brain stem + 4th ventricle (from T1 brain image)
23	25782	volume of grey matter in Left Frontal Pole
24	25783	volume of grey matter in Right Frontal Pole
25	25784	volume of grey matter in Left Insular Cortex
26	25785	volume of grey matter in Right Insular Cortex
27	25786	volume of grey matter in Left Superior Frontal Gyrus
28	25787	volume of grey matter in Right Superior Frontal Gyrus
29	25788	volume of grey matter in Left Middle Frontal Gyrus
30	25789	volume of grey matter in Right Middle Frontal Gyrus
31	25790	volume of grey matter in Left Inferior Frontal Gyrus, pars triangularis
32	25791	volume of grey matter in Right Inferior Frontal Gyrus, pars triangularis
33	25792	volume of grey matter in Left Inferior Frontal Gyrus, pars opercularis
34	25793	volume of grey matter in Right Inferior Frontal Gyrus, pars opercularis
35	25794	volume of grey matter in Left Precentral Gyrus
36	25795	volume of grey matter in Right Precentral Gyrus
37	25796	volume of grey matter in Left Temporal Pole
38	25797	volume of grey matter in Right Temporal Pole
39	25798	volume of grey matter in Left Superior Temporal Gyrus, anterior division
40	25799	volume of grey matter in Right Superior Temporal Gyrus, anterior division
41	25800	volume of grey matter in Left Superior Temporal Gyrus, posterior division
42	25801	volume of grey matter in Right Superior Temporal Gyrus, posterior division
43	25802	volume of grey matter in Left Middle Temporal Gyrus, anterior division
44	25803	volume of grey matter in Right Middle Temporal Gyrus, anterior division
45	25804	volume of grey matter in Left Middle Temporal Gyrus, posterior division
46	25805	volume of grey matter in Right Middle Temporal Gyrus, posterior division
47	25806	volume of grey matter in Left Middle Temporal Gyrus, temporooccipital part
48	25807	volume of grey matter in Right Middle Temporal Gyrus, temporooccipital part
49	25808	volume of grey matter in Left Inferior Temporal Gyrus, anterior division
50	25809	volume of grey matter in Right Inferior Temporal Gyrus, anterior division
51	25810	volume of grey matter in Left Inferior Temporal Gyrus, posterior division
52	25811	volume of grey matter in Right Inferior Temporal Gyrus, posterior division
53	25812	volume of grey matter in Left Inferior Temporal Gyrus, temporooccipital part
54	25813	volume of grey matter in Right Inferior Temporal Gyrus, temporooccipital part
55	25814	volume of grey matter in Left Postcentral Gyrus
56	25815	volume of grey matter in Right Postcentral Gyrus
57	25816	volume of grey matter in Left Superior Parietal Lobule
58	25817	volume of grey matter in Right Superior Parietal Lobule
59	25818	volume of grey matter in Left Supramarginal Gyrus, anterior division
60	25819	volume of grey matter in Right Supramarginal Gyrus, anterior division
61	25820	volume of grey matter in Left Supramarginal Gyrus, posterior division
62	25821	volume of grey matter in Right Supramarginal Gyrus, posterior division
63	25822	volume of grey matter in Left Angular Gyrus
64	25823	volume of grey matter in Right Angular Gyrus
65	25824	volume of grey matter in Left Lateral Occipital Cortex, superior division
66	25825	volume of grey matter in Right Lateral Occipital Cortex, superior division
67	25826	volume of grey matter in Left Lateral Occipital Cortex, inferior division
68	25827	volume of grey matter in Right Lateral Occipital Cortex, inferior division
69	25828	volume of grey matter in Left Intracalcarine Cortex
70	25829	volume of grey matter in Right Intracalcarine Cortex
71	25830	volume of grey matter in Left Frontal Medial Cortex
72	25831	volume of grey matter in Right Frontal Medial Cortex
73	25832	volume of grey matter in Left Juxtapositional Lobule Cortex (formerly Supplementary Motor Cortex)
74	25833	volume of grey matter in Right Juxtapositional Lobule Cortex (formerly Supplementary Motor Cortex)
75	25834	volume of grey matter in Left Subcallosal Cortex
76	25835	volume of grey matter in Right Subcallosal Cortex
77	25836	volume of grey matter in Left Paracingulate Gyrus
78	25837	volume of grey matter in Right Paracingulate Gyrus
79	25838	volume of grey matter in Left Cingulate Gyrus, anterior division
80	25839	volume of grey matter in Right Cingulate Gyrus, anterior division
81	25840	volume of grey matter in Left Cingulate Gyrus, posterior division
82	25841	volume of grey matter in Right Cingulate Gyrus, posterior division
83	25842	volume of grey matter in Left Precuneous Cortex
84	25843	volume of grey matter in Right Precuneous Cortex

85	25844	volume of grey matter in Left Cuneal Cortex
86	25845	volume of grey matter in Right Cuneal Cortex
87	25846	volume of grey matter in Left Frontal Orbital Cortex
88	25847	volume of grey matter in Right Frontal Orbital Cortex
89	25848	volume of grey matter in Left Parahippocampal Gyrus, anterior division
90	25849	volume of grey matter in Right Parahippocampal Gyrus, anterior division
91	25850	volume of grey matter in Left Parahippocampal Gyrus, posterior division
92	25851	volume of grey matter in Right Parahippocampal Gyrus, posterior division
93	25852	volume of grey matter in Left Lingual Gyrus
94	25853	volume of grey matter in Right Lingual Gyrus
95	25854	volume of grey matter in Left Temporal Fusiform Cortex, anterior division
96	25855	volume of grey matter in Right Temporal Fusiform Cortex, anterior division
97	25856	volume of grey matter in Left Temporal Fusiform Cortex, posterior division
98	25857	volume of grey matter in Right Temporal Fusiform Cortex, posterior division
99	25858	volume of grey matter in Left Temporal Occipital Fusiform Cortex
100	25859	volume of grey matter in Right Temporal Occipital Fusiform Cortex
101	25860	volume of grey matter in Left Occipital Fusiform Gyrus
102	25861	volume of grey matter in Right Occipital Fusiform Gyrus
103	25862	volume of grey matter in Left Frontal Operculum Cortex
104	25863	volume of grey matter in Right Frontal Operculum Cortex
105	25864	volume of grey matter in Left Central Opercular Cortex
106	25865	volume of grey matter in Right Central Opercular Cortex
107	25866	volume of grey matter in Left Parietal Operculum Cortex
108	25867	volume of grey matter in Right Parietal Operculum Cortex
109	25868	volume of grey matter in Left Planum Polare
110	25869	volume of grey matter in Right Planum Polare
111	25870	volume of grey matter in Left Heschl's Gyrus (includes H1 and H2)
112	25871	volume of grey matter in Right Heschl's Gyrus (includes H1 and H2)
113	25872	volume of grey matter in Left Planum Temporale
114	25873	volume of grey matter in Right Planum Temporale
115	25874	volume of grey matter in Left Supracalcarine Cortex
116	25875	volume of grey matter in Right Supracalcarine Cortex
117	25876	volume of grey matter in Left Occipital Pole
118	25877	volume of grey matter in Right Occipital Pole
119	25878	volume of grey matter in Left Thalamus
120	25879	volume of grey matter in Right Thalamus
121	25880	volume of grey matter in Left Caudate
122	25881	volume of grey matter in Right Caudate
123	25882	volume of grey matter in Left Putamen
124	25883	volume of grey matter in Right Putamen
125	25884	volume of grey matter in Left Pallidum
126	25885	volume of grey matter in Right Pallidum
127	25886	volume of grey matter in Left Hippocampus
128	25887	volume of grey matter in Right Hippocampus
129	25888	volume of grey matter in Left Amygdala
130	25889	volume of grey matter in Right Amygdala
131	25890	volume of grey matter in Left Ventral Striatum
132	25891	volume of grey matter in Right Ventral Striatum
133	25892	volume of grey matter in Brain-Stem
134	25893	volume of grey matter in Left I-IV Cerebellum
135	25894	volume of grey matter in Right I-IV Cerebellum
136	25895	volume of grey matter in Left V Cerebellum
137	25896	volume of grey matter in Right V Cerebellum
138	25897	volume of grey matter in Left VI Cerebellum
139	25898	volume of grey matter in Vermis VI Cerebellum
140	25899	volume of grey matter in Right VI Cerebellum
141	25900	volume of grey matter in Left Crus I Cerebellum
142	25901	volume of grey matter in Vermis Crus I Cerebellum
143	25902	volume of grey matter in Right Crus I Cerebellum
144	25903	volume of grey matter in Left Crus II Cerebellum
145	25904	volume of grey matter in Vermis Crus II Cerebellum
146	25905	volume of grey matter in Right Crus II Cerebellum
147	25906	volume of grey matter in Left VIIb Cerebellum
148	25907	volume of grey matter in Vermis VIIb Cerebellum
149	25908	volume of grey matter in Right VIIb Cerebellum
150	25909	volume of grey matter in Left VIIIa Cerebellum
151	25910	volume of grey matter in Vermis VIIIa Cerebellum
152	25911	volume of grey matter in Right VIIIa Cerebellum
153	25912	volume of grey matter in Left VIIIb Cerebellum
154	25913	volume of grey matter in Vermis VIIIb Cerebellum
155	25914	volume of grey matter in Right VIIIb Cerebellum
156	25915	volume of grey matter in Left IX Cerebellum
157	25916	volume of grey matter in Vermis IX Cerebellum
158	25917	volume of grey matter in Right IX Cerebellum
159	25918	volume of grey matter in Left X Cerebellum
160	25919	volume of grey matter in Vermis X Cerebellum
161	25920	volume of grey matter in Right X Cerebellum
162	25026	Median T2star in left thalamus (from SWI data)
163	25027	Median T2star in right thalamus (from SWI data)
164	25028	Median T2star in left caudate (from SWI data)
165	25029	Median T2star in right caudate (from SWI data)
166	25030	Median T2star in left putamen (from SWI data)
167	25031	Median T2star in right putamen (from SWI data)
168	25032	Median T2star in left pallidum (from SWI data)
169	25033	Median T2star in right pallidum (from SWI data)
170	25034	Median T2star in left hippocampus (from SWI data)
171	25035	Median T2star in right hippocampus (from SWI data)
172	25036	Median T2star in left amygdala (from SWI data)
173	25037	Median T2star in right amygdala (from SWI data)

174	25038	Median T2star in left accumbens (from SWI data)
175	25039	Median T2star in right accumbens (from SWI data)
176	26504	Mean intensity of Brain-Stem in the whole brain generated by subcortical volumetric segmentation (aseg)
177	26507	Mean intensity of non-WM-hypointensities in the whole brain generated by subcortical volumetric segmentation (aseg)
178	26508	Mean intensity of Optic-Chiasm in the whole brain generated by subcortical volumetric segmentation (aseg)
179	26509	Mean intensity of CC-Posterior in the whole brain generated by subcortical volumetric segmentation (aseg)
180	26510	Mean intensity of CC-Mid-Posterior in the whole brain generated by subcortical volumetric segmentation (aseg)
181	26511	Mean intensity of CC-Central in the whole brain generated by subcortical volumetric segmentation (aseg)
182	26512	Mean intensity of CC-Mid-Anterior in the whole brain generated by subcortical volumetric segmentation (aseg)
183	26513	Mean intensity of CC-Anterior in the whole brain generated by subcortical volumetric segmentation (aseg)
184	26514	Volume of BrainSeg in the whole brain generated by subcortical volumetric segmentation (aseg)
185	26515	Volume of BrainSegNotVent in the whole brain generated by subcortical volumetric segmentation (aseg)
186	26516	Volume of BrainSegNotVentSurf in the whole brain generated by subcortical volumetric segmentation (aseg)
187	26517	Volume of SubCortGray in the whole brain generated by subcortical volumetric segmentation (aseg)
188	26518	Volume of TotalGray in the whole brain generated by subcortical volumetric segmentation (aseg)
189	26519	Volume of SupraTentorial in the whole brain generated by subcortical volumetric segmentation (aseg)
190	26520	Volume of SupraTentorialNotVent in the whole brain generated by subcortical volumetric segmentation (aseg)
191	26521	Volume of EstimatedTotalIntraCranial in the whole brain generated by subcortical volumetric segmentation (aseg)
192	26526	Volume of Brain-Stem in the whole brain generated by subcortical volumetric segmentation (aseg)
193	26529	Volume of non-WM-hypointensities in the whole brain generated by subcortical volumetric segmentation (aseg)
194	26530	Volume of Optic-Chiasm in the whole brain generated by subcortical volumetric segmentation (aseg)
195	26531	Volume of CC-Posterior in the whole brain generated by subcortical volumetric segmentation (aseg)
196	26532	Volume of CC-Mid-Posterior in the whole brain generated by subcortical volumetric segmentation (aseg)
197	26533	Volume of CC-Central in the whole brain generated by subcortical volumetric segmentation (aseg)
198	26534	Volume of CC-Mid-Anterior in the whole brain generated by subcortical volumetric segmentation (aseg)
199	26535	Volume of CC-Anterior in the whole brain generated by subcortical volumetric segmentation (aseg)
200	26536	Volume-ratio of BrainSegVol-to-eTIV in the whole brain generated by subcortical volumetric segmentation (aseg)
201	26537	Volume-ratio of MaskVol-to-eTIV in the whole brain generated by subcortical volumetric segmentation (aseg)
202	26541	Mean intensity of Cerebellum-Cortex in the left hemisphere generated by subcortical volumetric segmentation (aseg)
203	26542	Mean intensity of Thalamus-Proper in the left hemisphere generated by subcortical volumetric segmentation (aseg)
204	26543	Mean intensity of Caudate in the left hemisphere generated by subcortical volumetric segmentation (aseg)
205	26544	Mean intensity of Putamen in the left hemisphere generated by subcortical volumetric segmentation (aseg)
206	26545	Mean intensity of Pallidum in the left hemisphere generated by subcortical volumetric segmentation (aseg)
207	26546	Mean intensity of Hippocampus in the left hemisphere generated by subcortical volumetric segmentation (aseg)
208	26547	Mean intensity of Amygdala in the left hemisphere generated by subcortical volumetric segmentation (aseg)
209	26548	Mean intensity of Accumbens-area in the left hemisphere generated by subcortical volumetric segmentation (aseg)
210	26549	Mean intensity of VentralDC in the left hemisphere generated by subcortical volumetric segmentation (aseg)
211	26550	Mean intensity of vessel in the left hemisphere generated by subcortical volumetric segmentation (aseg)
212	26551	Mean intensity of choroid-plexus in the left hemisphere generated by subcortical volumetric segmentation (aseg)
213	26552	Volume of Cortex in the left hemisphere generated by subcortical volumetric segmentation (aseg)
214	26557	Volume of Cerebellum-Cortex in the left hemisphere generated by subcortical volumetric segmentation (aseg)
215	26558	Volume of Thalamus-Proper in the left hemisphere generated by subcortical volumetric segmentation (aseg)
216	26559	Volume of Caudate in the left hemisphere generated by subcortical volumetric segmentation (aseg)
217	26560	Volume of Putamen in the left hemisphere generated by subcortical volumetric segmentation (aseg)
218	26561	Volume of Pallidum in the left hemisphere generated by subcortical volumetric segmentation (aseg)
219	26562	Volume of Hippocampus in the left hemisphere generated by subcortical volumetric segmentation (aseg)
220	26563	Volume of Amygdala in the left hemisphere generated by subcortical volumetric segmentation (aseg)
221	26564	Volume of Accumbens-area in the left hemisphere generated by subcortical volumetric segmentation (aseg)
222	26565	Volume of VentralDC in the left hemisphere generated by subcortical volumetric segmentation (aseg)
223	26566	Volume of vessel in the left hemisphere generated by subcortical volumetric segmentation (aseg)
224	26567	Volume of choroid-plexus in the left hemisphere generated by subcortical volumetric segmentation (aseg)
225	26568	Number of HolesBeforeFixing in the left hemisphere generated by subcortical volumetric segmentation (aseg)
226	26572	Mean intensity of Cerebellum-Cortex in the right hemisphere generated by subcortical volumetric segmentation (aseg)
227	26573	Mean intensity of Thalamus-Proper in the right hemisphere generated by subcortical volumetric segmentation (aseg)
228	26574	Mean intensity of Caudate in the right hemisphere generated by subcortical volumetric segmentation (aseg)
229	26575	Mean intensity of Putamen in the right hemisphere generated by subcortical volumetric segmentation (aseg)
230	26576	Mean intensity of Pallidum in the right hemisphere generated by subcortical volumetric segmentation (aseg)
231	26577	Mean intensity of Hippocampus in the right hemisphere generated by subcortical volumetric segmentation (aseg)
232	26578	Mean intensity of Amygdala in the right hemisphere generated by subcortical volumetric segmentation (aseg)
233	26579	Mean intensity of Accumbens-area in the right hemisphere generated by subcortical volumetric segmentation (aseg)
234	26580	Mean intensity of VentralDC in the right hemisphere generated by subcortical volumetric segmentation (aseg)
235	26581	Mean intensity of vessel in the right hemisphere generated by subcortical volumetric segmentation (aseg)
236	26582	Mean intensity of choroid-plexus in the right hemisphere generated by subcortical volumetric segmentation (aseg)
237	26583	Volume of Cortex in the right hemisphere generated by subcortical volumetric segmentation (aseg)
238	26588	Volume of Cerebellum-Cortex in the right hemisphere generated by subcortical volumetric segmentation (aseg)
239	26589	Volume of Thalamus-Proper in the right hemisphere generated by subcortical volumetric segmentation (aseg)
240	26590	Volume of Caudate in the right hemisphere generated by subcortical volumetric segmentation (aseg)
241	26591	Volume of Putamen in the right hemisphere generated by subcortical volumetric segmentation (aseg)
242	26592	Volume of Pallidum in the right hemisphere generated by subcortical volumetric segmentation (aseg)
243	26593	Volume of Hippocampus in the right hemisphere generated by subcortical volumetric segmentation (aseg)
244	26594	Volume of Amygdala in the right hemisphere generated by subcortical volumetric segmentation (aseg)
245	26595	Volume of Accumbens-area in the right hemisphere generated by subcortical volumetric segmentation (aseg)
246	26596	Volume of VentralDC in the right hemisphere generated by subcortical volumetric segmentation (aseg)
247	26597	Volume of vessel in the right hemisphere generated by subcortical volumetric segmentation (aseg)
248	26598	Volume of choroid-plexus in the right hemisphere generated by subcortical volumetric segmentation (aseg)
249	26599	Number of HolesBeforeFixing in the right hemisphere generated by subcortical volumetric segmentation (aseg)
250	26600	Volume of Lateral-nucleus in the left hemisphere generated by subcortical volumetric sub-segmentation of the Amygdala Nuclei
251	26601	Volume of Basal-nucleus in the left hemisphere generated by subcortical volumetric sub-segmentation of the Amygdala Nuclei
252	26602	Volume of Accessory-Basal-nucleus in the left hemisphere generated by subcortical volumetric sub-segmentation of the Amygdala Nuclei
253	26603	Volume of Anterior-amygdaloid-area-AAA in the left hemisphere generated by subcortical volumetric sub-segmentation of the Amygdala Nuclei
254	26604	Volume of Central-nucleus in the left hemisphere generated by subcortical volumetric sub-segmentation of the Amygdala Nuclei
255	26605	Volume of Medial-nucleus in the left hemisphere generated by subcortical volumetric sub-segmentation of the Amygdala Nuclei
256	26606	Volume of Cortical-nucleus in the left hemisphere generated by subcortical volumetric sub-segmentation of the Amygdala Nuclei
257	26607	Volume of Corticoamygdaloid-transitio in the left hemisphere generated by subcortical volumetric sub-segmentation of the Amygdala Nuclei
258	26608	Volume of Paralaminar-nucleus in the left hemisphere generated by subcortical volumetric sub-segmentation of the Amygdala Nuclei
259	26609	Volume of Whole-amygdala in the left hemisphere generated by subcortical volumetric sub-segmentation of the Amygdala Nuclei
260	26610	Volume of Lateral-nucleus in the right hemisphere generated by subcortical volumetric sub-segmentation of the Amygdala Nuclei
261	26611	Volume of Basal-nucleus in the right hemisphere generated by subcortical volumetric sub-segmentation of the Amygdala Nuclei
262	26612	Volume of Accessory-Basal-nucleus in the right hemisphere generated by subcortical volumetric sub-segmentation of the Amygdala Nuclei

263	26613	Volume of Anterior-amygdaloid-area-AAA in the right hemisphere generated by subcortical volumetric sub-segmentation of the Amygdala Nuclei
264	26614	Volume of Central-nucleus in the right hemisphere generated by subcortical volumetric sub-segmentation of the Amygdala Nuclei
265	26615	Volume of Medial-nucleus in the right hemisphere generated by subcortical volumetric sub-segmentation of the Amygdala Nuclei
266	26616	Volume of Cortical-nucleus in the right hemisphere generated by subcortical volumetric sub-segmentation of the Amygdala Nuclei
267	26617	Volume of Corticoamygdaloid-transitio in the right hemisphere generated by subcortical volumetric sub-segmentation of the Amygdala Nuclei
268	26618	Volume of Paralaminar-nucleus in the right hemisphere generated by subcortical volumetric sub-segmentation of the Amygdala Nuclei
269	26619	Volume of Whole-amygdala in the right hemisphere generated by subcortical volumetric sub-segmentation of the Amygdala Nuclei
270	26620	Volume of Hippocampal-tail in the left hemisphere generated by subcortical volumetric sub-segmentation of the Hippocampal Subfields
271	26621	Volume of subiculum-body in the left hemisphere generated by subcortical volumetric sub-segmentation of the Hippocampal Subfields
272	26622	Volume of CA1-body in the left hemisphere generated by subcortical volumetric sub-segmentation of the Hippocampal Subfields
273	26623	Volume of subiculum-head in the left hemisphere generated by subcortical volumetric sub-segmentation of the Hippocampal Subfields
274	26624	Volume of hippocampal-fissure in the left hemisphere generated by subcortical volumetric sub-segmentation of the Hippocampal Subfields
275	26625	Volume of presubiculum-head in the left hemisphere generated by subcortical volumetric sub-segmentation of the Hippocampal Subfields
276	26626	Volume of CA1-head in the left hemisphere generated by subcortical volumetric sub-segmentation of the Hippocampal Subfields
277	26627	Volume of presubiculum-body in the left hemisphere generated by subcortical volumetric sub-segmentation of the Hippocampal Subfields
278	26628	Volume of parasubiculum in the left hemisphere generated by subcortical volumetric sub-segmentation of the Hippocampal Subfields
279	26629	Volume of molecular-layer-HP-head in the left hemisphere generated by subcortical volumetric sub-segmentation of the Hippocampal Subfields
280	26630	Volume of molecular-layer-HP-body in the left hemisphere generated by subcortical volumetric sub-segmentation of the Hippocampal Subfields
281	26631	Volume of GC-ML-DG-head in the left hemisphere generated by subcortical volumetric sub-segmentation of the Hippocampal Subfields
282	26632	Volume of CA3-body in the left hemisphere generated by subcortical volumetric sub-segmentation of the Hippocampal Subfields
283	26633	Volume of GC-ML-DG-body in the left hemisphere generated by subcortical volumetric sub-segmentation of the Hippocampal Subfields
284	26634	Volume of CA4-head in the left hemisphere generated by subcortical volumetric sub-segmentation of the Hippocampal Subfields
285	26635	Volume of CA4-body in the left hemisphere generated by subcortical volumetric sub-segmentation of the Hippocampal Subfields
286	26636	Volume of fimbria in the left hemisphere generated by subcortical volumetric sub-segmentation of the Hippocampal Subfields
287	26637	Volume of CA3-head in the left hemisphere generated by subcortical volumetric sub-segmentation of the Hippocampal Subfields
288	26638	Volume of HATA in the left hemisphere generated by subcortical volumetric sub-segmentation of the Hippocampal Subfields
289	26639	Volume of Whole-hippocampal-body in the left hemisphere generated by subcortical volumetric sub-segmentation of the Hippocampal Subfields
290	26640	Volume of Whole-hippocampal-head in the left hemisphere generated by subcortical volumetric sub-segmentation of the Hippocampal Subfields
291	26641	Volume of Whole-hippocampus in the left hemisphere generated by subcortical volumetric sub-segmentation of the Hippocampal Subfields
292	26642	Volume of Hippocampal-tail in the right hemisphere generated by subcortical volumetric sub-segmentation of the Hippocampal Subfields
293	26643	Volume of subiculum-body in the right hemisphere generated by subcortical volumetric sub-segmentation of the Hippocampal Subfields
294	26644	Volume of CA1-body in the right hemisphere generated by subcortical volumetric sub-segmentation of the Hippocampal Subfields
295	26645	Volume of subiculum-head in the right hemisphere generated by subcortical volumetric sub-segmentation of the Hippocampal Subfields
296	26646	Volume of hippocampal-fissure in the right hemisphere generated by subcortical volumetric sub-segmentation of the Hippocampal Subfields
297	26647	Volume of presubiculum-head in the right hemisphere generated by subcortical volumetric sub-segmentation of the Hippocampal Subfields
298	26648	Volume of CA1-head in the right hemisphere generated by subcortical volumetric sub-segmentation of the Hippocampal Subfields
299	26649	Volume of presubiculum-body in the right hemisphere generated by subcortical volumetric sub-segmentation of the Hippocampal Subfields
300	26650	Volume of parasubiculum in the right hemisphere generated by subcortical volumetric sub-segmentation of the Hippocampal Subfields
301	26651	Volume of molecular-layer-HP-head in the right hemisphere generated by subcortical volumetric sub-segmentation of the Hippocampal Subfields
302	26652	Volume of molecular-layer-HP-body in the right hemisphere generated by subcortical volumetric sub-segmentation of the Hippocampal Subfields
303	26653	Volume of GC-ML-DG-head in the right hemisphere generated by subcortical volumetric sub-segmentation of the Hippocampal Subfields
304	26654	Volume of CA3-body in the right hemisphere generated by subcortical volumetric sub-segmentation of the Hippocampal Subfields
305	26655	Volume of GC-ML-DG-body in the right hemisphere generated by subcortical volumetric sub-segmentation of the Hippocampal Subfields
306	26656	Volume of CA4-head in the right hemisphere generated by subcortical volumetric sub-segmentation of the Hippocampal Subfields
307	26657	Volume of CA4-body in the right hemisphere generated by subcortical volumetric sub-segmentation of the Hippocampal Subfields
308	26658	Volume of fimbria in the right hemisphere generated by subcortical volumetric sub-segmentation of the Hippocampal Subfields
309	26659	Volume of CA3-head in the right hemisphere generated by subcortical volumetric sub-segmentation of the Hippocampal Subfields
310	26660	Volume of HATA in the right hemisphere generated by subcortical volumetric sub-segmentation of the Hippocampal Subfields
311	26661	Volume of Whole-hippocampal-body in the right hemisphere generated by subcortical volumetric sub-segmentation of the Hippocampal Subfields
312	26662	Volume of Whole-hippocampal-head in the right hemisphere generated by subcortical volumetric sub-segmentation of the Hippocampal Subfields
313	26663	Volume of Whole-hippocampus in the right hemisphere generated by subcortical volumetric sub-segmentation of the Hippocampal Subfields
314	26664	Volume of MGN in the left hemisphere generated by subcortical volumetric sub-segmentation of the Thalamic Nuclei
315	26665	Volume of LGN in the left hemisphere generated by subcortical volumetric sub-segmentation of the Thalamic Nuclei
316	26666	Volume of PuI in the left hemisphere generated by subcortical volumetric sub-segmentation of the Thalamic Nuclei
317	26667	Volume of PuM in the left hemisphere generated by subcortical volumetric sub-segmentation of the Thalamic Nuclei
318	26668	Volume of L-Sg in the left hemisphere generated by subcortical volumetric sub-segmentation of the Thalamic Nuclei
319	26669	Volume of VPL in the left hemisphere generated by subcortical volumetric sub-segmentation of the Thalamic Nuclei
320	26670	Volume of CM in the left hemisphere generated by subcortical volumetric sub-segmentation of the Thalamic Nuclei
321	26671	Volume of VLa in the left hemisphere generated by subcortical volumetric sub-segmentation of the Thalamic Nuclei
322	26672	Volume of PuA in the left hemisphere generated by subcortical volumetric sub-segmentation of the Thalamic Nuclei
323	26673	Volume of MDm in the left hemisphere generated by subcortical volumetric sub-segmentation of the Thalamic Nuclei
324	26674	Volume of Pf in the left hemisphere generated by subcortical volumetric sub-segmentation of the Thalamic Nuclei
325	26675	Volume of VAmc in the left hemisphere generated by subcortical volumetric sub-segmentation of the Thalamic Nuclei
326	26676	Volume of MDl in the left hemisphere generated by subcortical volumetric sub-segmentation of the Thalamic Nuclei
327	26677	Volume of CeM in the left hemisphere generated by subcortical volumetric sub-segmentation of the Thalamic Nuclei
328	26678	Volume of VA in the left hemisphere generated by subcortical volumetric sub-segmentation of the Thalamic Nuclei
329	26679	Volume of MV(Re) in the left hemisphere generated by subcortical volumetric sub-segmentation of the Thalamic Nuclei
330	26680	Volume of VM in the left hemisphere generated by subcortical volumetric sub-segmentation of the Thalamic Nuclei
331	26681	Volume of CL in the left hemisphere generated by subcortical volumetric sub-segmentation of the Thalamic Nuclei
332	26682	Volume of PuL in the left hemisphere generated by subcortical volumetric sub-segmentation of the Thalamic Nuclei
333	26683	Volume of Pt in the left hemisphere generated by subcortical volumetric sub-segmentation of the Thalamic Nuclei
334	26684	Volume of AV in the left hemisphere generated by subcortical volumetric sub-segmentation of the Thalamic Nuclei
335	26685	Volume of Pc in the left hemisphere generated by subcortical volumetric sub-segmentation of the Thalamic Nuclei
336	26686	Volume of VLp in the left hemisphere generated by subcortical volumetric sub-segmentation of the Thalamic Nuclei
337	26687	Volume of LP in the left hemisphere generated by subcortical volumetric sub-segmentation of the Thalamic Nuclei
338	26688	Volume of LGN in the right hemisphere generated by subcortical volumetric sub-segmentation of the Thalamic Nuclei
339	26689	Volume of MGN in the right hemisphere generated by subcortical volumetric sub-segmentation of the Thalamic Nuclei
340	26690	Volume of PuI in the right hemisphere generated by subcortical volumetric sub-segmentation of the Thalamic Nuclei
341	26691	Volume of PuM in the right hemisphere generated by subcortical volumetric sub-segmentation of the Thalamic Nuclei
342	26692	Volume of L-Sg in the right hemisphere generated by subcortical volumetric sub-segmentation of the Thalamic Nuclei
343	26693	Volume of VPL in the right hemisphere generated by subcortical volumetric sub-segmentation of the Thalamic Nuclei
344	26694	Volume of CM in the right hemisphere generated by subcortical volumetric sub-segmentation of the Thalamic Nuclei
345	26695	Volume of VLa in the right hemisphere generated by subcortical volumetric sub-segmentation of the Thalamic Nuclei
346	26696	Volume of PuA in the right hemisphere generated by subcortical volumetric sub-segmentation of the Thalamic Nuclei
347	26697	Volume of MDm in the right hemisphere generated by subcortical volumetric sub-segmentation of the Thalamic Nuclei
348	26698	Volume of Pf in the right hemisphere generated by subcortical volumetric sub-segmentation of the Thalamic Nuclei
349	26699	Volume of VAmc in the right hemisphere generated by subcortical volumetric sub-segmentation of the Thalamic Nuclei
350	26700	Volume of MDl in the right hemisphere generated by subcortical volumetric sub-segmentation of the Thalamic Nuclei
351	26701	Volume of VA in the right hemisphere generated by subcortical volumetric sub-segmentation of the Thalamic Nuclei

352	26702	Volume of MV(Re) in the right hemisphere generated by subcortical volumetric sub-segmentation of the Thalamic Nuclei
353	26703	Volume of CeM in the right hemisphere generated by subcortical volumetric sub-segmentation of the Thalamic Nuclei
354	26704	Volume of VM in the right hemisphere generated by subcortical volumetric sub-segmentation of the Thalamic Nuclei
355	26705	Volume of PuL in the right hemisphere generated by subcortical volumetric sub-segmentation of the Thalamic Nuclei
356	26706	Volume of CL in the right hemisphere generated by subcortical volumetric sub-segmentation of the Thalamic Nuclei
357	26707	Volume of VLp in the right hemisphere generated by subcortical volumetric sub-segmentation of the Thalamic Nuclei
358	26708	Volume of Pc in the right hemisphere generated by subcortical volumetric sub-segmentation of the Thalamic Nuclei
359	26709	Volume of Pt in the right hemisphere generated by subcortical volumetric sub-segmentation of the Thalamic Nuclei
360	26710	Volume of AV in the right hemisphere generated by subcortical volumetric sub-segmentation of the Thalamic Nuclei
361	26711	Volume of LP in the right hemisphere generated by subcortical volumetric sub-segmentation of the Thalamic Nuclei
362	26712	Volume of LD in the left hemisphere generated by subcortical volumetric sub-segmentation of the Thalamic Nuclei
363	26713	Volume of LD in the right hemisphere generated by subcortical volumetric sub-segmentation of the Thalamic Nuclei
364	26714	Volume of Whole-thalamus in the left hemisphere generated by subcortical volumetric sub-segmentation of the Thalamic Nuclei
365	26715	Volume of Whole-thalamus in the right hemisphere generated by subcortical volumetric sub-segmentation of the Thalamic Nuclei
366	26716	Volume of Medulla in the whole brain generated by subcortical volumetric sub-segmentation of the Brainstem
367	26717	Volume of Pons in the whole brain generated by subcortical volumetric sub-segmentation of the Brainstem
368	26718	Volume of SCP in the whole brain generated by subcortical volumetric sub-segmentation of the Brainstem
369	26719	Volume of Midbrain in the whole brain generated by subcortical volumetric sub-segmentation of the Brainstem
370	26720	Volume of Whole-brainstem in the whole brain generated by subcortical volumetric sub-segmentation of the Brainstem
371	26721	Area of TotalSurface in the left hemisphere generated by parcellation of the white surface using Desikan-Killiany parcellation
372	26722	Area of bankssts in the left hemisphere generated by parcellation of the white surface using Desikan-Killiany parcellation
373	26723	Area of caudalanteriorcingulate in the left hemisphere generated by parcellation of the white surface using Desikan-Killiany parcellation
374	26724	Area of caudalmiddlefrontal in the left hemisphere generated by parcellation of the white surface using Desikan-Killiany parcellation
375	26725	Area of cuneus in the left hemisphere generated by parcellation of the white surface using Desikan-Killiany parcellation
376	26726	Area of entorhinal in the left hemisphere generated by parcellation of the white surface using Desikan-Killiany parcellation
377	26727	Area of fusiform in the left hemisphere generated by parcellation of the white surface using Desikan-Killiany parcellation
378	26728	Area of inferiorparietal in the left hemisphere generated by parcellation of the white surface using Desikan-Killiany parcellation
379	26729	Area of inferiortemporal in the left hemisphere generated by parcellation of the white surface using Desikan-Killiany parcellation
380	26730	Area of isthmuscingulate in the left hemisphere generated by parcellation of the white surface using Desikan-Killiany parcellation
381	26731	Area of lateraloccipital in the left hemisphere generated by parcellation of the white surface using Desikan-Killiany parcellation
382	26732	Area of lateralorbitofrontal in the left hemisphere generated by parcellation of the white surface using Desikan-Killiany parcellation
383	26733	Area of lingual in the left hemisphere generated by parcellation of the white surface using Desikan-Killiany parcellation
384	26734	Area of medialorbitofrontal in the left hemisphere generated by parcellation of the white surface using Desikan-Killiany parcellation
385	26735	Area of middletemporal in the left hemisphere generated by parcellation of the white surface using Desikan-Killiany parcellation
386	26736	Area of parahippocampal in the left hemisphere generated by parcellation of the white surface using Desikan-Killiany parcellation
387	26737	Area of paracentral in the left hemisphere generated by parcellation of the white surface using Desikan-Killiany parcellation
388	26738	Area of parsopercularis in the left hemisphere generated by parcellation of the white surface using Desikan-Killiany parcellation
389	26739	Area of parsorbitalis in the left hemisphere generated by parcellation of the white surface using Desikan-Killiany parcellation
390	26740	Area of parstriangularis in the left hemisphere generated by parcellation of the white surface using Desikan-Killiany parcellation
391	26741	Area of pericalcarine in the left hemisphere generated by parcellation of the white surface using Desikan-Killiany parcellation
392	26742	Area of postcentral in the left hemisphere generated by parcellation of the white surface using Desikan-Killiany parcellation
393	26743	Area of posteriorcingulate in the left hemisphere generated by parcellation of the white surface using Desikan-Killiany parcellation
394	26744	Area of precentral in the left hemisphere generated by parcellation of the white surface using Desikan-Killiany parcellation
395	26745	Area of precuneus in the left hemisphere generated by parcellation of the white surface using Desikan-Killiany parcellation
396	26746	Area of rostralanteriorcingulate in the left hemisphere generated by parcellation of the white surface using Desikan-Killiany parcellation
397	26747	Area of rostralmiddlefrontal in the left hemisphere generated by parcellation of the white surface using Desikan-Killiany parcellation
398	26748	Area of superiorfrontal in the left hemisphere generated by parcellation of the white surface using Desikan-Killiany parcellation
399	26749	Area of superiorparietal in the left hemisphere generated by parcellation of the white surface using Desikan-Killiany parcellation
400	26750	Area of superiortemporal in the left hemisphere generated by parcellation of the white surface using Desikan-Killiany parcellation
401	26751	Area of supramarginal in the left hemisphere generated by parcellation of the white surface using Desikan-Killiany parcellation
402	26752	Area of frontalpole in the left hemisphere generated by parcellation of the white surface using Desikan-Killiany parcellation
403	26753	Area of transversetemporal in the left hemisphere generated by parcellation of the white surface using Desikan-Killiany parcellation
404	26754	Area of insula in the left hemisphere generated by parcellation of the white surface using Desikan-Killiany parcellation
405	26755	Mean thickness of GlobalMeanMean thickness in the left hemisphere generated by parcellation of the white surface using Desikan-Killiany parcellation
406	26756	Mean thickness of bankssts in the left hemisphere generated by parcellation of the white surface using Desikan-Killiany parcellation
407	26757	Mean thickness of caudalanteriorcingulate in the left hemisphere generated by parcellation of the white surface using Desikan-Killiany parcellation
408	26758	Mean thickness of caudalmiddlefrontal in the left hemisphere generated by parcellation of the white surface using Desikan-Killiany parcellation
409	26759	Mean thickness of cuneus in the left hemisphere generated by parcellation of the white surface using Desikan-Killiany parcellation
410	26760	Mean thickness of entorhinal in the left hemisphere generated by parcellation of the white surface using Desikan-Killiany parcellation
411	26761	Mean thickness of fusiform in the left hemisphere generated by parcellation of the white surface using Desikan-Killiany parcellation
412	26762	Mean thickness of inferiorparietal in the left hemisphere generated by parcellation of the white surface using Desikan-Killiany parcellation
413	26763	Mean thickness of inferiortemporal in the left hemisphere generated by parcellation of the white surface using Desikan-Killiany parcellation
414	26764	Mean thickness of isthmuscingulate in the left hemisphere generated by parcellation of the white surface using Desikan-Killiany parcellation
415	26765	Mean thickness of lateraloccipital in the left hemisphere generated by parcellation of the white surface using Desikan-Killiany parcellation
416	26766	Mean thickness of lateralorbitofrontal in the left hemisphere generated by parcellation of the white surface using Desikan-Killiany parcellation
417	26767	Mean thickness of lingual in the left hemisphere generated by parcellation of the white surface using Desikan-Killiany parcellation
418	26768	Mean thickness of medialorbitofrontal in the left hemisphere generated by parcellation of the white surface using Desikan-Killiany parcellation
419	26769	Mean thickness of middletemporal in the left hemisphere generated by parcellation of the white surface using Desikan-Killiany parcellation
420	26770	Mean thickness of parahippocampal in the left hemisphere generated by parcellation of the white surface using Desikan-Killiany parcellation
421	26771	Mean thickness of paracentral in the left hemisphere generated by parcellation of the white surface using Desikan-Killiany parcellation
422	26772	Mean thickness of parsopercularis in the left hemisphere generated by parcellation of the white surface using Desikan-Killiany parcellation
423	26773	Mean thickness of parsorbitalis in the left hemisphere generated by parcellation of the white surface using Desikan-Killiany parcellation
424	26774	Mean thickness of parstriangularis in the left hemisphere generated by parcellation of the white surface using Desikan-Killiany parcellation
425	26775	Mean thickness of pericalcarine in the left hemisphere generated by parcellation of the white surface using Desikan-Killiany parcellation
426	26776	Mean thickness of postcentral in the left hemisphere generated by parcellation of the white surface using Desikan-Killiany parcellation
427	26777	Mean thickness of posteriorcingulate in the left hemisphere generated by parcellation of the white surface using Desikan-Killiany parcellation
428	26778	Mean thickness of precentral in the left hemisphere generated by parcellation of the white surface using Desikan-Killiany parcellation
429	26779	Mean thickness of precuneus in the left hemisphere generated by parcellation of the white surface using Desikan-Killiany parcellation
430	26780	Mean thickness of rostralanteriorcingulate in the left hemisphere generated by parcellation of the white surface using Desikan-Killiany parcellation
431	26781	Mean thickness of rostralmiddlefrontal in the left hemisphere generated by parcellation of the white surface using Desikan-Killiany parcellation
432	26782	Mean thickness of superiorfrontal in the left hemisphere generated by parcellation of the white surface using Desikan-Killiany parcellation
433	26783	Mean thickness of superiorparietal in the left hemisphere generated by parcellation of the white surface using Desikan-Killiany parcellation
434	26784	Mean thickness of superiortemporal in the left hemisphere generated by parcellation of the white surface using Desikan-Killiany parcellation
435	26785	Mean thickness of supramarginal in the left hemisphere generated by parcellation of the white surface using Desikan-Killiany parcellation
436	26786	Mean thickness of frontalpole in the left hemisphere generated by parcellation of the white surface using Desikan-Killiany parcellation
437	26787	Mean thickness of transversetemporal in the left hemisphere generated by parcellation of the white surface using Desikan-Killiany parcellation
438	26788	Mean thickness of insula in the left hemisphere generated by parcellation of the white surface using Desikan-Killiany parcellation
439	26789	Volume of bankssts in the left hemisphere generated by parcellation of the white surface using Desikan-Killiany parcellation
440	26790	Volume of caudalanteriorcingulate in the left hemisphere generated by parcellation of the white surface using Desikan-Killiany parcellation

441	26791	Volume of caudalmiddlefrontal in the left hemisphere generated by parcellation of the white surface using Desikan-Killiany parcellation
442	26792	Volume of cuneus in the left hemisphere generated by parcellation of the white surface using Desikan-Killiany parcellation
443	26793	Volume of entorhinal in the left hemisphere generated by parcellation of the white surface using Desikan-Killiany parcellation
444	26794	Volume of fusiform in the left hemisphere generated by parcellation of the white surface using Desikan-Killiany parcellation
445	26795	Volume of inferiorparietal in the left hemisphere generated by parcellation of the white surface using Desikan-Killiany parcellation
446	26796	Volume of inferiortemporal in the left hemisphere generated by parcellation of the white surface using Desikan-Killiany parcellation
447	26797	Volume of isthmuscingulate in the left hemisphere generated by parcellation of the white surface using Desikan-Killiany parcellation
448	26798	Volume of lateraloccipital in the left hemisphere generated by parcellation of the white surface using Desikan-Killiany parcellation
449	26799	Volume of lateralorbitofrontal in the left hemisphere generated by parcellation of the white surface using Desikan-Killiany parcellation
450	26800	Volume of lingual in the left hemisphere generated by parcellation of the white surface using Desikan-Killiany parcellation
451	26801	Volume of medialorbitofrontal in the left hemisphere generated by parcellation of the white surface using Desikan-Killiany parcellation
452	26802	Volume of middletemporal in the left hemisphere generated by parcellation of the white surface using Desikan-Killiany parcellation
453	26803	Volume of parahippocampal in the left hemisphere generated by parcellation of the white surface using Desikan-Killiany parcellation
454	26804	Volume of paracentral in the left hemisphere generated by parcellation of the white surface using Desikan-Killiany parcellation
455	26805	Volume of parsopercularis in the left hemisphere generated by parcellation of the white surface using Desikan-Killiany parcellation
456	26806	Volume of parsorbitalis in the left hemisphere generated by parcellation of the white surface using Desikan-Killiany parcellation
457	26807	Volume of parstriangularis in the left hemisphere generated by parcellation of the white surface using Desikan-Killiany parcellation
458	26808	Volume of pericalcarine in the left hemisphere generated by parcellation of the white surface using Desikan-Killiany parcellation
459	26809	Volume of postcentral in the left hemisphere generated by parcellation of the white surface using Desikan-Killiany parcellation
460	26810	Volume of posteriorcingulate in the left hemisphere generated by parcellation of the white surface using Desikan-Killiany parcellation
461	26811	Volume of precentral in the left hemisphere generated by parcellation of the white surface using Desikan-Killiany parcellation
462	26812	Volume of precuneus in the left hemisphere generated by parcellation of the white surface using Desikan-Killiany parcellation
463	26813	Volume of rostralanteriorcingulate in the left hemisphere generated by parcellation of the white surface using Desikan-Killiany parcellation
464	26814	Volume of rostralmiddlefrontal in the left hemisphere generated by parcellation of the white surface using Desikan-Killiany parcellation
465	26815	Volume of superiorfrontal in the left hemisphere generated by parcellation of the white surface using Desikan-Killiany parcellation
466	26816	Volume of superiorparietal in the left hemisphere generated by parcellation of the white surface using Desikan-Killiany parcellation
467	26817	Volume of superiortemporal in the left hemisphere generated by parcellation of the white surface using Desikan-Killiany parcellation
468	26818	Volume of supramarginal in the left hemisphere generated by parcellation of the white surface using Desikan-Killiany parcellation
469	26819	Volume of frontalpole in the left hemisphere generated by parcellation of the white surface using Desikan-Killiany parcellation
470	26820	Volume of transversetemporal in the left hemisphere generated by parcellation of the white surface using Desikan-Killiany parcellation
471	26821	Volume of insula in the left hemisphere generated by parcellation of the white surface using Desikan-Killiany parcellation
472	26822	Area of TotalSurface in the right hemisphere generated by parcellation of the white surface using Desikan-Killiany parcellation
473	26823	Area of bankssts in the right hemisphere generated by parcellation of the white surface using Desikan-Killiany parcellation
474	26824	Area of caudalanteriorcingulate in the right hemisphere generated by parcellation of the white surface using Desikan-Killiany parcellation
475	26825	Area of caudalmiddlefrontal in the right hemisphere generated by parcellation of the white surface using Desikan-Killiany parcellation
476	26826	Area of cuneus in the right hemisphere generated by parcellation of the white surface using Desikan-Killiany parcellation
477	26827	Area of entorhinal in the right hemisphere generated by parcellation of the white surface using Desikan-Killiany parcellation
478	26828	Area of fusiform in the right hemisphere generated by parcellation of the white surface using Desikan-Killiany parcellation
479	26829	Area of inferiorparietal in the right hemisphere generated by parcellation of the white surface using Desikan-Killiany parcellation
480	26830	Area of inferiortemporal in the right hemisphere generated by parcellation of the white surface using Desikan-Killiany parcellation
481	26831	Area of isthmuscingulate in the right hemisphere generated by parcellation of the white surface using Desikan-Killiany parcellation
482	26832	Area of lateraloccipital in the right hemisphere generated by parcellation of the white surface using Desikan-Killiany parcellation
483	26833	Area of lateralorbitofrontal in the right hemisphere generated by parcellation of the white surface using Desikan-Killiany parcellation
484	26834	Area of lingual in the right hemisphere generated by parcellation of the white surface using Desikan-Killiany parcellation
485	26835	Area of medialorbitofrontal in the right hemisphere generated by parcellation of the white surface using Desikan-Killiany parcellation
486	26836	Area of middletemporal in the right hemisphere generated by parcellation of the white surface using Desikan-Killiany parcellation
487	26837	Area of parahippocampal in the right hemisphere generated by parcellation of the white surface using Desikan-Killiany parcellation
488	26838	Area of paracentral in the right hemisphere generated by parcellation of the white surface using Desikan-Killiany parcellation
489	26839	Area of parsopercularis in the right hemisphere generated by parcellation of the white surface using Desikan-Killiany parcellation
490	26840	Area of parsorbitalis in the right hemisphere generated by parcellation of the white surface using Desikan-Killiany parcellation
491	26841	Area of parstriangularis in the right hemisphere generated by parcellation of the white surface using Desikan-Killiany parcellation
492	26842	Area of pericalcarine in the right hemisphere generated by parcellation of the white surface using Desikan-Killiany parcellation
493	26843	Area of postcentral in the right hemisphere generated by parcellation of the white surface using Desikan-Killiany parcellation
494	26844	Area of posteriorcingulate in the right hemisphere generated by parcellation of the white surface using Desikan-Killiany parcellation
495	26845	Area of precentral in the right hemisphere generated by parcellation of the white surface using Desikan-Killiany parcellation
496	26846	Area of precuneus in the right hemisphere generated by parcellation of the white surface using Desikan-Killiany parcellation
497	26847	Area of rostralanteriorcingulate in the right hemisphere generated by parcellation of the white surface using Desikan-Killiany parcellation
498	26848	Area of rostralmiddlefrontal in the right hemisphere generated by parcellation of the white surface using Desikan-Killiany parcellation
499	26849	Area of superiorfrontal in the right hemisphere generated by parcellation of the white surface using Desikan-Killiany parcellation
500	26850	Area of superiorparietal in the right hemisphere generated by parcellation of the white surface using Desikan-Killiany parcellation
501	26851	Area of superiortemporal in the right hemisphere generated by parcellation of the white surface using Desikan-Killiany parcellation
502	26852	Area of supramarginal in the right hemisphere generated by parcellation of the white surface using Desikan-Killiany parcellation
503	26853	Area of frontalpole in the right hemisphere generated by parcellation of the white surface using Desikan-Killiany parcellation
504	26854	Area of transversetemporal in the right hemisphere generated by parcellation of the white surface using Desikan-Killiany parcellation
505	26855	Area of insula in the right hemisphere generated by parcellation of the white surface using Desikan-Killiany parcellation
506	26856	Mean thickness of GlobalMeanMean thickness in the right hemisphere generated by parcellation of the white surface using Desikan-Killiany parcellation
507	26857	Mean thickness of bankssts in the right hemisphere generated by parcellation of the white surface using Desikan-Killiany parcellation
508	26858	Mean thickness of caudalanteriorcingulate in the right hemisphere generated by parcellation of the white surface using Desikan-Killiany parcellation
509	26859	Mean thickness of caudalmiddlefrontal in the right hemisphere generated by parcellation of the white surface using Desikan-Killiany parcellation
510	26860	Mean thickness of cuneus in the right hemisphere generated by parcellation of the white surface using Desikan-Killiany parcellation
511	26861	Mean thickness of entorhinal in the right hemisphere generated by parcellation of the white surface using Desikan-Killiany parcellation
512	26862	Mean thickness of fusiform in the right hemisphere generated by parcellation of the white surface using Desikan-Killiany parcellation
513	26863	Mean thickness of inferiorparietal in the right hemisphere generated by parcellation of the white surface using Desikan-Killiany parcellation
514	26864	Mean thickness of inferiortemporal in the right hemisphere generated by parcellation of the white surface using Desikan-Killiany parcellation
515	26865	Mean thickness of isthmuscingulate in the right hemisphere generated by parcellation of the white surface using Desikan-Killiany parcellation
516	26866	Mean thickness of lateraloccipital in the right hemisphere generated by parcellation of the white surface using Desikan-Killiany parcellation
517	26867	Mean thickness of lateralorbitofrontal in the right hemisphere generated by parcellation of the white surface using Desikan-Killiany parcellation
518	26868	Mean thickness of lingual in the right hemisphere generated by parcellation of the white surface using Desikan-Killiany parcellation
519	26869	Mean thickness of medialorbitofrontal in the right hemisphere generated by parcellation of the white surface using Desikan-Killiany parcellation
520	26870	Mean thickness of middletemporal in the right hemisphere generated by parcellation of the white surface using Desikan-Killiany parcellation
521	26871	Mean thickness of parahippocampal in the right hemisphere generated by parcellation of the white surface using Desikan-Killiany parcellation
522	26872	Mean thickness of paracentral in the right hemisphere generated by parcellation of the white surface using Desikan-Killiany parcellation
523	26873	Mean thickness of parsopercularis in the right hemisphere generated by parcellation of the white surface using Desikan-Killiany parcellation
524	26874	Mean thickness of parsorbitalis in the right hemisphere generated by parcellation of the white surface using Desikan-Killiany parcellation
525	26875	Mean thickness of parstriangularis in the right hemisphere generated by parcellation of the white surface using Desikan-Killiany parcellation
526	26876	Mean thickness of pericalcarine in the right hemisphere generated by parcellation of the white surface using Desikan-Killiany parcellation
527	26877	Mean thickness of postcentral in the right hemisphere generated by parcellation of the white surface using Desikan-Killiany parcellation
528	26878	Mean thickness of posteriorcingulate in the right hemisphere generated by parcellation of the white surface using Desikan-Killiany parcellation
529	26879	Mean thickness of precentral in the right hemisphere generated by parcellation of the white surface using Desikan-Killiany parcellation

530	26880	Mean thickness of precuneus in the right hemisphere generated by parcellation of the white surface using Desikan-Killiany parcellation
531	26881	Mean thickness of rostralanteriorcingulate in the right hemisphere generated by parcellation of the white surface using Desikan-Killiany parcellation
532	26882	Mean thickness of rostralmiddlefrontal in the right hemisphere generated by parcellation of the white surface using Desikan-Killiany parcellation
533	26883	Mean thickness of superiorfrontal in the right hemisphere generated by parcellation of the white surface using Desikan-Killiany parcellation
534	26884	Mean thickness of superiorparietal in the right hemisphere generated by parcellation of the white surface using Desikan-Killiany parcellation
535	26885	Mean thickness of superiortemporal in the right hemisphere generated by parcellation of the white surface using Desikan-Killiany parcellation
536	26886	Mean thickness of supramarginal in the right hemisphere generated by parcellation of the white surface using Desikan-Killiany parcellation
537	26887	Mean thickness of frontalpole in the right hemisphere generated by parcellation of the white surface using Desikan-Killiany parcellation
538	26888	Mean thickness of transversetemporal in the right hemisphere generated by parcellation of the white surface using Desikan-Killiany parcellation
539	26889	Mean thickness of insula in the right hemisphere generated by parcellation of the white surface using Desikan-Killiany parcellation
540	26890	Volume of bankssts in the right hemisphere generated by parcellation of the white surface using Desikan-Killiany parcellation
541	26891	Volume of caudalanteriorcingulate in the right hemisphere generated by parcellation of the white surface using Desikan-Killiany parcellation
542	26892	Volume of caudalmiddlefrontal in the right hemisphere generated by parcellation of the white surface using Desikan-Killiany parcellation
543	26893	Volume of cuneus in the right hemisphere generated by parcellation of the white surface using Desikan-Killiany parcellation
544	26894	Volume of entorhinal in the right hemisphere generated by parcellation of the white surface using Desikan-Killiany parcellation
545	26895	Volume of fusiform in the right hemisphere generated by parcellation of the white surface using Desikan-Killiany parcellation
546	26896	Volume of inferiorparietal in the right hemisphere generated by parcellation of the white surface using Desikan-Killiany parcellation
547	26897	Volume of inferiortemporal in the right hemisphere generated by parcellation of the white surface using Desikan-Killiany parcellation
548	26898	Volume of isthmuscingulate in the right hemisphere generated by parcellation of the white surface using Desikan-Killiany parcellation
549	26899	Volume of lateraloccipital in the right hemisphere generated by parcellation of the white surface using Desikan-Killiany parcellation
550	26900	Volume of lateralorbitofrontal in the right hemisphere generated by parcellation of the white surface using Desikan-Killiany parcellation
551	26901	Volume of lingual in the right hemisphere generated by parcellation of the white surface using Desikan-Killiany parcellation
552	26902	Volume of medialorbitofrontal in the right hemisphere generated by parcellation of the white surface using Desikan-Killiany parcellation
553	26903	Volume of middletemporal in the right hemisphere generated by parcellation of the white surface using Desikan-Killiany parcellation
554	26904	Volume of parahippocampal in the right hemisphere generated by parcellation of the white surface using Desikan-Killiany parcellation
555	26905	Volume of paracentral in the right hemisphere generated by parcellation of the white surface using Desikan-Killiany parcellation
556	26906	Volume of parsopercularis in the right hemisphere generated by parcellation of the white surface using Desikan-Killiany parcellation
557	26907	Volume of parsorbitalis in the right hemisphere generated by parcellation of the white surface using Desikan-Killiany parcellation
558	26908	Volume of parstriangularis in the right hemisphere generated by parcellation of the white surface using Desikan-Killiany parcellation
559	26909	Volume of pericalcarine in the right hemisphere generated by parcellation of the white surface using Desikan-Killiany parcellation
560	26910	Volume of postcentral in the right hemisphere generated by parcellation of the white surface using Desikan-Killiany parcellation
561	26911	Volume of posteriorcingulate in the right hemisphere generated by parcellation of the white surface using Desikan-Killiany parcellation
562	26912	Volume of precentral in the right hemisphere generated by parcellation of the white surface using Desikan-Killiany parcellation
563	26913	Volume of precuneus in the right hemisphere generated by parcellation of the white surface using Desikan-Killiany parcellation
564	26914	Volume of rostralanteriorcingulate in the right hemisphere generated by parcellation of the white surface using Desikan-Killiany parcellation
565	26915	Volume of rostralmiddlefrontal in the right hemisphere generated by parcellation of the white surface using Desikan-Killiany parcellation
566	26916	Volume of superiorfrontal in the right hemisphere generated by parcellation of the white surface using Desikan-Killiany parcellation
567	26917	Volume of superiorparietal in the right hemisphere generated by parcellation of the white surface using Desikan-Killiany parcellation
568	26918	Volume of superiortemporal in the right hemisphere generated by parcellation of the white surface using Desikan-Killiany parcellation
569	26919	Volume of supramarginal in the right hemisphere generated by parcellation of the white surface using Desikan-Killiany parcellation
570	26920	Volume of frontalpole in the right hemisphere generated by parcellation of the white surface using Desikan-Killiany parcellation
571	26921	Volume of transversetemporal in the right hemisphere generated by parcellation of the white surface using Desikan-Killiany parcellation
572	26922	Volume of insula in the right hemisphere generated by parcellation of the white surface using Desikan-Killiany parcellation
573	26923	Area of TotalSurface in the left hemisphere generated by parcellation of the pial surface using Desikan-Killiany parcellation
574	26924	Area of bankssts in the left hemisphere generated by parcellation of the pial surface using Desikan-Killiany parcellation
575	26925	Area of caudalanteriorcingulate in the left hemisphere generated by parcellation of the pial surface using Desikan-Killiany parcellation
576	26926	Area of caudalmiddlefrontal in the left hemisphere generated by parcellation of the pial surface using Desikan-Killiany parcellation
577	26927	Area of cuneus in the left hemisphere generated by parcellation of the pial surface using Desikan-Killiany parcellation
578	26928	Area of entorhinal in the left hemisphere generated by parcellation of the pial surface using Desikan-Killiany parcellation
579	26929	Area of fusiform in the left hemisphere generated by parcellation of the pial surface using Desikan-Killiany parcellation
580	26930	Area of inferiorparietal in the left hemisphere generated by parcellation of the pial surface using Desikan-Killiany parcellation
581	26931	Area of inferiortemporal in the left hemisphere generated by parcellation of the pial surface using Desikan-Killiany parcellation
582	26932	Area of isthmuscingulate in the left hemisphere generated by parcellation of the pial surface using Desikan-Killiany parcellation
583	26933	Area of lateraloccipital in the left hemisphere generated by parcellation of the pial surface using Desikan-Killiany parcellation
584	26934	Area of lateralorbitofrontal in the left hemisphere generated by parcellation of the pial surface using Desikan-Killiany parcellation
585	26935	Area of lingual in the left hemisphere generated by parcellation of the pial surface using Desikan-Killiany parcellation
586	26936	Area of medialorbitofrontal in the left hemisphere generated by parcellation of the pial surface using Desikan-Killiany parcellation
587	26937	Area of middletemporal in the left hemisphere generated by parcellation of the pial surface using Desikan-Killiany parcellation
588	26938	Area of parahippocampal in the left hemisphere generated by parcellation of the pial surface using Desikan-Killiany parcellation
589	26939	Area of paracentral in the left hemisphere generated by parcellation of the pial surface using Desikan-Killiany parcellation
590	26940	Area of parsopercularis in the left hemisphere generated by parcellation of the pial surface using Desikan-Killiany parcellation
591	26941	Area of parsorbitalis in the left hemisphere generated by parcellation of the pial surface using Desikan-Killiany parcellation
592	26942	Area of parstriangularis in the left hemisphere generated by parcellation of the pial surface using Desikan-Killiany parcellation
593	26943	Area of pericalcarine in the left hemisphere generated by parcellation of the pial surface using Desikan-Killiany parcellation
594	26944	Area of postcentral in the left hemisphere generated by parcellation of the pial surface using Desikan-Killiany parcellation
595	26945	Area of posteriorcingulate in the left hemisphere generated by parcellation of the pial surface using Desikan-Killiany parcellation
596	26946	Area of precentral in the left hemisphere generated by parcellation of the pial surface using Desikan-Killiany parcellation
597	26947	Area of precuneus in the left hemisphere generated by parcellation of the pial surface using Desikan-Killiany parcellation
598	26948	Area of rostralanteriorcingulate in the left hemisphere generated by parcellation of the pial surface using Desikan-Killiany parcellation
599	26949	Area of rostralmiddlefrontal in the left hemisphere generated by parcellation of the pial surface using Desikan-Killiany parcellation
600	26950	Area of superiorfrontal in the left hemisphere generated by parcellation of the pial surface using Desikan-Killiany parcellation
601	26951	Area of superiorparietal in the left hemisphere generated by parcellation of the pial surface using Desikan-Killiany parcellation
602	26952	Area of superiortemporal in the left hemisphere generated by parcellation of the pial surface using Desikan-Killiany parcellation
603	26953	Area of supramarginal in the left hemisphere generated by parcellation of the pial surface using Desikan-Killiany parcellation
604	26954	Area of frontalpole in the left hemisphere generated by parcellation of the pial surface using Desikan-Killiany parcellation
605	26955	Area of transversetemporal in the left hemisphere generated by parcellation of the pial surface using Desikan-Killiany parcellation
606	26956	Area of TotalSurface in the right hemisphere generated by parcellation of the pial surface using Desikan-Killiany parcellation
607	26957	Area of bankssts in the right hemisphere generated by parcellation of the pial surface using Desikan-Killiany parcellation
608	26958	Area of caudalanteriorcingulate in the right hemisphere generated by parcellation of the pial surface using Desikan-Killiany parcellation
609	26959	Area of caudalmiddlefrontal in the right hemisphere generated by parcellation of the pial surface using Desikan-Killiany parcellation
610	26960	Area of cuneus in the right hemisphere generated by parcellation of the pial surface using Desikan-Killiany parcellation
611	26961	Area of entorhinal in the right hemisphere generated by parcellation of the pial surface using Desikan-Killiany parcellation
612	26962	Area of fusiform in the right hemisphere generated by parcellation of the pial surface using Desikan-Killiany parcellation
613	26963	Area of inferiorparietal in the right hemisphere generated by parcellation of the pial surface using Desikan-Killiany parcellation
614	26964	Area of inferiortemporal in the right hemisphere generated by parcellation of the pial surface using Desikan-Killiany parcellation
615	26965	Area of isthmuscingulate in the right hemisphere generated by parcellation of the pial surface using Desikan-Killiany parcellation
616	26966	Area of lateraloccipital in the right hemisphere generated by parcellation of the pial surface using Desikan-Killiany parcellation
617	26967	Area of lateralorbitofrontal in the right hemisphere generated by parcellation of the pial surface using Desikan-Killiany parcellation
618	26968	Area of lingual in the right hemisphere generated by parcellation of the pial surface using Desikan-Killiany parcellation

619	26969	Area of medialorbitofrontal in the right hemisphere generated by parcellation of the pial surface using Desikan-Killiany parcellation
620	26970	Area of middletemporal in the right hemisphere generated by parcellation of the pial surface using Desikan-Killiany parcellation
621	26971	Area of parahippocampal in the right hemisphere generated by parcellation of the pial surface using Desikan-Killiany parcellation
622	26972	Area of paracentral in the right hemisphere generated by parcellation of the pial surface using Desikan-Killiany parcellation
623	26973	Area of parsopercularis in the right hemisphere generated by parcellation of the pial surface using Desikan-Killiany parcellation
624	26974	Area of parsorbitalis in the right hemisphere generated by parcellation of the pial surface using Desikan-Killiany parcellation
625	26975	Area of parstriangularis in the right hemisphere generated by parcellation of the pial surface using Desikan-Killiany parcellation
626	26976	Area of pericalcarine in the right hemisphere generated by parcellation of the pial surface using Desikan-Killiany parcellation
627	26977	Area of postcentral in the right hemisphere generated by parcellation of the pial surface using Desikan-Killiany parcellation
628	26978	Area of posteriorcingulate in the right hemisphere generated by parcellation of the pial surface using Desikan-Killiany parcellation
629	26979	Area of precentral in the right hemisphere generated by parcellation of the pial surface using Desikan-Killiany parcellation
630	26980	Area of precuneus in the right hemisphere generated by parcellation of the pial surface using Desikan-Killiany parcellation
631	26981	Area of rostralanteriorcingulate in the right hemisphere generated by parcellation of the pial surface using Desikan-Killiany parcellation
632	26982	Area of rostralmiddlefrontal in the right hemisphere generated by parcellation of the pial surface using Desikan-Killiany parcellation
633	26983	Area of superiorfrontal in the right hemisphere generated by parcellation of the pial surface using Desikan-Killiany parcellation
634	26984	Area of superiorparietal in the right hemisphere generated by parcellation of the pial surface using Desikan-Killiany parcellation
635	26985	Area of superiortemporal in the right hemisphere generated by parcellation of the pial surface using Desikan-Killiany parcellation
636	26986	Area of supramarginal in the right hemisphere generated by parcellation of the pial surface using Desikan-Killiany parcellation
637	26987	Area of frontalpole in the right hemisphere generated by parcellation of the pial surface using Desikan-Killiany parcellation
638	26988	Area of transversetemporal in the right hemisphere generated by parcellation of the pial surface using Desikan-Killiany parcellation
639	26989	Grey-white contrast in unknown in the left hemisphere calculated as a percentage of the mean gray-white matter intensity from Desikan-Killiany parcellation
640	26990	Grey-white contrast in bankssts in the left hemisphere calculated as a percentage of the mean gray-white matter intensity from Desikan-Killiany parcellation
641	26991	Grey-white contrast in caudalanteriorcingulate in the left hemisphere calculated as a percentage of the mean gray-white matter intensity from Desikan-Killiany parcellation
642	26992	Grey-white contrast in caudalmiddlefrontal in the left hemisphere calculated as a percentage of the mean gray-white matter intensity from Desikan-Killiany parcellation
643	26993	Grey-white contrast in cuneus in the left hemisphere calculated as a percentage of the mean gray-white matter intensity from Desikan-Killiany parcellation
644	26994	Grey-white contrast in entorhinal in the left hemisphere calculated as a percentage of the mean gray-white matter intensity from Desikan-Killiany parcellation
645	26995	Grey-white contrast in fusiform in the left hemisphere calculated as a percentage of the mean gray-white matter intensity from Desikan-Killiany parcellation
646	26996	Grey-white contrast in inferiorparietal in the left hemisphere calculated as a percentage of the mean gray-white matter intensity from Desikan-Killiany parcellation
647	26997	Grey-white contrast in inferiortemporal in the left hemisphere calculated as a percentage of the mean gray-white matter intensity from Desikan-Killiany parcellation
648	26998	Grey-white contrast in isthmuscingulate in the left hemisphere calculated as a percentage of the mean gray-white matter intensity from Desikan-Killiany parcellation
649	26999	Grey-white contrast in lateraloccipital in the left hemisphere calculated as a percentage of the mean gray-white matter intensity from Desikan-Killiany parcellation
650	27000	Grey-white contrast in lateralorbitofrontal in the left hemisphere calculated as a percentage of the mean gray-white matter intensity from Desikan-Killiany parcellation
651	27001	Grey-white contrast in lingual in the left hemisphere calculated as a percentage of the mean gray-white matter intensity from Desikan-Killiany parcellation
652	27002	Grey-white contrast in medialorbitofrontal in the left hemisphere calculated as a percentage of the mean gray-white matter intensity from Desikan-Killiany parcellation
653	27003	Grey-white contrast in middletemporal in the left hemisphere calculated as a percentage of the mean gray-white matter intensity from Desikan-Killiany parcellation
654	27004	Grey-white contrast in parahippocampal in the left hemisphere calculated as a percentage of the mean gray-white matter intensity from Desikan-Killiany parcellation
655	27005	Grey-white contrast in paracentral in the left hemisphere calculated as a percentage of the mean gray-white matter intensity from Desikan-Killiany parcellation
656	27006	Grey-white contrast in parsopercularis in the left hemisphere calculated as a percentage of the mean gray-white matter intensity from Desikan-Killiany parcellation
657	27007	Grey-white contrast in parsorbitalis in the left hemisphere calculated as a percentage of the mean gray-white matter intensity from Desikan-Killiany parcellation
658	27008	Grey-white contrast in parstriangularis in the left hemisphere calculated as a percentage of the mean gray-white matter intensity from Desikan-Killiany parcellation
659	27009	Grey-white contrast in pericalcarine in the left hemisphere calculated as a percentage of the mean gray-white matter intensity from Desikan-Killiany parcellation
660	27010	Grey-white contrast in postcentral in the left hemisphere calculated as a percentage of the mean gray-white matter intensity from Desikan-Killiany parcellation
661	27011	Grey-white contrast in posteriorcingulate in the left hemisphere calculated as a percentage of the mean gray-white matter intensity from Desikan-Killiany parcellation
662	27012	Grey-white contrast in precentral in the left hemisphere calculated as a percentage of the mean gray-white matter intensity from Desikan-Killiany parcellation
663	27013	Grey-white contrast in precuneus in the left hemisphere calculated as a percentage of the mean gray-white matter intensity from Desikan-Killiany parcellation
664	27014	Grey-white contrast in rostralanteriorcingulate in the left hemisphere calculated as a percentage of the mean gray-white matter intensity from Desikan-Killiany parcellation
665	27015	Grey-white contrast in rostralmiddlefrontal in the left hemisphere calculated as a percentage of the mean gray-white matter intensity from Desikan-Killiany parcellation
666	27016	Grey-white contrast in superiorfrontal in the left hemisphere calculated as a percentage of the mean gray-white matter intensity from Desikan-Killiany parcellation
667	27017	Grey-white contrast in superiorparietal in the left hemisphere calculated as a percentage of the mean gray-white matter intensity from Desikan-Killiany parcellation
668	27018	Grey-white contrast in superiortemporal in the left hemisphere calculated as a percentage of the mean gray-white matter intensity from Desikan-Killiany parcellation
669	27019	Grey-white contrast in supramarginal in the left hemisphere calculated as a percentage of the mean gray-white matter intensity from Desikan-Killiany parcellation
670	27020	Grey-white contrast in frontalpole in the left hemisphere calculated as a percentage of the mean gray-white matter intensity from Desikan-Killiany parcellation
671	27021	Grey-white contrast in temporalpole in the left hemisphere calculated as a percentage of the mean gray-white matter intensity from Desikan-Killiany parcellation
672	27022	Grey-white contrast in transversetemporal in the left hemisphere calculated as a percentage of the mean gray-white matter intensity from Desikan-Killiany parcellation
673	27023	Grey-white contrast in insula in the left hemisphere calculated as a percentage of the mean gray-white matter intensity from Desikan-Killiany parcellation
674	27024	Grey-white contrast in unknown in the right hemisphere calculated as a percentage of the mean gray-white matter intensity from Desikan-Killiany parcellation
675	27025	Grey-white contrast in bankssts in the right hemisphere calculated as a percentage of the mean gray-white matter intensity from Desikan-Killiany parcellation

676	27026	Grey-white contrast in caudalanteriorcingulate in the right hemisphere calculated as a percentage of the mean gray-white matter intensity from Desikan-Killiany parcellation
677	27027	Grey-white contrast in caudalmiddlefrontal in the right hemisphere calculated as a percentage of the mean gray-white matter intensity from Desikan-Killiany parcellation
678	27028	Grey-white contrast in cuneus in the right hemisphere calculated as a percentage of the mean gray-white matter intensity from Desikan-Killiany parcellation
679	27029	Grey-white contrast in entorhinal in the right hemisphere calculated as a percentage of the mean gray-white matter intensity from Desikan-Killiany parcellation
680	27030	Grey-white contrast in fusiform in the right hemisphere calculated as a percentage of the mean gray-white matter intensity from Desikan-Killiany parcellation
681	27031	Grey-white contrast in inferiorparietal in the right hemisphere calculated as a percentage of the mean gray-white matter intensity from Desikan-Killiany parcellation
682	27032	Grey-white contrast in inferiortemporal in the right hemisphere calculated as a percentage of the mean gray-white matter intensity from Desikan-Killiany parcellation
683	27033	Grey-white contrast in isthmuscingulate in the right hemisphere calculated as a percentage of the mean gray-white matter intensity from Desikan-Killiany parcellation
684	27034	Grey-white contrast in lateraloccipital in the right hemisphere calculated as a percentage of the mean gray-white matter intensity from Desikan-Killiany parcellation
685	27035	Grey-white contrast in lateralorbitofrontal in the right hemisphere calculated as a percentage of the mean gray-white matter intensity from Desikan-Killiany parcellation
686	27036	Grey-white contrast in lingual in the right hemisphere calculated as a percentage of the mean gray-white matter intensity from Desikan-Killiany parcellation
687	27037	Grey-white contrast in medialorbitofrontal in the right hemisphere calculated as a percentage of the mean gray-white matter intensity from Desikan-Killiany parcellation
688	27038	Grey-white contrast in middletemporal in the right hemisphere calculated as a percentage of the mean gray-white matter intensity from Desikan-Killiany parcellation
689	27039	Grey-white contrast in parahippocampal in the right hemisphere calculated as a percentage of the mean gray-white matter intensity from Desikan-Killiany parcellation
690	27040	Grey-white contrast in paracentral in the right hemisphere calculated as a percentage of the mean gray-white matter intensity from Desikan-Killiany parcellation
691	27041	Grey-white contrast in parsopercularis in the right hemisphere calculated as a percentage of the mean gray-white matter intensity from Desikan-Killiany parcellation
692	27042	Grey-white contrast in parsorbitalis in the right hemisphere calculated as a percentage of the mean gray-white matter intensity from Desikan-Killiany parcellation
693	27043	Grey-white contrast in parstriangularis in the right hemisphere calculated as a percentage of the mean gray-white matter intensity from Desikan-Killiany parcellation
694	27044	Grey-white contrast in pericalcarine in the right hemisphere calculated as a percentage of the mean gray-white matter intensity from Desikan-Killiany parcellation
695	27045	Grey-white contrast in postcentral in the right hemisphere calculated as a percentage of the mean gray-white matter intensity from Desikan-Killiany parcellation
696	27046	Grey-white contrast in posteriorcingulate in the right hemisphere calculated as a percentage of the mean gray-white matter intensity from Desikan-Killiany parcellation
697	27047	Grey-white contrast in precentral in the right hemisphere calculated as a percentage of the mean gray-white matter intensity from Desikan-Killiany parcellation
698	27048	Grey-white contrast in precuneus in the right hemisphere calculated as a percentage of the mean gray-white matter intensity from Desikan-Killiany parcellation
699	27049	Grey-white contrast in rostralanteriorcingulate in the right hemisphere calculated as a percentage of the mean gray-white matter intensity from Desikan-Killiany parcellation
700	27050	Grey-white contrast in rostralmiddlefrontal in the right hemisphere calculated as a percentage of the mean gray-white matter intensity from Desikan-Killiany parcellation
701	27051	Grey-white contrast in superiorfrontal in the right hemisphere calculated as a percentage of the mean gray-white matter intensity from Desikan-Killiany parcellation
702	27052	Grey-white contrast in superiorparietal in the right hemisphere calculated as a percentage of the mean gray-white matter intensity from Desikan-Killiany parcellation
703	27053	Grey-white contrast in superiortemporal in the right hemisphere calculated as a percentage of the mean gray-white matter intensity from Desikan-Killiany parcellation
704	27054	Grey-white contrast in supramarginal in the right hemisphere calculated as a percentage of the mean gray-white matter intensity from Desikan-Killiany parcellation
705	27055	Grey-white contrast in frontalpole in the right hemisphere calculated as a percentage of the mean gray-white matter intensity from Desikan-Killiany parcellation
706	27056	Grey-white contrast in temporalpole in the right hemisphere calculated as a percentage of the mean gray-white matter intensity from Desikan-Killiany parcellation
707	27057	Grey-white contrast in transversetemporal in the right hemisphere calculated as a percentage of the mean gray-white matter intensity from Desikan-Killiany parcellation
708	27058	Grey-white contrast in insula in the right hemisphere calculated as a percentage of the mean gray-white matter intensity from Desikan-Killiany parcellation
709	27059	Area of BA1 in the left hemisphere generated by parcellation of the white surface using BA_exvivo parcellation
710	27060	Area of BA2 in the left hemisphere generated by parcellation of the white surface using BA_exvivo parcellation
711	27061	Area of BA3a in the left hemisphere generated by parcellation of the white surface using BA_exvivo parcellation
712	27062	Area of BA3b in the left hemisphere generated by parcellation of the white surface using BA_exvivo parcellation
713	27063	Area of BA4a in the left hemisphere generated by parcellation of the white surface using BA_exvivo parcellation
714	27064	Area of BA4p in the left hemisphere generated by parcellation of the white surface using BA_exvivo parcellation
715	27065	Area of BA6 in the left hemisphere generated by parcellation of the white surface using BA_exvivo parcellation
716	27066	Area of BA44 in the left hemisphere generated by parcellation of the white surface using BA_exvivo parcellation
717	27067	Area of BA45 in the left hemisphere generated by parcellation of the white surface using BA_exvivo parcellation
718	27068	Area of V1 in the left hemisphere generated by parcellation of the white surface using BA_exvivo parcellation
719	27069	Area of V2 in the left hemisphere generated by parcellation of the white surface using BA_exvivo parcellation
720	27070	Area of MT in the left hemisphere generated by parcellation of the white surface using BA_exvivo parcellation
721	27071	Area of perirhinal in the left hemisphere generated by parcellation of the white surface using BA_exvivo parcellation
722	27072	Area of entorhinal in the left hemisphere generated by parcellation of the white surface using BA_exvivo parcellation
723	27073	Mean thickness of BA1 in the left hemisphere generated by parcellation of the white surface using BA_exvivo parcellation
724	27074	Mean thickness of BA2 in the left hemisphere generated by parcellation of the white surface using BA_exvivo parcellation
725	27075	Mean thickness of BA3a in the left hemisphere generated by parcellation of the white surface using BA_exvivo parcellation
726	27076	BMean thickness of BA3b in the left hemisphere generated by parcellation of the white surface using BA_exvivo parcellation
727	27077	Mean thickness of BA4a in the left hemisphere generated by parcellation of the white surface using BA_exvivo parcellation
728	27078	Mean thickness of BA4p in the left hemisphere generated by parcellation of the white surface using BA_exvivo parcellation
729	27079	Mean thickness of BA6 in the left hemisphere generated by parcellation of the white surface using BA_exvivo parcellation
730	27080	Mean thickness of BA44 in the left hemisphere generated by parcellation of the white surface using BA_exvivo parcellation
731	27081	Mean thickness of BA45 in the left hemisphere generated by parcellation of the white surface using BA_exvivo parcellation
732	27082	Mean thickness of V1 in the left hemisphere generated by parcellation of the white surface using BA_exvivo parcellation
733	27083	Mean thickness of V2 in the left hemisphere generated by parcellation of the white surface using BA_exvivo parcellation
734	27084	Mean thickness of MT in the left hemisphere generated by parcellation of the white surface using BA_exvivo parcellation

735	27085	Mean thickness of perirhinal in the left hemisphere generated by parcellation of the white surface using BA_exvivo parcellation
736	27086	Mean thickness of entorhinal in the left hemisphere generated by parcellation of the white surface using BA_exvivo parcellation
737	27087	Volume of BA1 in the left hemisphere generated by parcellation of the white surface using BA_exvivo parcellation
738	27088	Volume of BA2 in the left hemisphere generated by parcellation of the white surface using BA_exvivo parcellation
739	27089	Volume of BA3a in the left hemisphere generated by parcellation of the white surface using BA_exvivo parcellation
740	27090	Volume of BA3b in the left hemisphere generated by parcellation of the white surface using BA_exvivo parcellation
741	27091	Volume of BA4a in the left hemisphere generated by parcellation of the white surface using BA_exvivo parcellation
742	27092	Volume of BA4p in the left hemisphere generated by parcellation of the white surface using BA_exvivo parcellation
743	27093	Volume of BA6 in the left hemisphere generated by parcellation of the white surface using BA_exvivo parcellation
744	27094	Volume of BA44 in the left hemisphere generated by parcellation of the white surface using BA_exvivo parcellation
745	27095	Volume of BA45 in the left hemisphere generated by parcellation of the white surface using BA_exvivo parcellation
746	27096	Volume of V1 in the left hemisphere generated by parcellation of the white surface using BA_exvivo parcellation
747	27097	Volume of V2 in the left hemisphere generated by parcellation of the white surface using BA_exvivo parcellation
748	27098	Volume of MT in the left hemisphere generated by parcellation of the white surface using BA_exvivo parcellation
749	27099	Volume of perirhinal in the left hemisphere generated by parcellation of the white surface using BA_exvivo parcellation
750	27100	Volume of entorhinal in the left hemisphere generated by parcellation of the white surface using BA_exvivo parcellation
751	27101	Area of BA1 in the right hemisphere generated by parcellation of the white surface using BA_exvivo parcellation
752	27102	Area of BA2 in the right hemisphere generated by parcellation of the white surface using BA_exvivo parcellation
753	27103	Area of BA3a in the right hemisphere generated by parcellation of the white surface using BA_exvivo parcellation
754	27104	Area of BA3b in the right hemisphere generated by parcellation of the white surface using BA_exvivo parcellation
755	27105	Area of BA4a in the right hemisphere generated by parcellation of the white surface using BA_exvivo parcellation
756	27106	Area of BA4p in the right hemisphere generated by parcellation of the white surface using BA_exvivo parcellation
757	27107	Area of BA6 in the right hemisphere generated by parcellation of the white surface using BA_exvivo parcellation
758	27108	Area of BA44 in the right hemisphere generated by parcellation of the white surface using BA_exvivo parcellation
759	27109	Area of BA45 in the right hemisphere generated by parcellation of the white surface using BA_exvivo parcellation
760	27110	Area of V1 in the right hemisphere generated by parcellation of the white surface using BA_exvivo parcellation
761	27111	Area of V2 in the right hemisphere generated by parcellation of the white surface using BA_exvivo parcellation
762	27112	Area of MT in the right hemisphere generated by parcellation of the white surface using BA_exvivo parcellation
763	27113	Area of perirhinal in the right hemisphere generated by parcellation of the white surface using BA_exvivo parcellation
764	27114	Area of entorhinal in the right hemisphere generated by parcellation of the white surface using BA_exvivo parcellation
765	27115	Mean thickness of BA1 in the right hemisphere generated by parcellation of the white surface using BA_exvivo parcellation
766	27116	Mean thickness of BA2 in the right hemisphere generated by parcellation of the white surface using BA_exvivo parcellation
767	27117	Mean thickness of BA3a in the right hemisphere generated by parcellation of the white surface using BA_exvivo parcellation
768	27118	Mean thickness of BA3b in the right hemisphere generated by parcellation of the white surface using BA_exvivo parcellation
769	27119	Mean thickness of BA4a in the right hemisphere generated by parcellation of the white surface using BA_exvivo parcellation
770	27120	Mean thickness of BA4p in the right hemisphere generated by parcellation of the white surface using BA_exvivo parcellation
771	27121	Mean thickness of BA6 in the right hemisphere generated by parcellation of the white surface using BA_exvivo parcellation
772	27122	Mean thickness of BA44 in the right hemisphere generated by parcellation of the white surface using BA_exvivo parcellation
773	27123	Mean thickness of BA45 in the right hemisphere generated by parcellation of the white surface using BA_exvivo parcellation
774	27124	Mean thickness of V1 in the right hemisphere generated by parcellation of the white surface using BA_exvivo parcellation
775	27125	Mean thickness of V2 in the right hemisphere generated by parcellation of the white surface using BA_exvivo parcellation
776	27126	Mean thickness of MT in the right hemisphere generated by parcellation of the white surface using BA_exvivo parcellation
777	27127	Mean thickness of perirhinal in the right hemisphere generated by parcellation of the white surface using BA_exvivo parcellation
778	27128	Mean thickness of entorhinal in the right hemisphere generated by parcellation of the white surface using BA_exvivo parcellation
779	27129	Volume of BA1 in the right hemisphere generated by parcellation of the white surface using BA_exvivo parcellation
780	27130	Volume of BA2 in the right hemisphere generated by parcellation of the white surface using BA_exvivo parcellation
781	27131	Volume of BA3a in the right hemisphere generated by parcellation of the white surface using BA_exvivo parcellation
782	27132	Volume of BA3b in the right hemisphere generated by parcellation of the white surface using BA_exvivo parcellation
783	27133	Volume of BA4a in the right hemisphere generated by parcellation of the white surface using BA_exvivo parcellation
784	27134	Volume of BA4p in the right hemisphere generated by parcellation of the white surface using BA_exvivo parcellation
785	27135	Volume of BA6 in the right hemisphere generated by parcellation of the white surface using BA_exvivo parcellation
786	27136	Volume of BA44 in the right hemisphere generated by parcellation of the white surface using BA_exvivo parcellation
787	27137	Volume of BA45 in the right hemisphere generated by parcellation of the white surface using BA_exvivo parcellation
788	27138	Volume of V1 in the right hemisphere generated by parcellation of the white surface using BA_exvivo parcellation
789	27139	Volume of V2 in the right hemisphere generated by parcellation of the white surface using BA_exvivo parcellation
790	27140	Volume of MT in the right hemisphere generated by parcellation of the white surface using BA_exvivo parcellation
791	27141	Volume of perirhinal in the right hemisphere generated by parcellation of the white surface using BA_exvivo parcellation
792	27142	Volume of entorhinal in the right hemisphere generated by parcellation of the white surface using BA_exvivo parcellation
793	27143	Area of caudalanteriorcingulate in the left hemisphere generated by parcellation of the white surface using DKT parcellation
794	27144	Area of caudalmiddlefrontal in the left hemisphere generated by parcellation of the white surface using DKT parcellation
795	27145	Area of cuneus in the left hemisphere generated by parcellation of the white surface using DKT parcellation
796	27146	Area of entorhinal in the left hemisphere generated by parcellation of the white surface using DKT parcellation
797	27147	Area of fusiform in the left hemisphere generated by parcellation of the white surface using DKT parcellation
798	27148	Area of inferiorparietal in the left hemisphere generated by parcellation of the white surface using DKT parcellation
799	27149	Area of inferiortemporal in the left hemisphere generated by parcellation of the white surface using DKT parcellation
800	27150	Area of isthmuscingulate in the left hemisphere generated by parcellation of the white surface using DKT parcellation
801	27151	Area of lateraloccipital in the left hemisphere generated by parcellation of the white surface using DKT parcellation
802	27152	Area of lateralorbitofrontal in the left hemisphere generated by parcellation of the white surface using DKT parcellation
803	27153	Area of lingual in the left hemisphere generated by parcellation of the white surface using DKT parcellation
804	27154	Area of medialorbitofrontal in the left hemisphere generated by parcellation of the white surface using DKT parcellation
805	27155	Area of middletemporal in the left hemisphere generated by parcellation of the white surface using DKT parcellation
806	27156	Area of parahippocampal in the left hemisphere generated by parcellation of the white surface using DKT parcellation
807	27157	Area of paracentral in the left hemisphere generated by parcellation of the white surface using DKT parcellation
808	27158	Area of parsopercularis in the left hemisphere generated by parcellation of the white surface using DKT parcellation
809	27159	Area of parsorbitalis in the left hemisphere generated by parcellation of the white surface using DKT parcellation
810	27160	Area of parstriangularis in the left hemisphere generated by parcellation of the white surface using DKT parcellation
811	27161	Area of pericalcarine in the left hemisphere generated by parcellation of the white surface using DKT parcellation
812	27162	Area of postcentral in the left hemisphere generated by parcellation of the white surface using DKT parcellation
813	27163	Area of posteriorcingulate in the left hemisphere generated by parcellation of the white surface using DKT parcellation
814	27164	Area of precentral in the left hemisphere generated by parcellation of the white surface using DKT parcellation
815	27165	Area of precuneus in the left hemisphere generated by parcellation of the white surface using DKT parcellation
816	27166	Area of rostralanteriorcingulate in the left hemisphere generated by parcellation of the white surface using DKT parcellation
817	27167	Area of rostralmiddlefrontal in the left hemisphere generated by parcellation of the white surface using DKT parcellation
818	27168	Area of superiorfrontal in the left hemisphere generated by parcellation of the white surface using DKT parcellation
819	27169	Area of superiorparietal in the left hemisphere generated by parcellation of the white surface using DKT parcellation
820	27170	Area of superiortemporal in the left hemisphere generated by parcellation of the white surface using DKT parcellation
821	27171	Area of supramarginal in the left hemisphere generated by parcellation of the white surface using DKT parcellation
822	27172	Area of transversetemporal in the left hemisphere generated by parcellation of the white surface using DKT parcellation
823	27173	Area of insula in the left hemisphere generated by parcellation of the white surface using DKT parcellation

824	27174	Mean thickness of caudalanteriorcingulate in the left hemisphere generated by parcellation of the white surface using DKT parcellation
825	27175	Mean thickness of caudalmiddlefrontal in the left hemisphere generated by parcellation of the white surface using DKT parcellation
826	27176	Mean thickness of cuneus in the left hemisphere generated by parcellation of the white surface using DKT parcellation
827	27177	Mean thickness of entorhinal in the left hemisphere generated by parcellation of the white surface using DKT parcellation
828	27178	Mean thickness of fusiform in the left hemisphere generated by parcellation of the white surface using DKT parcellation
829	27179	Mean thickness of inferiorparietal in the left hemisphere generated by parcellation of the white surface using DKT parcellation
830	27180	Mean thickness of inferiortemporal in the left hemisphere generated by parcellation of the white surface using DKT parcellation
831	27181	Mean thickness of isthmuscingulate in the left hemisphere generated by parcellation of the white surface using DKT parcellation
832	27182	Mean thickness of lateraloccipital in the left hemisphere generated by parcellation of the white surface using DKT parcellation
833	27183	Mean thickness of lateralorbitofrontal in the left hemisphere generated by parcellation of the white surface using DKT parcellation
834	27184	Mean thickness of lingual in the left hemisphere generated by parcellation of the white surface using DKT parcellation
835	27185	Mean thickness of medialorbitofrontal in the left hemisphere generated by parcellation of the white surface using DKT parcellation
836	27186	Mean thickness of middletemporal in the left hemisphere generated by parcellation of the white surface using DKT parcellation
837	27187	Mean thickness of parahippocampal in the left hemisphere generated by parcellation of the white surface using DKT parcellation
838	27188	Mean thickness of paracentral in the left hemisphere generated by parcellation of the white surface using DKT parcellation
839	27189	Mean thickness of parsopercularis in the left hemisphere generated by parcellation of the white surface using DKT parcellation
840	27190	Mean thickness of parsorbitalis in the left hemisphere generated by parcellation of the white surface using DKT parcellation
841	27191	Mean thickness of parstriangularis in the left hemisphere generated by parcellation of the white surface using DKT parcellation
842	27192	Mean thickness of pericalcarine in the left hemisphere generated by parcellation of the white surface using DKT parcellation
843	27193	Mean thickness of postcentral in the left hemisphere generated by parcellation of the white surface using DKT parcellation
844	27194	Mean thickness of posteriorcingulate in the left hemisphere generated by parcellation of the white surface using DKT parcellation
845	27195	Mean thickness of precentral in the left hemisphere generated by parcellation of the white surface using DKT parcellation
846	27196	Mean thickness of precuneus in the left hemisphere generated by parcellation of the white surface using DKT parcellation
847	27197	Mean thickness of rostralanteriorcingulate in the left hemisphere generated by parcellation of the white surface using DKT parcellation
848	27198	Mean thickness of rostralmiddlefrontal in the left hemisphere generated by parcellation of the white surface using DKT parcellation
849	27199	Mean thickness of superiorfrontal in the left hemisphere generated by parcellation of the white surface using DKT parcellation
850	27200	Mean thickness of superiorparietal in the left hemisphere generated by parcellation of the white surface using DKT parcellation
851	27201	Mean thickness of superiortemporal in the left hemisphere generated by parcellation of the white surface using DKT parcellation
852	27202	Mean thickness of supramarginal in the left hemisphere generated by parcellation of the white surface using DKT parcellation
853	27203	Mean thickness of transversetemporal in the left hemisphere generated by parcellation of the white surface using DKT parcellation
854	27204	Mean thickness of insula in the left hemisphere generated by parcellation of the white surface using DKT parcellation
855	27205	Volume of caudalanteriorcingulate in the left hemisphere generated by parcellation of the white surface using DKT parcellation
856	27206	Volume of caudalmiddlefrontal in the left hemisphere generated by parcellation of the white surface using DKT parcellation
857	27207	Volume of cuneus in the left hemisphere generated by parcellation of the white surface using DKT parcellation
858	27208	Volume of entorhinal in the left hemisphere generated by parcellation of the white surface using DKT parcellation
859	27209	Volume of fusiform in the left hemisphere generated by parcellation of the white surface using DKT parcellation
860	27210	Volume of inferiorparietal in the left hemisphere generated by parcellation of the white surface using DKT parcellation
861	27211	Volume of inferiortemporal in the left hemisphere generated by parcellation of the white surface using DKT parcellation
862	27212	Volume of isthmuscingulate in the left hemisphere generated by parcellation of the white surface using DKT parcellation
863	27213	Volume of lateraloccipital in the left hemisphere generated by parcellation of the white surface using DKT parcellation
864	27214	Volume of lateralorbitofrontal in the left hemisphere generated by parcellation of the white surface using DKT parcellation
865	27215	Volume of lingual in the left hemisphere generated by parcellation of the white surface using DKT parcellation
866	27216	Volume of medialorbitofrontal in the left hemisphere generated by parcellation of the white surface using DKT parcellation
867	27217	Volume of middletemporal in the left hemisphere generated by parcellation of the white surface using DKT parcellation
868	27218	Volume of parahippocampal in the left hemisphere generated by parcellation of the white surface using DKT parcellation
869	27219	Volume of paracentral in the left hemisphere generated by parcellation of the white surface using DKT parcellation
870	27220	Volume of parsopercularis in the left hemisphere generated by parcellation of the white surface using DKT parcellation
871	27221	Volume of parsorbitalis in the left hemisphere generated by parcellation of the white surface using DKT parcellation
872	27222	Volume of parstriangularis in the left hemisphere generated by parcellation of the white surface using DKT parcellation
873	27223	Volume of pericalcarine in the left hemisphere generated by parcellation of the white surface using DKT parcellation
874	27224	Volume of postcentral in the left hemisphere generated by parcellation of the white surface using DKT parcellation
875	27225	Volume of posteriorcingulate in the left hemisphere generated by parcellation of the white surface using DKT parcellation
876	27226	Volume of precentral in the left hemisphere generated by parcellation of the white surface using DKT parcellation
877	27227	Volume of precuneus in the left hemisphere generated by parcellation of the white surface using DKT parcellation
878	27228	Volume of rostralanteriorcingulate in the left hemisphere generated by parcellation of the white surface using DKT parcellation
879	27229	Volume of rostralmiddlefrontal in the left hemisphere generated by parcellation of the white surface using DKT parcellation
880	27230	Volume of superiorfrontal in the left hemisphere generated by parcellation of the white surface using DKT parcellation
881	27231	Volume of superiorparietal in the left hemisphere generated by parcellation of the white surface using DKT parcellation
882	27232	Volume of superiortemporal in the left hemisphere generated by parcellation of the white surface using DKT parcellation
883	27233	Volume of supramarginal in the left hemisphere generated by parcellation of the white surface using DKT parcellation
884	27234	Volume of transversetemporal in the left hemisphere generated by parcellation of the white surface using DKT parcellation
885	27235	Volume of insula in the left hemisphere generated by parcellation of the white surface using DKT parcellation
886	27236	Area of caudalanteriorcingulate in the right hemisphere generated by parcellation of the white surface using DKT parcellation
887	27237	Area of caudalmiddlefrontal in the right hemisphere generated by parcellation of the white surface using DKT parcellation
888	27238	Area of cuneus in the right hemisphere generated by parcellation of the white surface using DKT parcellation
889	27239	Area of entorhinal in the right hemisphere generated by parcellation of the white surface using DKT parcellation
890	27240	Area of fusiform in the right hemisphere generated by parcellation of the white surface using DKT parcellation
891	27241	Area of inferiorparietal in the right hemisphere generated by parcellation of the white surface using DKT parcellation
892	27242	Area of inferiortemporal in the right hemisphere generated by parcellation of the white surface using DKT parcellation
893	27243	Area of isthmuscingulate in the right hemisphere generated by parcellation of the white surface using DKT parcellation
894	27244	Area of lateraloccipital in the right hemisphere generated by parcellation of the white surface using DKT parcellation
895	27245	Area of lateralorbitofrontal in the right hemisphere generated by parcellation of the white surface using DKT parcellation
896	27246	Area of lingual in the right hemisphere generated by parcellation of the white surface using DKT parcellation
897	27247	Area of medialorbitofrontal in the right hemisphere generated by parcellation of the white surface using DKT parcellation
898	27248	Area of middletemporal in the right hemisphere generated by parcellation of the white surface using DKT parcellation
899	27249	Area of parahippocampal in the right hemisphere generated by parcellation of the white surface using DKT parcellation
900	27250	Area of paracentral in the right hemisphere generated by parcellation of the white surface using DKT parcellation
901	27251	Area of parsopercularis in the right hemisphere generated by parcellation of the white surface using DKT parcellation
902	27252	Area of parsorbitalis in the right hemisphere generated by parcellation of the white surface using DKT parcellation
903	27253	Area of parstriangularis in the right hemisphere generated by parcellation of the white surface using DKT parcellation
904	27254	Area of pericalcarine in the right hemisphere generated by parcellation of the white surface using DKT parcellation
905	27255	Area of postcentral in the right hemisphere generated by parcellation of the white surface using DKT parcellation
906	27256	Area of posteriorcingulate in the right hemisphere generated by parcellation of the white surface using DKT parcellation
907	27257	Area of precentral in the right hemisphere generated by parcellation of the white surface using DKT parcellation
908	27258	Area of precuneus in the right hemisphere generated by parcellation of the white surface using DKT parcellation
909	27259	Area of rostralanteriorcingulate in the right hemisphere generated by parcellation of the white surface using DKT parcellation
910	27260	Area of rostralmiddlefrontal in the right hemisphere generated by parcellation of the white surface using DKT parcellation
911	27261	Area of superiorfrontal in the right hemisphere generated by parcellation of the white surface using DKT parcellation
912	27262	Area of superiorparietal in the right hemisphere generated by parcellation of the white surface using DKT parcellation

913	27263	Area of superiortemporal in the right hemisphere generated by parcellation of the white surface using DKT parcellation
914	27264	Area of supramarginal in the right hemisphere generated by parcellation of the white surface using DKT parcellation
915	27265	Area of transversetemporal in the right hemisphere generated by parcellation of the white surface using DKT parcellation
916	27266	Area of insula in the right hemisphere generated by parcellation of the white surface using DKT parcellation
917	27267	Mean thickness of caudalanteriorcingulate in the right hemisphere generated by parcellation of the white surface using DKT parcellation
918	27268	Mean thickness of caudalmiddlefrontal in the right hemisphere generated by parcellation of the white surface using DKT parcellation
919	27269	Mean thickness of cuneus in the right hemisphere generated by parcellation of the white surface using DKT parcellation
920	27270	Mean thickness of entorhinal in the right hemisphere generated by parcellation of the white surface using DKT parcellation
921	27271	Mean thickness of fusiform in the right hemisphere generated by parcellation of the white surface using DKT parcellation
922	27272	Mean thickness of inferiorparietal in the right hemisphere generated by parcellation of the white surface using DKT parcellation
923	27273	Mean thickness of inferiortemporal in the right hemisphere generated by parcellation of the white surface using DKT parcellation
924	27274	Mean thickness of isthmuscingulate in the right hemisphere generated by parcellation of the white surface using DKT parcellation
925	27275	Mean thickness of lateraloccipital in the right hemisphere generated by parcellation of the white surface using DKT parcellation
926	27276	Mean thickness of lateralorbitofrontal in the right hemisphere generated by parcellation of the white surface using DKT parcellation
927	27277	Mean thickness of lingual in the right hemisphere generated by parcellation of the white surface using DKT parcellation
928	27278	Mean thickness of medialorbitofrontal in the right hemisphere generated by parcellation of the white surface using DKT parcellation
929	27279	Mean thickness of middletemporal in the right hemisphere generated by parcellation of the white surface using DKT parcellation
930	27280	Mean thickness of parahippocampal in the right hemisphere generated by parcellation of the white surface using DKT parcellation
931	27281	Mean thickness of paracentral in the right hemisphere generated by parcellation of the white surface using DKT parcellation
932	27282	Mean thickness of parsopercularis in the right hemisphere generated by parcellation of the white surface using DKT parcellation
933	27283	Mean thickness of parsorbitalis in the right hemisphere generated by parcellation of the white surface using DKT parcellation
934	27284	Mean thickness of parstriangularis in the right hemisphere generated by parcellation of the white surface using DKT parcellation
935	27285	Mean thickness of pericalcarine in the right hemisphere generated by parcellation of the white surface using DKT parcellation
936	27286	Mean thickness of postcentral in the right hemisphere generated by parcellation of the white surface using DKT parcellation
937	27287	Mean thickness of posteriorcingulate in the right hemisphere generated by parcellation of the white surface using DKT parcellation
938	27288	Mean thickness of precentral in the right hemisphere generated by parcellation of the white surface using DKT parcellation
939	27289	Mean thickness of precuneus in the right hemisphere generated by parcellation of the white surface using DKT parcellation
940	27290	Mean thickness of rostralanteriorcingulate in the right hemisphere generated by parcellation of the white surface using DKT parcellation
941	27291	Mean thickness of rostralmiddlefrontal in the right hemisphere generated by parcellation of the white surface using DKT parcellation
942	27292	Mean thickness of superiorfrontal in the right hemisphere generated by parcellation of the white surface using DKT parcellation
943	27293	Mean thickness of superiorparietal in the right hemisphere generated by parcellation of the white surface using DKT parcellation
944	27294	Mean thickness of superiortemporal in the right hemisphere generated by parcellation of the white surface using DKT parcellation
945	27295	Mean thickness of supramarginal in the right hemisphere generated by parcellation of the white surface using DKT parcellation
946	27296	Mean thickness of transversetemporal in the right hemisphere generated by parcellation of the white surface using DKT parcellation
947	27297	Mean thickness of insula in the right hemisphere generated by parcellation of the white surface using DKT parcellation
948	27298	Volume of caudalanteriorcingulate in the right hemisphere generated by parcellation of the white surface using DKT parcellation
949	27299	Volume of caudalmiddlefrontal in the right hemisphere generated by parcellation of the white surface using DKT parcellation
950	27300	Volume of cuneus in the right hemisphere generated by parcellation of the white surface using DKT parcellation
951	27301	Volume of entorhinal in the right hemisphere generated by parcellation of the white surface using DKT parcellation
952	27302	Volume of fusiform in the right hemisphere generated by parcellation of the white surface using DKT parcellation
953	27303	Volume of inferiorparietal in the right hemisphere generated by parcellation of the white surface using DKT parcellation
954	27304	Volume of inferiortemporal in the right hemisphere generated by parcellation of the white surface using DKT parcellation
955	27305	Volume of isthmuscingulate in the right hemisphere generated by parcellation of the white surface using DKT parcellation
956	27306	Volume of lateraloccipital in the right hemisphere generated by parcellation of the white surface using DKT parcellation
957	27307	Volume of lateralorbitofrontal in the right hemisphere generated by parcellation of the white surface using DKT parcellation
958	27308	Volume of lingual in the right hemisphere generated by parcellation of the white surface using DKT parcellation
959	27309	Volume of medialorbitofrontal in the right hemisphere generated by parcellation of the white surface using DKT parcellation
960	27310	Volume of middletemporal in the right hemisphere generated by parcellation of the white surface using DKT parcellation
961	27311	Volume of parahippocampal in the right hemisphere generated by parcellation of the white surface using DKT parcellation
962	27312	Volume of paracentral in the right hemisphere generated by parcellation of the white surface using DKT parcellation
963	27313	Volume of parsopercularis in the right hemisphere generated by parcellation of the white surface using DKT parcellation
964	27314	Volume of parsorbitalis in the right hemisphere generated by parcellation of the white surface using DKT parcellation
965	27315	Volume of parstriangularis in the right hemisphere generated by parcellation of the white surface using DKT parcellation
966	27316	Volume of pericalcarine in the right hemisphere generated by parcellation of the white surface using DKT parcellation
967	27317	Volume of postcentral in the right hemisphere generated by parcellation of the white surface using DKT parcellation
968	27318	Volume of posteriorcingulate in the right hemisphere generated by parcellation of the white surface using DKT parcellation
969	27319	Volume of precentral in the right hemisphere generated by parcellation of the white surface using DKT parcellation
970	27320	Volume of precuneus in the right hemisphere generated by parcellation of the white surface using DKT parcellation
971	27321	Volume of rostralanteriorcingulate in the right hemisphere generated by parcellation of the white surface using DKT parcellation
972	27322	Volume of rostralmiddlefrontal in the right hemisphere generated by parcellation of the white surface using DKT parcellation
973	27323	Volume of superiorfrontal in the right hemisphere generated by parcellation of the white surface using DKT parcellation
974	27324	Volume of superiorparietal in the right hemisphere generated by parcellation of the white surface using DKT parcellation
975	27325	Volume of superiortemporal in the right hemisphere generated by parcellation of the white surface using DKT parcellation
976	27326	Volume of supramarginal in the right hemisphere generated by parcellation of the white surface using DKT parcellation
977	27327	Volume of transversetemporal in the right hemisphere generated by parcellation of the white surface using DKT parcellation
978	27328	Volume of insula in the right hemisphere generated by parcellation of the white surface using DKT parcellation
979	27329	Area of G+S-frontomargin in the left hemisphere generated by parcellation of the white surface using Destrieux (a2009s) parcellation
980	27330	Area of G+S-occipital-inf in the left hemisphere generated by parcellation of the white surface using Destrieux (a2009s) parcellation
981	27331	Area of G+S-paracentral in the left hemisphere generated by parcellation of the white surface using Destrieux (a2009s) parcellation
982	27332	Area of G+S-subcentral in the left hemisphere generated by parcellation of the white surface using Destrieux (a2009s) parcellation
983	27333	Area of G+S-transv-frontopol in the left hemisphere generated by parcellation of the white surface using Destrieux (a2009s) parcellation
984	27334	Area of G+S-cingul-Ant in the left hemisphere generated by parcellation of the white surface using Destrieux (a2009s) parcellation
985	27335	Area of G+S-cingul-Mid-Ant in the left hemisphere generated by parcellation of the white surface using Destrieux (a2009s) parcellation
986	27336	Area of G+S-cingul-Mid-Post in the left hemisphere generated by parcellation of the white surface using Destrieux (a2009s) parcellation
987	27337	Area of G-cingul-Post-dorsal in the left hemisphere generated by parcellation of the white surface using Destrieux (a2009s) parcellation
988	27338	Area of G-cingul-Post-ventral in the left hemisphere generated by parcellation of the white surface using Destrieux (a2009s) parcellation
989	27339	Area of G-cuneus in the left hemisphere generated by parcellation of the white surface using Destrieux (a2009s) parcellation
990	27340	Area of G-front-inf-Opercular in the left hemisphere generated by parcellation of the white surface using Destrieux (a2009s) parcellation
991	27341	Area of G-front-inf-Orbital in the left hemisphere generated by parcellation of the white surface using Destrieux (a2009s) parcellation
992	27342	Area of G-front-inf-Triangul in the left hemisphere generated by parcellation of the white surface using Destrieux (a2009s) parcellation
993	27343	Area of G-front-middle in the left hemisphere generated by parcellation of the white surface using Destrieux (a2009s) parcellation
994	27344	Area of G-front-sup in the left hemisphere generated by parcellation of the white surface using Destrieux (a2009s) parcellation
995	27345	Area of G-Ins-lg+S-cent-ins in the left hemisphere generated by parcellation of the white surface using Destrieux (a2009s) parcellation
996	27346	Area of G-insular-short in the left hemisphere generated by parcellation of the white surface using Destrieux (a2009s) parcellation
997	27347	Area of G-occipital-middle in the left hemisphere generated by parcellation of the white surface using Destrieux (a2009s) parcellation
998	27348	Area of G-occipital-sup in the left hemisphere generated by parcellation of the white surface using Destrieux (a2009s) parcellation
999	27349	Area of G-oc-temp-lat-fusifor in the left hemisphere generated by parcellation of the white surface using Destrieux (a2009s) parcellation
1000	27350	Area of G-oc-temp-med-Lingual in the left hemisphere generated by parcellation of the white surface using Destrieux (a2009s) parcellation
1001	27351	Area of G-oc-temp-med-Parahip in the left hemisphere generated by parcellation of the white surface using Destrieux (a2009s) parcellation

1002	27352	Area of G-orbital in the left hemisphere generated by parcellation of the white surface using Destrieux (a2009s) parcellation
1003	27353	Area of G-pariet-inf-Angular in the left hemisphere generated by parcellation of the white surface using Destrieux (a2009s) parcellation
1004	27354	Area of G-pariet-inf-Supramar in the left hemisphere generated by parcellation of the white surface using Destrieux (a2009s) parcellation
1005	27355	Area of G-parietal-sup in the left hemisphere generated by parcellation of the white surface using Destrieux (a2009s) parcellation
1006	27356	Area of G-postcentral in the left hemisphere generated by parcellation of the white surface using Destrieux (a2009s) parcellation
1007	27357	Area of G-precentral in the left hemisphere generated by parcellation of the white surface using Destrieux (a2009s) parcellation
1008	27358	Area of G-precuneus in the left hemisphere generated by parcellation of the white surface using Destrieux (a2009s) parcellation
1009	27359	Area of G-rectus in the left hemisphere generated by parcellation of the white surface using Destrieux (a2009s) parcellation
1010	27360	Area of G-subcallosal in the left hemisphere generated by parcellation of the white surface using Destrieux (a2009s) parcellation
1011	27361	Area of G-temp-sup-G-T-transv in the left hemisphere generated by parcellation of the white surface using Destrieux (a2009s) parcellation
1012	27362	Area of G-temp-sup-Lateral in the left hemisphere generated by parcellation of the white surface using Destrieux (a2009s) parcellation
1013	27363	Area of G-temp-sup-Plan-polar in the left hemisphere generated by parcellation of the white surface using Destrieux (a2009s) parcellation
1014	27364	Area of G-temp-sup-Plan-tempo in the left hemisphere generated by parcellation of the white surface using Destrieux (a2009s) parcellation
1015	27365	Area of G-temporal-inf in the left hemisphere generated by parcellation of the white surface using Destrieux (a2009s) parcellation
1016	27366	Area of G-temporal-middle in the left hemisphere generated by parcellation of the white surface using Destrieux (a2009s) parcellation
1017	27367	Area of Lat-Fis-ant-Horizont in the left hemisphere generated by parcellation of the white surface using Destrieux (a2009s) parcellation
1018	27368	Area of Lat-Fis-ant-Vertical in the left hemisphere generated by parcellation of the white surface using Destrieux (a2009s) parcellation
1019	27369	Area of Lat-Fis-post in the left hemisphere generated by parcellation of the white surface using Destrieux (a2009s) parcellation
1020	27370	Area of Pole-occipital in the left hemisphere generated by parcellation of the white surface using Destrieux (a2009s) parcellation
1021	27371	Area of Pole-temporal in the left hemisphere generated by parcellation of the white surface using Destrieux (a2009s) parcellation
1022	27372	Area of S-calcarine in the left hemisphere generated by parcellation of the white surface using Destrieux (a2009s) parcellation
1023	27373	Area of S-central in the left hemisphere generated by parcellation of the white surface using Destrieux (a2009s) parcellation
1024	27374	Area of S-cingul-Marginalis in the left hemisphere generated by parcellation of the white surface using Destrieux (a2009s) parcellation
1025	27375	Area of S-circular-insula-ant in the left hemisphere generated by parcellation of the white surface using Destrieux (a2009s) parcellation
1026	27376	Area of S-circular-insula-inf in the left hemisphere generated by parcellation of the white surface using Destrieux (a2009s) parcellation
1027	27377	Area of S-circular-insula-sup in the left hemisphere generated by parcellation of the white surface using Destrieux (a2009s) parcellation
1028	27378	Area of S-collat-transv-ant in the left hemisphere generated by parcellation of the white surface using Destrieux (a2009s) parcellation
1029	27379	Area of S-collat-transv-post in the left hemisphere generated by parcellation of the white surface using Destrieux (a2009s) parcellation
1030	27380	Area of S-front-inf in the left hemisphere generated by parcellation of the white surface using Destrieux (a2009s) parcellation
1031	27381	Area of S-front-middle in the left hemisphere generated by parcellation of the white surface using Destrieux (a2009s) parcellation
1032	27382	Area of S-front-sup in the left hemisphere generated by parcellation of the white surface using Destrieux (a2009s) parcellation
1033	27383	Area of S-interm-prim-Jensen in the left hemisphere generated by parcellation of the white surface using Destrieux (a2009s) parcellation
1034	27384	Area of S-intrapariet+P-trans in the left hemisphere generated by parcellation of the white surface using Destrieux (a2009s) parcellation
1035	27385	Area of S-oc-middle+Lunatus in the left hemisphere generated by parcellation of the white surface using Destrieux (a2009s) parcellation
1036	27386	Area of S-oc-sup+transversal in the left hemisphere generated by parcellation of the white surface using Destrieux (a2009s) parcellation
1037	27387	Area of S-occipital-ant in the left hemisphere generated by parcellation of the white surface using Destrieux (a2009s) parcellation
1038	27388	Area of S-oc-temp-lat in the left hemisphere generated by parcellation of the white surface using Destrieux (a2009s) parcellation
1039	27389	Area of S-oc-temp-med+Lingual in the left hemisphere generated by parcellation of the white surface using Destrieux (a2009s) parcellation
1040	27390	Area of S-orbital-lateral in the left hemisphere generated by parcellation of the white surface using Destrieux (a2009s) parcellation
1041	27391	Area of S-orbital-med-olfact in the left hemisphere generated by parcellation of the white surface using Destrieux (a2009s) parcellation
1042	27392	Area of S-orbital-H-Shaped in the left hemisphere generated by parcellation of the white surface using Destrieux (a2009s) parcellation
1043	27393	Area of S-parieto-occipital in the left hemisphere generated by parcellation of the white surface using Destrieux (a2009s) parcellation
1044	27394	Area of S-pericallosal in the left hemisphere generated by parcellation of the white surface using Destrieux (a2009s) parcellation
1045	27395	Area of S-postcentral in the left hemisphere generated by parcellation of the white surface using Destrieux (a2009s) parcellation
1046	27396	Area of S-precentral-inf-part in the left hemisphere generated by parcellation of the white surface using Destrieux (a2009s) parcellation
1047	27397	Area of S-precentral-sup-part in the left hemisphere generated by parcellation of the white surface using Destrieux (a2009s) parcellation
1048	27398	Area of S-suborbital in the left hemisphere generated by parcellation of the white surface using Destrieux (a2009s) parcellation
1049	27399	Area of S-subparietal in the left hemisphere generated by parcellation of the white surface using Destrieux (a2009s) parcellation
1050	27400	Area of S-temporal-inf in the left hemisphere generated by parcellation of the white surface using Destrieux (a2009s) parcellation
1051	27401	Area of S-temporal-sup in the left hemisphere generated by parcellation of the white surface using Destrieux (a2009s) parcellation
1052	27402	Area of S-temporal-transverse in the left hemisphere generated by parcellation of the white surface using Destrieux (a2009s) parcellation
1053	27403	Mean thickness of G+S-frontomargin in the left hemisphere generated by parcellation of the white surface using Destrieux (a2009s) parcellation
1054	27404	Mean thickness of G+S-occipital-inf in the left hemisphere generated by parcellation of the white surface using Destrieux (a2009s) parcellation
1055	27405	Mean thickness of G+S-paracentral in the left hemisphere generated by parcellation of the white surface using Destrieux (a2009s) parcellation
1056	27406	Mean thickness of G+S-subcentral in the left hemisphere generated by parcellation of the white surface using Destrieux (a2009s) parcellation
1057	27407	Mean thickness of G+S-transv-frontopol in the left hemisphere generated by parcellation of the white surface using Destrieux (a2009s) parcellation
1058	27408	Mean thickness of G+S-cingul-Ant in the left hemisphere generated by parcellation of the white surface using Destrieux (a2009s) parcellation
1059	27409	Mean thickness of G+S-cingul-Mid-Ant in the left hemisphere generated by parcellation of the white surface using Destrieux (a2009s) parcellation
1060	27410	Mean thickness of G+S-cingul-Mid-Post in the left hemisphere generated by parcellation of the white surface using Destrieux (a2009s) parcellation
1061	27411	Mean thickness of G-cingul-Post-dorsal in the left hemisphere generated by parcellation of the white surface using Destrieux (a2009s) parcellation
1062	27412	Mean thickness of G-cingul-Post-ventral in the left hemisphere generated by parcellation of the white surface using Destrieux (a2009s) parcellation
1063	27413	Mean thickness of G-cuneus in the left hemisphere generated by parcellation of the white surface using Destrieux (a2009s) parcellation
1064	27414	Mean thickness of G-front-inf-Opercular in the left hemisphere generated by parcellation of the white surface using Destrieux (a2009s) parcellation
1065	27415	Mean thickness of G-front-inf-Orbital in the left hemisphere generated by parcellation of the white surface using Destrieux (a2009s) parcellation
1066	27416	Mean thickness of G-front-inf-Triangul in the left hemisphere generated by parcellation of the white surface using Destrieux (a2009s) parcellation
1067	27417	Mean thickness of G-front-middle in the left hemisphere generated by parcellation of the white surface using Destrieux (a2009s) parcellation
1068	27418	Mean thickness of G-front-sup in the left hemisphere generated by parcellation of the white surface using Destrieux (a2009s) parcellation
1069	27419	Mean thickness of G-Ins-lg+S-cent-ins in the left hemisphere generated by parcellation of the white surface using Destrieux (a2009s) parcellation
1070	27420	Mean thickness of G-insular-short in the left hemisphere generated by parcellation of the white surface using Destrieux (a2009s) parcellation
1071	27421	Mean thickness of G-occipital-middle in the left hemisphere generated by parcellation of the white surface using Destrieux (a2009s) parcellation
1072	27422	Mean thickness of G-occipital-sup in the left hemisphere generated by parcellation of the white surface using Destrieux (a2009s) parcellation
1073	27423	Mean thickness of G-oc-temp-lat-fusifor in the left hemisphere generated by parcellation of the white surface using Destrieux (a2009s) parcellation
1074	27424	Mean thickness of G-oc-temp-med-Lingual in the left hemisphere generated by parcellation of the white surface using Destrieux (a2009s) parcellation
1075	27425	Mean thickness of G-oc-temp-med-Parahip in the left hemisphere generated by parcellation of the white surface using Destrieux (a2009s) parcellation
1076	27426	Mean thickness of G-orbital in the left hemisphere generated by parcellation of the white surface using Destrieux (a2009s) parcellation
1077	27427	Mean thickness of G-pariet-inf-Angular in the left hemisphere generated by parcellation of the white surface using Destrieux (a2009s) parcellation
1078	27428	Mean thickness of G-pariet-inf-Supramar in the left hemisphere generated by parcellation of the white surface using Destrieux (a2009s) parcellation
1079	27429	Mean thickness of G-parietal-sup in the left hemisphere generated by parcellation of the white surface using Destrieux (a2009s) parcellation
1080	27430	Mean thickness of G-postcentral in the left hemisphere generated by parcellation of the white surface using Destrieux (a2009s) parcellation
1081	27431	Mean thickness of G-precentral in the left hemisphere generated by parcellation of the white surface using Destrieux (a2009s) parcellation
1082	27432	Mean thickness of G-precuneus in the left hemisphere generated by parcellation of the white surface using Destrieux (a2009s) parcellation
1083	27433	Mean thickness of G-rectus in the left hemisphere generated by parcellation of the white surface using Destrieux (a2009s) parcellation
1084	27434	Mean thickness of G-subcallosal in the left hemisphere generated by parcellation of the white surface using Destrieux (a2009s) parcellation
1085	27435	Mean thickness of G-temp-sup-G-T-transv in the left hemisphere generated by parcellation of the white surface using Destrieux (a2009s) parcellation
1086	27436	Mean thickness of G-temp-sup-Lateral in the left hemisphere generated by parcellation of the white surface using Destrieux (a2009s) parcellation
1087	27437	Mean thickness of G-temp-sup-Plan-polar in the left hemisphere generated by parcellation of the white surface using Destrieux (a2009s) parcellation
1088	27438	Mean thickness of G-temp-sup-Plan-tempo in the left hemisphere generated by parcellation of the white surface using Destrieux (a2009s) parcellation
1089	27439	Mean thickness of G-temporal-inf in the left hemisphere generated by parcellation of the white surface using Destrieux (a2009s) parcellation
1090	27440	Mean thickness of G-temporal-middle in the left hemisphere generated by parcellation of the white surface using Destrieux (a2009s) parcellation

1091	27441	Mean thickness of Lat-Fis-ant-Horizont in the left hemisphere generated by parcellation of the white surface using Destrieux (a2009s) parcellation
1092	27442	Mean thickness of Lat-Fis-ant-Vertical in the left hemisphere generated by parcellation of the white surface using Destrieux (a2009s) parcellation
1093	27443	Mean thickness of Lat-Fis-post in the left hemisphere generated by parcellation of the white surface using Destrieux (a2009s) parcellation
1094	27444	Mean thickness of Pole-occipital in the left hemisphere generated by parcellation of the white surface using Destrieux (a2009s) parcellation
1095	27445	Mean thickness of Pole-temporal in the left hemisphere generated by parcellation of the white surface using Destrieux (a2009s) parcellation
1096	27446	Mean thickness of S-calcarine in the left hemisphere generated by parcellation of the white surface using Destrieux (a2009s) parcellation
1097	27447	Mean thickness of S-central in the left hemisphere generated by parcellation of the white surface using Destrieux (a2009s) parcellation
1098	27448	Mean thickness of S-cingul-Marginalis in the left hemisphere generated by parcellation of the white surface using Destrieux (a2009s) parcellation
1099	27449	Mean thickness of S-circular-insula-ant in the left hemisphere generated by parcellation of the white surface using Destrieux (a2009s) parcellation
1100	27450	Mean thickness of S-circular-insula-inf in the left hemisphere generated by parcellation of the white surface using Destrieux (a2009s) parcellation
1101	27451	Mean thickness of S-circular-insula-sup in the left hemisphere generated by parcellation of the white surface using Destrieux (a2009s) parcellation
1102	27452	Mean thickness of S-collat-transv-ant in the left hemisphere generated by parcellation of the white surface using Destrieux (a2009s) parcellation
1103	27453	Mean thickness of S-collat-transv-post in the left hemisphere generated by parcellation of the white surface using Destrieux (a2009s) parcellation
1104	27454	Mean thickness of S-front-inf in the left hemisphere generated by parcellation of the white surface using Destrieux (a2009s) parcellation
1105	27455	Mean thickness of S-front-middle in the left hemisphere generated by parcellation of the white surface using Destrieux (a2009s) parcellation
1106	27456	Mean thickness of S-front-sup in the left hemisphere generated by parcellation of the white surface using Destrieux (a2009s) parcellation
1107	27457	Mean thickness of S-interm-prim-Jensen in the left hemisphere generated by parcellation of the white surface using Destrieux (a2009s) parcellation
1108	27458	Mean thickness of S-intrapariet+P-trans in the left hemisphere generated by parcellation of the white surface using Destrieux (a2009s) parcellation
1109	27459	Mean thickness of S-oc-middle+Lunatus in the left hemisphere generated by parcellation of the white surface using Destrieux (a2009s) parcellation
1110	27460	Mean thickness of S-oc-sup+transversal in the left hemisphere generated by parcellation of the white surface using Destrieux (a2009s) parcellation
1111	27461	Mean thickness of S-occipital-ant in the left hemisphere generated by parcellation of the white surface using Destrieux (a2009s) parcellation
1112	27462	Mean thickness of S-oc-temp-lat in the left hemisphere generated by parcellation of the white surface using Destrieux (a2009s) parcellation
1113	27463	Mean thickness of S-oc-temp-med+Lingual in the left hemisphere generated by parcellation of the white surface using Destrieux (a2009s) parcellation
1114	27464	Mean thickness of S-orbital-lateral in the left hemisphere generated by parcellation of the white surface using Destrieux (a2009s) parcellation
1115	27465	Mean thickness of S-orbital-med-olfact in the left hemisphere generated by parcellation of the white surface using Destrieux (a2009s) parcellation
1116	27466	Mean thickness of S-orbital-H-Shaped in the left hemisphere generated by parcellation of the white surface using Destrieux (a2009s) parcellation
1117	27467	Mean thickness of S-parieto-occipital in the left hemisphere generated by parcellation of the white surface using Destrieux (a2009s) parcellation
1118	27468	Mean thickness of S-pericallosal in the left hemisphere generated by parcellation of the white surface using Destrieux (a2009s) parcellation
1119	27469	Mean thickness of S-postcentral in the left hemisphere generated by parcellation of the white surface using Destrieux (a2009s) parcellation
1120	27470	Mean thickness of S-precentral-inf-part in the left hemisphere generated by parcellation of the white surface using Destrieux (a2009s) parcellation
1121	27471	Mean thickness of S-precentral-sup-part in the left hemisphere generated by parcellation of the white surface using Destrieux (a2009s) parcellation
1122	27472	Mean thickness of S-suborbital in the left hemisphere generated by parcellation of the white surface using Destrieux (a2009s) parcellation
1123	27473	Mean thickness of S-subparietal in the left hemisphere generated by parcellation of the white surface using Destrieux (a2009s) parcellation
1124	27474	Mean thickness of S-temporal-inf in the left hemisphere generated by parcellation of the white surface using Destrieux (a2009s) parcellation
1125	27475	Mean thickness of S-temporal-sup in the left hemisphere generated by parcellation of the white surface using Destrieux (a2009s) parcellation
1126	27476	Mean thickness of S-temporal-transverse in the left hemisphere generated by parcellation of the white surface using Destrieux (a2009s) parcellation
1127	27477	Volume of G+S-frontomargin in the left hemisphere generated by parcellation of the white surface using Destrieux (a2009s) parcellation
1128	27478	Volume of G+S-occipital-inf in the left hemisphere generated by parcellation of the white surface using Destrieux (a2009s) parcellation
1129	27479	Volume of G+S-paracentral in the left hemisphere generated by parcellation of the white surface using Destrieux (a2009s) parcellation
1130	27480	Volume of G+S-subcentral in the left hemisphere generated by parcellation of the white surface using Destrieux (a2009s) parcellation
1131	27481	Volume of G+S-transv-frontopol in the left hemisphere generated by parcellation of the white surface using Destrieux (a2009s) parcellation
1132	27482	Volume of G+S-cingul-Ant in the left hemisphere generated by parcellation of the white surface using Destrieux (a2009s) parcellation
1133	27483	Volume of G+S-cingul-Mid-Ant in the left hemisphere generated by parcellation of the white surface using Destrieux (a2009s) parcellation
1134	27484	Volume of G+S-cingul-Mid-Post in the left hemisphere generated by parcellation of the white surface using Destrieux (a2009s) parcellation
1135	27485	Volume of G-cingul-Post-dorsal in the left hemisphere generated by parcellation of the white surface using Destrieux (a2009s) parcellation
1136	27486	Volume of G-cingul-Post-ventral in the left hemisphere generated by parcellation of the white surface using Destrieux (a2009s) parcellation
1137	27487	Volume of G-cuneus in the left hemisphere generated by parcellation of the white surface using Destrieux (a2009s) parcellation
1138	27488	Volume of G-front-inf-Opercular in the left hemisphere generated by parcellation of the white surface using Destrieux (a2009s) parcellation
1139	27489	Volume of G-front-inf-Orbital in the left hemisphere generated by parcellation of the white surface using Destrieux (a2009s) parcellation
1140	27490	Volume of G-front-inf-Triangul in the left hemisphere generated by parcellation of the white surface using Destrieux (a2009s) parcellation
1141	27491	Volume of G-front-middle in the left hemisphere generated by parcellation of the white surface using Destrieux (a2009s) parcellation
1142	27492	Volume of G-front-sup in the left hemisphere generated by parcellation of the white surface using Destrieux (a2009s) parcellation
1143	27493	Volume of G-Ins-lg+S-cent-ins in the left hemisphere generated by parcellation of the white surface using Destrieux (a2009s) parcellation
1144	27494	Volume of G-insular-short in the left hemisphere generated by parcellation of the white surface using Destrieux (a2009s) parcellation
1145	27495	Volume of G-occipital-middle in the left hemisphere generated by parcellation of the white surface using Destrieux (a2009s) parcellation
1146	27496	Volume of G-occipital-sup in the left hemisphere generated by parcellation of the white surface using Destrieux (a2009s) parcellation
1147	27497	Volume of G-oc-temp-lat-fusifor in the left hemisphere generated by parcellation of the white surface using Destrieux (a2009s) parcellation
1148	27498	Volume of G-oc-temp-med-Lingual in the left hemisphere generated by parcellation of the white surface using Destrieux (a2009s) parcellation
1149	27499	Volume of G-oc-temp-med-Parahip in the left hemisphere generated by parcellation of the white surface using Destrieux (a2009s) parcellation
1150	27500	Volume of G-orbital in the left hemisphere generated by parcellation of the white surface using Destrieux (a2009s) parcellation
1151	27501	Volume of G-pariet-inf-Angular in the left hemisphere generated by parcellation of the white surface using Destrieux (a2009s) parcellation
1152	27502	Volume of G-pariet-inf-Supramar in the left hemisphere generated by parcellation of the white surface using Destrieux (a2009s) parcellation
1153	27503	Volume of G-parietal-sup in the left hemisphere generated by parcellation of the white surface using Destrieux (a2009s) parcellation
1154	27504	Volume of G-postcentral in the left hemisphere generated by parcellation of the white surface using Destrieux (a2009s) parcellation
1155	27505	Volume of G-precentral in the left hemisphere generated by parcellation of the white surface using Destrieux (a2009s) parcellation
1156	27506	Volume of G-precuneus in the left hemisphere generated by parcellation of the white surface using Destrieux (a2009s) parcellation
1157	27507	Volume of G-rectus in the left hemisphere generated by parcellation of the white surface using Destrieux (a2009s) parcellation
1158	27508	Volume of G-subcallosal in the left hemisphere generated by parcellation of the white surface using Destrieux (a2009s) parcellation
1159	27509	Volume of G-temp-sup-G-T-transv in the left hemisphere generated by parcellation of the white surface using Destrieux (a2009s) parcellation
1160	27510	Volume of G-temp-sup-Lateral in the left hemisphere generated by parcellation of the white surface using Destrieux (a2009s) parcellation
1161	27511	Volume of G-temp-sup-Plan-polar in the left hemisphere generated by parcellation of the white surface using Destrieux (a2009s) parcellation
1162	27512	Volume of G-temp-sup-Plan-tempo in the left hemisphere generated by parcellation of the white surface using Destrieux (a2009s) parcellation
1163	27513	Volume of G-temporal-inf in the left hemisphere generated by parcellation of the white surface using Destrieux (a2009s) parcellation
1164	27514	Volume of G-temporal-middle in the left hemisphere generated by parcellation of the white surface using Destrieux (a2009s) parcellation
1165	27515	Volume of Lat-Fis-ant-Horizont in the left hemisphere generated by parcellation of the white surface using Destrieux (a2009s) parcellation
1166	27516	Volume of Lat-Fis-ant-Vertical in the left hemisphere generated by parcellation of the white surface using Destrieux (a2009s) parcellation
1167	27517	Volume of Lat-Fis-post in the left hemisphere generated by parcellation of the white surface using Destrieux (a2009s) parcellation
1168	27518	Volume of Pole-occipital in the left hemisphere generated by parcellation of the white surface using Destrieux (a2009s) parcellation
1169	27519	Volume of Pole-temporal in the left hemisphere generated by parcellation of the white surface using Destrieux (a2009s) parcellation
1170	27520	Volume of S-calcarine in the left hemisphere generated by parcellation of the white surface using Destrieux (a2009s) parcellation
1171	27521	Volume of S-central in the left hemisphere generated by parcellation of the white surface using Destrieux (a2009s) parcellation
1172	27522	Volume of S-cingul-Marginalis in the left hemisphere generated by parcellation of the white surface using Destrieux (a2009s) parcellation
1173	27523	Volume of S-circular-insula-ant in the left hemisphere generated by parcellation of the white surface using Destrieux (a2009s) parcellation
1174	27524	Volume of S-circular-insula-inf in the left hemisphere generated by parcellation of the white surface using Destrieux (a2009s) parcellation
1175	27525	Volume of S-circular-insula-sup in the left hemisphere generated by parcellation of the white surface using Destrieux (a2009s) parcellation
1176	27526	Volume of S-collat-transv-ant in the left hemisphere generated by parcellation of the white surface using Destrieux (a2009s) parcellation
1177	27527	Volume of S-collat-transv-post in the left hemisphere generated by parcellation of the white surface using Destrieux (a2009s) parcellation
1178	27528	Volume of S-front-inf in the left hemisphere generated by parcellation of the white surface using Destrieux (a2009s) parcellation
1179	27529	Volume of S-front-middle in the left hemisphere generated by parcellation of the white surface using Destrieux (a2009s) parcellation

1180	27530	Volume of S-front-sup in the left hemisphere generated by parcellation of the white surface using Destrieux (a2009s) parcellation
1181	27531	Volume of S-interm-prim-Jensen in the left hemisphere generated by parcellation of the white surface using Destrieux (a2009s) parcellation
1182	27532	Volume of S-intrapariet+P-trans in the left hemisphere generated by parcellation of the white surface using Destrieux (a2009s) parcellation
1183	27533	Volume of S-oc-middle+Lunatus in the left hemisphere generated by parcellation of the white surface using Destrieux (a2009s) parcellation
1184	27534	Volume of S-oc-sup+transversal in the left hemisphere generated by parcellation of the white surface using Destrieux (a2009s) parcellation
1185	27535	Volume of S-occipital-ant in the left hemisphere generated by parcellation of the white surface using Destrieux (a2009s) parcellation
1186	27536	Volume of S-oc-temp-lat in the left hemisphere generated by parcellation of the white surface using Destrieux (a2009s) parcellation
1187	27537	Volume of S-oc-temp-med+Lingual in the left hemisphere generated by parcellation of the white surface using Destrieux (a2009s) parcellation
1188	27538	Volume of S-orbital-lateral in the left hemisphere generated by parcellation of the white surface using Destrieux (a2009s) parcellation
1189	27539	Volume of S-orbital-med-olfact in the left hemisphere generated by parcellation of the white surface using Destrieux (a2009s) parcellation
1190	27540	Volume of S-orbital-H-Shaped in the left hemisphere generated by parcellation of the white surface using Destrieux (a2009s) parcellation
1191	27541	Volume of S-parieto-occipital in the left hemisphere generated by parcellation of the white surface using Destrieux (a2009s) parcellation
1192	27542	Volume of S-pericallosal in the left hemisphere generated by parcellation of the white surface using Destrieux (a2009s) parcellation
1193	27543	Volume of S-postcentral in the left hemisphere generated by parcellation of the white surface using Destrieux (a2009s) parcellation
1194	27544	Volume of S-precentral-inf-part in the left hemisphere generated by parcellation of the white surface using Destrieux (a2009s) parcellation
1195	27545	Volume of S-precentral-sup-part in the left hemisphere generated by parcellation of the white surface using Destrieux (a2009s) parcellation
1196	27546	Volume of S-suborbital in the left hemisphere generated by parcellation of the white surface using Destrieux (a2009s) parcellation
1197	27547	Volume of S-subparietal in the left hemisphere generated by parcellation of the white surface using Destrieux (a2009s) parcellation
1198	27548	Volume of S-temporal-inf in the left hemisphere generated by parcellation of the white surface using Destrieux (a2009s) parcellation
1199	27549	Volume of S-temporal-sup in the left hemisphere generated by parcellation of the white surface using Destrieux (a2009s) parcellation
1200	27550	Volume of S-temporal-transverse in the left hemisphere generated by parcellation of the white surface using Destrieux (a2009s) parcellation
1201	27551	Area of G+S-frontomargin in the right hemisphere generated by parcellation of the white surface using Destrieux (a2009s) parcellation
1202	27552	Area of G+S-occipital-inf in the right hemisphere generated by parcellation of the white surface using Destrieux (a2009s) parcellation
1203	27553	Area of G+S-paracentral in the right hemisphere generated by parcellation of the white surface using Destrieux (a2009s) parcellation
1204	27554	Area of G+S-subcentral in the right hemisphere generated by parcellation of the white surface using Destrieux (a2009s) parcellation
1205	27555	Area of G+S-transv-frontopol in the right hemisphere generated by parcellation of the white surface using Destrieux (a2009s) parcellation
1206	27556	Area of G+S-cingul-Ant in the right hemisphere generated by parcellation of the white surface using Destrieux (a2009s) parcellation
1207	27557	Area of G+S-cingul-Mid-Ant in the right hemisphere generated by parcellation of the white surface using Destrieux (a2009s) parcellation
1208	27558	Area of G+S-cingul-Mid-Post in the right hemisphere generated by parcellation of the white surface using Destrieux (a2009s) parcellation
1209	27559	Area of G-cingul-Post-dorsal in the right hemisphere generated by parcellation of the white surface using Destrieux (a2009s) parcellation
1210	27560	Area of G-cingul-Post-ventral in the right hemisphere generated by parcellation of the white surface using Destrieux (a2009s) parcellation
1211	27561	Area of G-cuneus in the right hemisphere generated by parcellation of the white surface using Destrieux (a2009s) parcellation
1212	27562	Area of G-front-inf-Opercular in the right hemisphere generated by parcellation of the white surface using Destrieux (a2009s) parcellation
1213	27563	Area of G-front-inf-Orbital in the right hemisphere generated by parcellation of the white surface using Destrieux (a2009s) parcellation
1214	27564	Area of G-front-inf-Triangul in the right hemisphere generated by parcellation of the white surface using Destrieux (a2009s) parcellation
1215	27565	Area of G-front-middle in the right hemisphere generated by parcellation of the white surface using Destrieux (a2009s) parcellation
1216	27566	Area of G-front-sup in the right hemisphere generated by parcellation of the white surface using Destrieux (a2009s) parcellation
1217	27567	Area of G-Ins-lg+S-cent-ins in the right hemisphere generated by parcellation of the white surface using Destrieux (a2009s) parcellation
1218	27568	Area of G-insular-short in the right hemisphere generated by parcellation of the white surface using Destrieux (a2009s) parcellation
1219	27569	Area of G-occipital-middle in the right hemisphere generated by parcellation of the white surface using Destrieux (a2009s) parcellation
1220	27570	Area of G-occipital-sup in the right hemisphere generated by parcellation of the white surface using Destrieux (a2009s) parcellation
1221	27571	Area of G-oc-temp-lat-fusifor in the right hemisphere generated by parcellation of the white surface using Destrieux (a2009s) parcellation
1222	27572	Area of G-oc-temp-med-Lingual in the right hemisphere generated by parcellation of the white surface using Destrieux (a2009s) parcellation
1223	27573	Area of G-oc-temp-med-Parahip in the right hemisphere generated by parcellation of the white surface using Destrieux (a2009s) parcellation
1224	27574	Area of G-orbital in the right hemisphere generated by parcellation of the white surface using Destrieux (a2009s) parcellation
1225	27575	Area of G-pariet-inf-Angular in the right hemisphere generated by parcellation of the white surface using Destrieux (a2009s) parcellation
1226	27576	Area of G-pariet-inf-Supramar in the right hemisphere generated by parcellation of the white surface using Destrieux (a2009s) parcellation
1227	27577	Area of G-parietal-sup in the right hemisphere generated by parcellation of the white surface using Destrieux (a2009s) parcellation
1228	27578	Area of G-postcentral in the right hemisphere generated by parcellation of the white surface using Destrieux (a2009s) parcellation
1229	27579	Area of G-precentral in the right hemisphere generated by parcellation of the white surface using Destrieux (a2009s) parcellation
1230	27580	Area of G-precuneus in the right hemisphere generated by parcellation of the white surface using Destrieux (a2009s) parcellation
1231	27581	Area of G-rectus in the right hemisphere generated by parcellation of the white surface using Destrieux (a2009s) parcellation
1232	27582	Area of G-subcallosal in the right hemisphere generated by parcellation of the white surface using Destrieux (a2009s) parcellation
1233	27583	Area of G-temp-sup-G-T-transv in the right hemisphere generated by parcellation of the white surface using Destrieux (a2009s) parcellation
1234	27584	Area of G-temp-sup-Lateral in the right hemisphere generated by parcellation of the white surface using Destrieux (a2009s) parcellation
1235	27585	Area of G-temp-sup-Plan-polar in the right hemisphere generated by parcellation of the white surface using Destrieux (a2009s) parcellation
1236	27586	Area of G-temp-sup-Plan-tempo in the right hemisphere generated by parcellation of the white surface using Destrieux (a2009s) parcellation
1237	27587	Area of G-temporal-inf in the right hemisphere generated by parcellation of the white surface using Destrieux (a2009s) parcellation
1238	27588	Area of G-temporal-middle in the right hemisphere generated by parcellation of the white surface using Destrieux (a2009s) parcellation
1239	27589	Area of Lat-Fis-ant-Horizont in the right hemisphere generated by parcellation of the white surface using Destrieux (a2009s) parcellation
1240	27590	Area of Lat-Fis-ant-Vertical in the right hemisphere generated by parcellation of the white surface using Destrieux (a2009s) parcellation
1241	27591	Area of Lat-Fis-post in the right hemisphere generated by parcellation of the white surface using Destrieux (a2009s) parcellation
1242	27592	Area of Pole-occipital in the right hemisphere generated by parcellation of the white surface using Destrieux (a2009s) parcellation
1243	27593	Area of Pole-temporal in the right hemisphere generated by parcellation of the white surface using Destrieux (a2009s) parcellation
1244	27594	Area of S-calcarine in the right hemisphere generated by parcellation of the white surface using Destrieux (a2009s) parcellation
1245	27595	Area of S-central in the right hemisphere generated by parcellation of the white surface using Destrieux (a2009s) parcellation
1246	27596	Area of S-cingul-Marginalis in the right hemisphere generated by parcellation of the white surface using Destrieux (a2009s) parcellation
1247	27597	Area of S-circular-insula-ant in the right hemisphere generated by parcellation of the white surface using Destrieux (a2009s) parcellation
1248	27598	Area of S-circular-insula-inf in the right hemisphere generated by parcellation of the white surface using Destrieux (a2009s) parcellation
1249	27599	Area of S-circular-insula-sup in the right hemisphere generated by parcellation of the white surface using Destrieux (a2009s) parcellation
1250	27600	Area of S-collat-transv-ant in the right hemisphere generated by parcellation of the white surface using Destrieux (a2009s) parcellation
1251	27601	Area of S-collat-transv-post in the right hemisphere generated by parcellation of the white surface using Destrieux (a2009s) parcellation
1252	27602	Area of S-front-inf in the right hemisphere generated by parcellation of the white surface using Destrieux (a2009s) parcellation
1253	27603	Area of S-front-middle in the right hemisphere generated by parcellation of the white surface using Destrieux (a2009s) parcellation
1254	27604	Area of S-front-sup in the right hemisphere generated by parcellation of the white surface using Destrieux (a2009s) parcellation
1255	27605	Area of S-interm-prim-Jensen in the right hemisphere generated by parcellation of the white surface using Destrieux (a2009s) parcellation
1256	27606	Area of S-intrapariet+P-trans in the right hemisphere generated by parcellation of the white surface using Destrieux (a2009s) parcellation
1257	27607	Area of S-oc-middle+Lunatus in the right hemisphere generated by parcellation of the white surface using Destrieux (a2009s) parcellation
1258	27608	Area of S-oc-sup+transversal in the right hemisphere generated by parcellation of the white surface using Destrieux (a2009s) parcellation
1259	27609	Area of S-occipital-ant in the right hemisphere generated by parcellation of the white surface using Destrieux (a2009s) parcellation
1260	27610	Area of S-oc-temp-lat in the right hemisphere generated by parcellation of the white surface using Destrieux (a2009s) parcellation
1261	27611	Area of S-oc-temp-med+Lingual in the right hemisphere generated by parcellation of the white surface using Destrieux (a2009s) parcellation
1262	27612	Area of S-orbital-lateral in the right hemisphere generated by parcellation of the white surface using Destrieux (a2009s) parcellation
1263	27613	Area of S-orbital-med-olfact in the right hemisphere generated by parcellation of the white surface using Destrieux (a2009s) parcellation
1264	27614	Area of S-orbital-H-Shaped in the right hemisphere generated by parcellation of the white surface using Destrieux (a2009s) parcellation
1265	27615	Area of S-parieto-occipital in the right hemisphere generated by parcellation of the white surface using Destrieux (a2009s) parcellation
1266	27616	Area of S-pericallosal in the right hemisphere generated by parcellation of the white surface using Destrieux (a2009s) parcellation
1267	27617	Area of S-postcentral in the right hemisphere generated by parcellation of the white surface using Destrieux (a2009s) parcellation
1268	27618	Area of S-precentral-inf-part in the right hemisphere generated by parcellation of the white surface using Destrieux (a2009s) parcellation

1269	27619	Area of S-precentral-sup-part in the right hemisphere generated by parcellation of the white surface using Destrieux (a2009s) parcellation
1270	27620	Area of S-suborbital in the right hemisphere generated by parcellation of the white surface using Destrieux (a2009s) parcellation
1271	27621	Area of S-subparietal in the right hemisphere generated by parcellation of the white surface using Destrieux (a2009s) parcellation
1272	27622	Area of S-temporal-inf in the right hemisphere generated by parcellation of the white surface using Destrieux (a2009s) parcellation
1273	27623	Area of S-temporal-sup in the right hemisphere generated by parcellation of the white surface using Destrieux (a2009s) parcellation
1274	27624	Area of S-temporal-transverse in the right hemisphere generated by parcellation of the white surface using Destrieux (a2009s) parcellation
1275	27625	Mean thickness of G+S-frontomargin in the right hemisphere generated by parcellation of the white surface using Destrieux (a2009s) parcellation
1276	27626	Mean thickness of G+S-occipital-inf in the right hemisphere generated by parcellation of the white surface using Destrieux (a2009s) parcellation
1277	27627	Mean thickness of G+S-paracentral in the right hemisphere generated by parcellation of the white surface using Destrieux (a2009s) parcellation
1278	27628	Mean thickness of G+S-subcentral in the right hemisphere generated by parcellation of the white surface using Destrieux (a2009s) parcellation
1279	27629	Mean thickness of G+S-transv-frontopol in the right hemisphere generated by parcellation of the white surface using Destrieux (a2009s) parcellation
1280	27630	Mean thickness of G+S-cingul-Ant in the right hemisphere generated by parcellation of the white surface using Destrieux (a2009s) parcellation
1281	27631	Mean thickness of G+S-cingul-Mid-Ant in the right hemisphere generated by parcellation of the white surface using Destrieux (a2009s) parcellation
1282	27632	Mean thickness of G+S-cingul-Mid-Post in the right hemisphere generated by parcellation of the white surface using Destrieux (a2009s) parcellation
1283	27633	Mean thickness of G-cingul-Post-dorsal in the right hemisphere generated by parcellation of the white surface using Destrieux (a2009s) parcellation
1284	27634	Mean thickness of G-cingul-Post-ventral in the right hemisphere generated by parcellation of the white surface using Destrieux (a2009s) parcellation
1285	27635	Mean thickness of G-cuneus in the right hemisphere generated by parcellation of the white surface using Destrieux (a2009s) parcellation
1286	27636	Mean thickness of G-front-inf-Opercular in the right hemisphere generated by parcellation of the white surface using Destrieux (a2009s) parcellation
1287	27637	Mean thickness of G-front-inf-Orbital in the right hemisphere generated by parcellation of the white surface using Destrieux (a2009s) parcellation
1288	27638	Mean thickness of G-front-inf-Triangul in the right hemisphere generated by parcellation of the white surface using Destrieux (a2009s) parcellation
1289	27639	Mean thickness of G-front-middle in the right hemisphere generated by parcellation of the white surface using Destrieux (a2009s) parcellation
1290	27640	Mean thickness of G-front-sup in the right hemisphere generated by parcellation of the white surface using Destrieux (a2009s) parcellation
1291	27641	Mean thickness of G-Ins-lg+S-cent-ins in the right hemisphere generated by parcellation of the white surface using Destrieux (a2009s) parcellation
1292	27642	Mean thickness of G-insular-short in the right hemisphere generated by parcellation of the white surface using Destrieux (a2009s) parcellation
1293	27643	Mean thickness of G-occipital-middle in the right hemisphere generated by parcellation of the white surface using Destrieux (a2009s) parcellation
1294	27644	Mean thickness of G-occipital-sup in the right hemisphere generated by parcellation of the white surface using Destrieux (a2009s) parcellation
1295	27645	Mean thickness of G-oc-temp-lat-fusifor in the right hemisphere generated by parcellation of the white surface using Destrieux (a2009s) parcellation
1296	27646	Mean thickness of G-oc-temp-med-Lingual in the right hemisphere generated by parcellation of the white surface using Destrieux (a2009s) parcellation
1297	27647	Mean thickness of G-oc-temp-med-Parahip in the right hemisphere generated by parcellation of the white surface using Destrieux (a2009s) parcellation
1298	27648	Mean thickness of G-orbital in the right hemisphere generated by parcellation of the white surface using Destrieux (a2009s) parcellation
1299	27649	Mean thickness of G-pariet-inf-Angular in the right hemisphere generated by parcellation of the white surface using Destrieux (a2009s) parcellation
1300	27650	Mean thickness of G-pariet-inf-Supramar in the right hemisphere generated by parcellation of the white surface using Destrieux (a2009s) parcellation
1301	27651	Mean thickness of G-parietal-sup in the right hemisphere generated by parcellation of the white surface using Destrieux (a2009s) parcellation
1302	27652	Mean thickness of G-postcentral in the right hemisphere generated by parcellation of the white surface using Destrieux (a2009s) parcellation
1303	27653	Mean thickness of G-precentral in the right hemisphere generated by parcellation of the white surface using Destrieux (a2009s) parcellation
1304	27654	Mean thickness of G-precuneus in the right hemisphere generated by parcellation of the white surface using Destrieux (a2009s) parcellation
1305	27655	Mean thickness of G-rectus in the right hemisphere generated by parcellation of the white surface using Destrieux (a2009s) parcellation
1306	27656	Mean thickness of G-subcallosal in the right hemisphere generated by parcellation of the white surface using Destrieux (a2009s) parcellation
1307	27657	Mean thickness of G-temp-sup-G-T-transv in the right hemisphere generated by parcellation of the white surface using Destrieux (a2009s) parcellation
1308	27658	Mean thickness of G-temp-sup-Lateral in the right hemisphere generated by parcellation of the white surface using Destrieux (a2009s) parcellation
1309	27659	Mean thickness of G-temp-sup-Plan-polar in the right hemisphere generated by parcellation of the white surface using Destrieux (a2009s) parcellation
1310	27660	Mean thickness of G-temp-sup-Plan-tempo in the right hemisphere generated by parcellation of the white surface using Destrieux (a2009s) parcellation
1311	27661	Mean thickness of G-temporal-inf in the right hemisphere generated by parcellation of the white surface using Destrieux (a2009s) parcellation
1312	27662	Mean thickness of G-temporal-middle in the right hemisphere generated by parcellation of the white surface using Destrieux (a2009s) parcellation
1313	27663	Mean thickness of Lat-Fis-ant-Horizont in the right hemisphere generated by parcellation of the white surface using Destrieux (a2009s) parcellation
1314	27664	Mean thickness of Lat-Fis-ant-Vertical in the right hemisphere generated by parcellation of the white surface using Destrieux (a2009s) parcellation
1315	27665	Mean thickness of Lat-Fis-post in the right hemisphere generated by parcellation of the white surface using Destrieux (a2009s) parcellation
1316	27666	Mean thickness of Pole-occipital in the right hemisphere generated by parcellation of the white surface using Destrieux (a2009s) parcellation
1317	27667	Mean thickness of Pole-temporal in the right hemisphere generated by parcellation of the white surface using Destrieux (a2009s) parcellation
1318	27668	Mean thickness of S-calcarine in the right hemisphere generated by parcellation of the white surface using Destrieux (a2009s) parcellation
1319	27669	Mean thickness of S-central in the right hemisphere generated by parcellation of the white surface using Destrieux (a2009s) parcellation
1320	27670	Mean thickness of S-cingul-Marginalis in the right hemisphere generated by parcellation of the white surface using Destrieux (a2009s) parcellation
1321	27671	Mean thickness of S-circular-insula-ant in the right hemisphere generated by parcellation of the white surface using Destrieux (a2009s) parcellation
1322	27672	Mean thickness of S-circular-insula-inf in the right hemisphere generated by parcellation of the white surface using Destrieux (a2009s) parcellation
1323	27673	Mean thickness of S-circular-insula-sup in the right hemisphere generated by parcellation of the white surface using Destrieux (a2009s) parcellation
1324	27674	Mean thickness of S-collat-transv-ant in the right hemisphere generated by parcellation of the white surface using Destrieux (a2009s) parcellation
1325	27675	Mean thickness of S-collat-transv-post in the right hemisphere generated by parcellation of the white surface using Destrieux (a2009s) parcellation
1326	27676	Mean thickness of S-front-inf in the right hemisphere generated by parcellation of the white surface using Destrieux (a2009s) parcellation
1327	27677	Mean thickness of S-front-middle in the right hemisphere generated by parcellation of the white surface using Destrieux (a2009s) parcellation
1328	27678	Mean thickness of S-front-sup in the right hemisphere generated by parcellation of the white surface using Destrieux (a2009s) parcellation
1329	27679	Mean thickness of S-interm-prim-Jensen in the right hemisphere generated by parcellation of the white surface using Destrieux (a2009s) parcellation
1330	27680	Mean thickness of S-intrapariet+P-trans in the right hemisphere generated by parcellation of the white surface using Destrieux (a2009s) parcellation
1331	27681	Mean thickness of S-oc-middle+Lunatus in the right hemisphere generated by parcellation of the white surface using Destrieux (a2009s) parcellation
1332	27682	Mean thickness of S-oc-sup+transversal in the right hemisphere generated by parcellation of the white surface using Destrieux (a2009s) parcellation
1333	27683	Mean thickness of S-occipital-ant in the right hemisphere generated by parcellation of the white surface using Destrieux (a2009s) parcellation
1334	27684	Mean thickness of S-oc-temp-lat in the right hemisphere generated by parcellation of the white surface using Destrieux (a2009s) parcellation
1335	27685	Mean thickness of S-oc-temp-med+Lingual in the right hemisphere generated by parcellation of the white surface using Destrieux (a2009s) parcellation
1336	27686	Mean thickness of S-orbital-lateral in the right hemisphere generated by parcellation of the white surface using Destrieux (a2009s) parcellation
1337	27687	Mean thickness of S-orbital-med-olfact in the right hemisphere generated by parcellation of the white surface using Destrieux (a2009s) parcellation
1338	27688	Mean thickness of S-orbital-H-Shaped in the right hemisphere generated by parcellation of the white surface using Destrieux (a2009s) parcellation
1339	27689	Mean thickness of S-parieto-occipital in the right hemisphere generated by parcellation of the white surface using Destrieux (a2009s) parcellation
1340	27690	Mean thickness of S-pericallosal in the right hemisphere generated by parcellation of the white surface using Destrieux (a2009s) parcellation
1341	27691	Mean thickness of S-postcentral in the right hemisphere generated by parcellation of the white surface using Destrieux (a2009s) parcellation
1342	27692	Mean thickness of S-precentral-inf-part in the right hemisphere generated by parcellation of the white surface using Destrieux (a2009s) parcellation
1343	27693	Mean thickness of S-precentral-sup-part in the right hemisphere generated by parcellation of the white surface using Destrieux (a2009s) parcellation
1344	27694	Mean thickness of S-suborbital in the right hemisphere generated by parcellation of the white surface using Destrieux (a2009s) parcellation
1345	27695	Mean thickness of S-subparietal in the right hemisphere generated by parcellation of the white surface using Destrieux (a2009s) parcellation
1346	27696	Mean thickness of S-temporal-inf in the right hemisphere generated by parcellation of the white surface using Destrieux (a2009s) parcellation
1347	27697	Mean thickness of S-temporal-sup in the right hemisphere generated by parcellation of the white surface using Destrieux (a2009s) parcellation
1348	27698	Mean thickness of S-temporal-transverse in the right hemisphere generated by parcellation of the white surface using Destrieux (a2009s) parcellation
1349	27699	Volume of G+S-frontomargin in the right hemisphere generated by parcellation of the white surface using Destrieux (a2009s) parcellation
1350	27700	Volume of G+S-occipital-inf in the right hemisphere generated by parcellation of the white surface using Destrieux (a2009s) parcellation
1351	27701	Volume of G+S-paracentral in the right hemisphere generated by parcellation of the white surface using Destrieux (a2009s) parcellation
1352	27702	Volume of G+S-subcentral in the right hemisphere generated by parcellation of the white surface using Destrieux (a2009s) parcellation
1353	27703	Volume of G+S-transv-frontopol in the right hemisphere generated by parcellation of the white surface using Destrieux (a2009s) parcellation
1354	27704	Volume of G+S-cingul-Ant in the right hemisphere generated by parcellation of the white surface using Destrieux (a2009s) parcellation
1355	27705	Volume of G+S-cingul-Mid-Ant in the right hemisphere generated by parcellation of the white surface using Destrieux (a2009s) parcellation
1356	27706	Volume of G+S-cingul-Mid-Post in the right hemisphere generated by parcellation of the white surface using Destrieux (a2009s) parcellation
1357	27707	Volume of G-cingul-Post-dorsal in the right hemisphere generated by parcellation of the white surface using Destrieux (a2009s) parcellation

1358	27708	Volume of G-cingul-Post-ventral in the right hemisphere generated by parcellation of the white surface using Destrieux (a2009s) parcellation
1359	27709	Volume of G-cuneus in the right hemisphere generated by parcellation of the white surface using Destrieux (a2009s) parcellation
1360	27710	Volume of G-front-inf-Opercular in the right hemisphere generated by parcellation of the white surface using Destrieux (a2009s) parcellation
1361	27711	Volume of G-front-inf-Orbital in the right hemisphere generated by parcellation of the white surface using Destrieux (a2009s) parcellation
1362	27712	Volume of G-front-inf-Triangul in the right hemisphere generated by parcellation of the white surface using Destrieux (a2009s) parcellation
1363	27713	Volume of G-front-middle in the right hemisphere generated by parcellation of the white surface using Destrieux (a2009s) parcellation
1364	27714	Volume of G-front-sup in the right hemisphere generated by parcellation of the white surface using Destrieux (a2009s) parcellation
1365	27715	Volume of G-Ins-lg+S-cent-ins in the right hemisphere generated by parcellation of the white surface using Destrieux (a2009s) parcellation
1366	27716	Volume of G-insular-short in the right hemisphere generated by parcellation of the white surface using Destrieux (a2009s) parcellation
1367	27717	Volume of G-occipital-middle in the right hemisphere generated by parcellation of the white surface using Destrieux (a2009s) parcellation
1368	27718	Volume of G-occipital-sup in the right hemisphere generated by parcellation of the white surface using Destrieux (a2009s) parcellation
1369	27719	Volume of G-oc-temp-lat-fusifor in the right hemisphere generated by parcellation of the white surface using Destrieux (a2009s) parcellation
1370	27720	Volume of G-oc-temp-med-Lingual in the right hemisphere generated by parcellation of the white surface using Destrieux (a2009s) parcellation
1371	27721	Volume of G-oc-temp-med-Parahip in the right hemisphere generated by parcellation of the white surface using Destrieux (a2009s) parcellation
1372	27722	Volume of G-orbital in the right hemisphere generated by parcellation of the white surface using Destrieux (a2009s) parcellation
1373	27723	Volume of G-pariet-inf-Angular in the right hemisphere generated by parcellation of the white surface using Destrieux (a2009s) parcellation
1374	27724	Volume of G-pariet-inf-Supramar in the right hemisphere generated by parcellation of the white surface using Destrieux (a2009s) parcellation
1375	27725	Volume of G-parietal-sup in the right hemisphere generated by parcellation of the white surface using Destrieux (a2009s) parcellation
1376	27726	Volume of G-postcentral in the right hemisphere generated by parcellation of the white surface using Destrieux (a2009s) parcellation
1377	27727	Volume of G-precentral in the right hemisphere generated by parcellation of the white surface using Destrieux (a2009s) parcellation
1378	27728	Volume of G-precuneus in the right hemisphere generated by parcellation of the white surface using Destrieux (a2009s) parcellation
1379	27729	Volume of G-rectus in the right hemisphere generated by parcellation of the white surface using Destrieux (a2009s) parcellation
1380	27730	Volume of G-subcallosal in the right hemisphere generated by parcellation of the white surface using Destrieux (a2009s) parcellation
1381	27731	Volume of G-temp-sup-G-T-transv in the right hemisphere generated by parcellation of the white surface using Destrieux (a2009s) parcellation
1382	27732	Volume of G-temp-sup-Lateral in the right hemisphere generated by parcellation of the white surface using Destrieux (a2009s) parcellation
1383	27733	Volume of G-temp-sup-Plan-polar in the right hemisphere generated by parcellation of the white surface using Destrieux (a2009s) parcellation
1384	27734	Volume of G-temp-sup-Plan-tempo in the right hemisphere generated by parcellation of the white surface using Destrieux (a2009s) parcellation
1385	27735	Volume of G-temporal-inf in the right hemisphere generated by parcellation of the white surface using Destrieux (a2009s) parcellation
1386	27736	Volume of G-temporal-middle in the right hemisphere generated by parcellation of the white surface using Destrieux (a2009s) parcellation
1387	27737	Volume of Lat-Fis-ant-Horizont in the right hemisphere generated by parcellation of the white surface using Destrieux (a2009s) parcellation
1388	27738	Volume of Lat-Fis-ant-Vertical in the right hemisphere generated by parcellation of the white surface using Destrieux (a2009s) parcellation
1389	27739	Volume of Lat-Fis-post in the right hemisphere generated by parcellation of the white surface using Destrieux (a2009s) parcellation
1390	27740	Volume of Pole-occipital in the right hemisphere generated by parcellation of the white surface using Destrieux (a2009s) parcellation
1391	27741	Volume of Pole-temporal in the right hemisphere generated by parcellation of the white surface using Destrieux (a2009s) parcellation
1392	27742	Volume of S-calcarine in the right hemisphere generated by parcellation of the white surface using Destrieux (a2009s) parcellation
1393	27743	Volume of S-central in the right hemisphere generated by parcellation of the white surface using Destrieux (a2009s) parcellation
1394	27744	Volume of S-cingul-Marginalis in the right hemisphere generated by parcellation of the white surface using Destrieux (a2009s) parcellation
1395	27745	Volume of S-circular-insula-ant in the right hemisphere generated by parcellation of the white surface using Destrieux (a2009s) parcellation
1396	27746	Volume of S-circular-insula-inf in the right hemisphere generated by parcellation of the white surface using Destrieux (a2009s) parcellation
1397	27747	Volume of S-circular-insula-sup in the right hemisphere generated by parcellation of the white surface using Destrieux (a2009s) parcellation
1398	27748	Volume of S-collat-transv-ant in the right hemisphere generated by parcellation of the white surface using Destrieux (a2009s) parcellation
1399	27749	Volume of S-collat-transv-post in the right hemisphere generated by parcellation of the white surface using Destrieux (a2009s) parcellation
1400	27750	Volume of S-front-inf in the right hemisphere generated by parcellation of the white surface using Destrieux (a2009s) parcellation
1401	27751	Volume of S-front-middle in the right hemisphere generated by parcellation of the white surface using Destrieux (a2009s) parcellation
1402	27752	Volume of S-front-sup in the right hemisphere generated by parcellation of the white surface using Destrieux (a2009s) parcellation
1403	27753	Volume of S-interm-prim-Jensen in the right hemisphere generated by parcellation of the white surface using Destrieux (a2009s) parcellation
1404	27754	Volume of S-intrapariet+P-trans in the right hemisphere generated by parcellation of the white surface using Destrieux (a2009s) parcellation
1405	27755	Volume of S-oc-middle+Lunatus in the right hemisphere generated by parcellation of the white surface using Destrieux (a2009s) parcellation
1406	27756	Volume of S-oc-sup+transversal in the right hemisphere generated by parcellation of the white surface using Destrieux (a2009s) parcellation
1407	27757	Volume of S-occipital-ant in the right hemisphere generated by parcellation of the white surface using Destrieux (a2009s) parcellation
1408	27758	Volume of S-oc-temp-lat in the right hemisphere generated by parcellation of the white surface using Destrieux (a2009s) parcellation
1409	27759	Volume of S-oc-temp-med+Lingual in the right hemisphere generated by parcellation of the white surface using Destrieux (a2009s) parcellation
1410	27760	Volume of S-orbital-lateral in the right hemisphere generated by parcellation of the white surface using Destrieux (a2009s) parcellation
1411	27761	Volume of S-orbital-med-olfact in the right hemisphere generated by parcellation of the white surface using Destrieux (a2009s) parcellation
1412	27762	Volume of S-orbital-H-Shaped in the right hemisphere generated by parcellation of the white surface using Destrieux (a2009s) parcellation
1413	27763	Volume of S-parieto-occipital in the right hemisphere generated by parcellation of the white surface using Destrieux (a2009s) parcellation
1414	27764	Volume of S-pericallosal in the right hemisphere generated by parcellation of the white surface using Destrieux (a2009s) parcellation
1415	27765	Volume of S-postcentral in the right hemisphere generated by parcellation of the white surface using Destrieux (a2009s) parcellation
1416	27766	Volume of S-precentral-inf-part in the right hemisphere generated by parcellation of the white surface using Destrieux (a2009s) parcellation
1417	27767	Volume of S-precentral-sup-part in the right hemisphere generated by parcellation of the white surface using Destrieux (a2009s) parcellation
1418	27768	Volume of S-suborbital in the right hemisphere generated by parcellation of the white surface using Destrieux (a2009s) parcellation
1419	27769	Volume of S-subparietal in the right hemisphere generated by parcellation of the white surface using Destrieux (a2009s) parcellation
1420	27770	Volume of S-temporal-inf in the right hemisphere generated by parcellation of the white surface using Destrieux (a2009s) parcellation
1421	27771	Volume of S-temporal-sup in the right hemisphere generated by parcellation of the white surface using Destrieux (a2009s) parcellation
1422	27772	Volume of S-temporal-transverse in the right hemisphere generated by parcellation of the white surface using Destrieux (a2009s) parcellation

Supplementary Table 4. List of white matter IDPs used for brain age prediction models.

No.	UKBB Data Field	IDP name
1	25056	Mean FA in middle cerebellar peduncle on FA skeleton (from dMRI data)
2	25057	Mean FA in pontine crossing tract on FA skeleton (from dMRI data)
3	25058	Mean FA in genu of corpus callosum on FA skeleton (from dMRI data)
4	25059	Mean FA in body of corpus callosum on FA skeleton (from dMRI data)
5	25060	Mean FA in splenium of corpus callosum on FA skeleton (from dMRI data)
6	25061	Mean FA in fornix on FA skeleton (from dMRI data)
7	25062	Mean FA in corticospinal tract (right) on FA skeleton (from dMRI data)
8	25063	Mean FA in corticospinal tract (left) on FA skeleton (from dMRI data)
9	25064	Mean FA in medial lemniscus (right) on FA skeleton (from dMRI data)
10	25065	Mean FA in medial lemniscus (left) on FA skeleton (from dMRI data)
11	25066	Mean FA in inferior cerebellar peduncle (right) on FA skeleton (from dMRI data)
12	25067	Mean FA in inferior cerebellar peduncle (left) on FA skeleton (from dMRI data)
13	25068	Mean FA in superior cerebellar peduncle (right) on FA skeleton (from dMRI data)
14	25069	Mean FA in superior cerebellar peduncle (left) on FA skeleton (from dMRI data)
15	25070	Mean FA in cerebral peduncle (right) on FA skeleton (from dMRI data)
16	25071	Mean FA in cerebral peduncle (left) on FA skeleton (from dMRI data)
17	25072	Mean FA in anterior limb of internal capsule (right) on FA skeleton (from dMRI data)
18	25073	Mean FA in anterior limb of internal capsule (left) on FA skeleton (from dMRI data)
19	25074	Mean FA in posterior limb of internal capsule (right) on FA skeleton (from dMRI data)
20	25075	Mean FA in posterior limb of internal capsule (left) on FA skeleton (from dMRI data)
21	25076	Mean FA in retrolenticular part of internal capsule (right) on FA skeleton (from dMRI data)
22	25077	Mean FA in retrolenticular part of internal capsule (left) on FA skeleton (from dMRI data)
23	25078	Mean FA in anterior corona radiata (right) on FA skeleton (from dMRI data)
24	25079	Mean FA in anterior corona radiata (left) on FA skeleton (from dMRI data)
25	25080	Mean FA in superior corona radiata (right) on FA skeleton (from dMRI data)
26	25081	Mean FA in superior corona radiata (left) on FA skeleton (from dMRI data)
27	25082	Mean FA in posterior corona radiata (right) on FA skeleton (from dMRI data)
28	25083	Mean FA in posterior corona radiata (left) on FA skeleton (from dMRI data)
29	25084	Mean FA in posterior thalamic radiation (right) on FA skeleton (from dMRI data)
30	25085	Mean FA in posterior thalamic radiation (left) on FA skeleton (from dMRI data)
31	25086	Mean FA in sagittal stratum (right) on FA skeleton (from dMRI data)
32	25087	Mean FA in sagittal stratum (left) on FA skeleton (from dMRI data)
33	25088	Mean FA in external capsule (right) on FA skeleton (from dMRI data)
34	25089	Mean FA in external capsule (left) on FA skeleton (from dMRI data)
35	25090	Mean FA in cingulum cingulate gyrus (right) on FA skeleton (from dMRI data)
36	25091	Mean FA in cingulum cingulate gyrus (left) on FA skeleton (from dMRI data)
37	25092	Mean FA in cingulum hippocampus (right) on FA skeleton (from dMRI data)
38	25093	Mean FA in cingulum hippocampus (left) on FA skeleton (from dMRI data)
39	25094	Mean FA in fornix cres+stria terminalis (right) on FA skeleton (from dMRI data)
40	25095	Mean FA in fornix cres+stria terminalis (left) on FA skeleton (from dMRI data)
41	25096	Mean FA in superior longitudinal fasciculus (right) on FA skeleton (from dMRI data)
42	25097	Mean FA in superior longitudinal fasciculus (left) on FA skeleton (from dMRI data)
43	25098	Mean FA in superior fronto-occipital fasciculus (right) on FA skeleton (from dMRI data)
44	25099	Mean FA in superior fronto-occipital fasciculus (left) on FA skeleton (from dMRI data)
45	25100	Mean FA in uncinate fasciculus (right) on FA skeleton (from dMRI data)
46	25101	Mean FA in uncinate fasciculus (left) on FA skeleton (from dMRI data)
47	25102	Mean FA in tapetum (right) on FA skeleton (from dMRI data)
48	25103	Mean FA in tapetum (left) on FA skeleton (from dMRI data)
49	25104	Mean MD in middle cerebellar peduncle on FA skeleton (from dMRI data)
50	25105	Mean MD in pontine crossing tract on FA skeleton (from dMRI data)
51	25106	Mean MD in genu of corpus callosum on FA skeleton (from dMRI data)
52	25107	Mean MD in body of corpus callosum on FA skeleton (from dMRI data)
53	25108	Mean MD in splenium of corpus callosum on FA skeleton (from dMRI data)
54	25109	Mean MD in fornix on FA skeleton (from dMRI data)
55	25110	Mean MD in corticospinal tract (right) on FA skeleton (from dMRI data)
56	25111	Mean MD in corticospinal tract (left) on FA skeleton (from dMRI data)
57	25112	Mean MD in medial lemniscus (right) on FA skeleton (from dMRI data)
58	25113	Mean MD in medial lemniscus (left) on FA skeleton (from dMRI data)
59	25114	Mean MD in inferior cerebellar peduncle (right) on FA skeleton (from dMRI data)
60	25115	Mean MD in inferior cerebellar peduncle (left) on FA skeleton (from dMRI data)
61	25116	Mean MD in superior cerebellar peduncle (right) on FA skeleton (from dMRI data)
62	25117	Mean MD in superior cerebellar peduncle (left) on FA skeleton (from dMRI data)
63	25118	Mean MD in cerebral peduncle (right) on FA skeleton (from dMRI data)
64	25119	Mean MD in cerebral peduncle (left) on FA skeleton (from dMRI data)
65	25120	Mean MD in anterior limb of internal capsule (right) on FA skeleton (from dMRI data)
66	25121	Mean MD in anterior limb of internal capsule (left) on FA skeleton (from dMRI data)
67	25122	Mean MD in posterior limb of internal capsule (right) on FA skeleton (from dMRI data)
68	25123	Mean MD in posterior limb of internal capsule (left) on FA skeleton (from dMRI data)
69	25124	Mean MD in retrolenticular part of internal capsule (right) on FA skeleton (from dMRI data)
70	25125	Mean MD in retrolenticular part of internal capsule (left) on FA skeleton (from dMRI data)
71	25126	Mean MD in anterior corona radiata (right) on FA skeleton (from dMRI data)
72	25127	Mean MD in anterior corona radiata (left) on FA skeleton (from dMRI data)
73	25128	Mean MD in superior corona radiata (right) on FA skeleton (from dMRI data)
74	25129	Mean MD in superior corona radiata (left) on FA skeleton (from dMRI data)
75	25130	Mean MD in posterior corona radiata (right) on FA skeleton (from dMRI data)
76	25131	Mean MD in posterior corona radiata (left) on FA skeleton (from dMRI data)
77	25132	Mean MD in posterior thalamic radiation (right) on FA skeleton (from dMRI data)
78	25133	Mean MD in posterior thalamic radiation (left) on FA skeleton (from dMRI data)
79	25134	Mean MD in sagittal stratum (right) on FA skeleton (from dMRI data)
80	25135	Mean MD in sagittal stratum (left) on FA skeleton (from dMRI data)
81	25136	Mean MD in external capsule (right) on FA skeleton (from dMRI data)
82	25137	Mean MD in external capsule (left) on FA skeleton (from dMRI data)
83	25138	Mean MD in cingulum cingulate gyrus (right) on FA skeleton (from dMRI data)
84	25139	Mean MD in cingulum cingulate gyrus (left) on FA skeleton (from dMRI data)

85	25140	Mean MD in cingulum hippocampus (right) on FA skeleton (from dMRI data)
86	25141	Mean MD in cingulum hippocampus (left) on FA skeleton (from dMRI data)
87	25142	Mean MD in fornix cres+stria terminalis (right) on FA skeleton (from dMRI data)
88	25143	Mean MD in fornix cres+stria terminalis (left) on FA skeleton (from dMRI data)
89	25144	Mean MD in superior longitudinal fasciculus (right) on FA skeleton (from dMRI data)
90	25145	Mean MD in superior longitudinal fasciculus (left) on FA skeleton (from dMRI data)
91	25146	Mean MD in superior fronto-occipital fasciculus (right) on FA skeleton (from dMRI data)
92	25147	Mean MD in superior fronto-occipital fasciculus (left) on FA skeleton (from dMRI data)
93	25148	Mean MD in uncinate fasciculus (right) on FA skeleton (from dMRI data)
94	25149	Mean MD in uncinate fasciculus (left) on FA skeleton (from dMRI data)
95	25150	Mean MD in tapetum (right) on FA skeleton (from dMRI data)
96	25151	Mean MD in tapetum (left) on FA skeleton (from dMRI data)
97	25152	Mean MO in middle cerebellar peduncle on FA skeleton (from dMRI data)
98	25153	Mean MO in pontine crossing tract on FA skeleton (from dMRI data)
99	25154	Mean MO in genu of corpus callosum on FA skeleton (from dMRI data)
100	25155	Mean MO in body of corpus callosum on FA skeleton (from dMRI data)
101	25156	Mean MO in splenium of corpus callosum on FA skeleton (from dMRI data)
102	25157	Mean MO in fornix on FA skeleton (from dMRI data)
103	25158	Mean MO in corticospinal tract (right) on FA skeleton (from dMRI data)
104	25159	Mean MO in corticospinal tract (left) on FA skeleton (from dMRI data)
105	25160	Mean MO in medial lemniscus (right) on FA skeleton (from dMRI data)
106	25161	Mean MO in medial lemniscus (left) on FA skeleton (from dMRI data)
107	25162	Mean MO in inferior cerebellar peduncle (right) on FA skeleton (from dMRI data)
108	25163	Mean MO in inferior cerebellar peduncle (left) on FA skeleton (from dMRI data)
109	25164	Mean MO in superior cerebellar peduncle (right) on FA skeleton (from dMRI data)
110	25165	Mean MO in superior cerebellar peduncle (left) on FA skeleton (from dMRI data)
111	25166	Mean MO in cerebral peduncle (right) on FA skeleton (from dMRI data)
112	25167	Mean MO in cerebral peduncle (left) on FA skeleton (from dMRI data)
113	25168	Mean MO in anterior limb of internal capsule (right) on FA skeleton (from dMRI data)
114	25169	Mean MO in anterior limb of internal capsule (left) on FA skeleton (from dMRI data)
115	25170	Mean MO in posterior limb of internal capsule (right) on FA skeleton (from dMRI data)
116	25171	Mean MO in posterior limb of internal capsule (left) on FA skeleton (from dMRI data)
117	25172	Mean MO in retrolenticular part of internal capsule (right) on FA skeleton (from dMRI data)
118	25173	Mean MO in retrolenticular part of internal capsule (left) on FA skeleton (from dMRI data)
119	25174	Mean MO in anterior corona radiata (right) on FA skeleton (from dMRI data)
120	25175	Mean MO in anterior corona radiata (left) on FA skeleton (from dMRI data)
121	25176	Mean MO in superior corona radiata (right) on FA skeleton (from dMRI data)
122	25177	Mean MO in superior corona radiata (left) on FA skeleton (from dMRI data)
123	25178	Mean MO in posterior corona radiata (right) on FA skeleton (from dMRI data)
124	25179	Mean MO in posterior corona radiata (left) on FA skeleton (from dMRI data)
125	25180	Mean MO in posterior thalamic radiation (right) on FA skeleton (from dMRI data)
126	25181	Mean MO in posterior thalamic radiation (left) on FA skeleton (from dMRI data)
127	25182	Mean MO in sagittal stratum (right) on FA skeleton (from dMRI data)
128	25183	Mean MO in sagittal stratum (left) on FA skeleton (from dMRI data)
129	25184	Mean MO in external capsule (right) on FA skeleton (from dMRI data)
130	25185	Mean MO in external capsule (left) on FA skeleton (from dMRI data)
131	25186	Mean MO in cingulum cingulate gyrus (right) on FA skeleton (from dMRI data)
132	25187	Mean MO in cingulum cingulate gyrus (left) on FA skeleton (from dMRI data)
133	25188	Mean MO in cingulum hippocampus (right) on FA skeleton (from dMRI data)
134	25189	Mean MO in cingulum hippocampus (left) on FA skeleton (from dMRI data)
135	25190	Mean MO in fornix cres+stria terminalis (right) on FA skeleton (from dMRI data)
136	25191	Mean MO in fornix cres+stria terminalis (left) on FA skeleton (from dMRI data)
137	25192	Mean MO in superior longitudinal fasciculus (right) on FA skeleton (from dMRI data)
138	25193	Mean MO in superior longitudinal fasciculus (left) on FA skeleton (from dMRI data)
139	25194	Mean MO in superior fronto-occipital fasciculus (right) on FA skeleton (from dMRI data)
140	25195	Mean MO in superior fronto-occipital fasciculus (left) on FA skeleton (from dMRI data)
141	25196	Mean MO in uncinate fasciculus (right) on FA skeleton (from dMRI data)
142	25197	Mean MO in uncinate fasciculus (left) on FA skeleton (from dMRI data)
143	25198	Mean MO in tapetum (right) on FA skeleton (from dMRI data)
144	25199	Mean MO in tapetum (left) on FA skeleton (from dMRI data)
145	25200	Mean L1 in middle cerebellar peduncle on FA skeleton (from dMRI data)
146	25201	Mean L1 in pontine crossing tract on FA skeleton (from dMRI data)
147	25202	Mean L1 in genu of corpus callosum on FA skeleton (from dMRI data)
148	25203	Mean L1 in body of corpus callosum on FA skeleton (from dMRI data)
149	25204	Mean L1 in splenium of corpus callosum on FA skeleton (from dMRI data)
150	25205	Mean L1 in fornix on FA skeleton (from dMRI data)
151	25206	Mean L1 in corticospinal tract (right) on FA skeleton (from dMRI data)
152	25207	Mean L1 in corticospinal tract (left) on FA skeleton (from dMRI data)
153	25208	Mean L1 in medial lemniscus (right) on FA skeleton (from dMRI data)
154	25209	Mean L1 in medial lemniscus (left) on FA skeleton (from dMRI data)
155	25210	Mean L1 in inferior cerebellar peduncle (right) on FA skeleton (from dMRI data)
156	25211	Mean L1 in inferior cerebellar peduncle (left) on FA skeleton (from dMRI data)
157	25212	Mean L1 in superior cerebellar peduncle (right) on FA skeleton (from dMRI data)
158	25213	Mean L1 in superior cerebellar peduncle (left) on FA skeleton (from dMRI data)
159	25214	Mean L1 in cerebral peduncle (right) on FA skeleton (from dMRI data)
160	25215	Mean L1 in cerebral peduncle (left) on FA skeleton (from dMRI data)
161	25216	Mean L1 in anterior limb of internal capsule (right) on FA skeleton (from dMRI data)
162	25217	Mean L1 in anterior limb of internal capsule (left) on FA skeleton (from dMRI data)
163	25218	Mean L1 in posterior limb of internal capsule (right) on FA skeleton (from dMRI data)
164	25219	Mean L1 in posterior limb of internal capsule (left) on FA skeleton (from dMRI data)
165	25220	Mean L1 in retrolenticular part of internal capsule (right) on FA skeleton (from dMRI data)
166	25221	Mean L1 in retrolenticular part of internal capsule (left) on FA skeleton (from dMRI data)
167	25222	Mean L1 in anterior corona radiata (right) on FA skeleton (from dMRI data)
168	25223	Mean L1 in anterior corona radiata (left) on FA skeleton (from dMRI data)
169	25224	Mean L1 in superior corona radiata (right) on FA skeleton (from dMRI data)
170	25225	Mean L1 in superior corona radiata (left) on FA skeleton (from dMRI data)
171	25226	Mean L1 in posterior corona radiata (right) on FA skeleton (from dMRI data)
172	25227	Mean L1 in posterior corona radiata (left) on FA skeleton (from dMRI data)
173	25228	Mean L1 in posterior thalamic radiation (right) on FA skeleton (from dMRI data)

174	25229	Mean L1 in posterior thalamic radiation (left) on FA skeleton (from dMRI data)
175	25230	Mean L1 in sagittal stratum (right) on FA skeleton (from dMRI data)
176	25231	Mean L1 in sagittal stratum (left) on FA skeleton (from dMRI data)
177	25232	Mean L1 in external capsule (right) on FA skeleton (from dMRI data)
178	25233	Mean L1 in external capsule (left) on FA skeleton (from dMRI data)
179	25234	Mean L1 in cingulum cingulate gyrus (right) on FA skeleton (from dMRI data)
180	25235	Mean L1 in cingulum cingulate gyrus (left) on FA skeleton (from dMRI data)
181	25236	Mean L1 in cingulum hippocampus (right) on FA skeleton (from dMRI data)
182	25237	Mean L1 in cingulum hippocampus (left) on FA skeleton (from dMRI data)
183	25238	Mean L1 in fornix cres+stria terminalis (right) on FA skeleton (from dMRI data)
184	25239	Mean L1 in fornix cres+stria terminalis (left) on FA skeleton (from dMRI data)
185	25240	Mean L1 in superior longitudinal fasciculus (right) on FA skeleton (from dMRI data)
186	25241	Mean L1 in superior longitudinal fasciculus (left) on FA skeleton (from dMRI data)
187	25242	Mean L1 in superior fronto-occipital fasciculus (right) on FA skeleton (from dMRI data)
188	25243	Mean L1 in superior fronto-occipital fasciculus (left) on FA skeleton (from dMRI data)
189	25244	Mean L1 in uncinate fasciculus (right) on FA skeleton (from dMRI data)
190	25245	Mean L1 in uncinate fasciculus (left) on FA skeleton (from dMRI data)
191	25246	Mean L1 in tapetum (right) on FA skeleton (from dMRI data)
192	25247	Mean L1 in tapetum (left) on FA skeleton (from dMRI data)
193	25248	Mean L2 in middle cerebellar peduncle on FA skeleton (from dMRI data)
194	25249	Mean L2 in pontine crossing tract on FA skeleton (from dMRI data)
195	25250	Mean L2 in genu of corpus callosum on FA skeleton (from dMRI data)
196	25251	Mean L2 in body of corpus callosum on FA skeleton (from dMRI data)
197	25252	Mean L2 in splenium of corpus callosum on FA skeleton (from dMRI data)
198	25253	Mean L2 in fornix on FA skeleton (from dMRI data)
199	25254	Mean L2 in corticospinal tract (right) on FA skeleton (from dMRI data)
200	25255	Mean L2 in corticospinal tract (left) on FA skeleton (from dMRI data)
201	25256	Mean L2 in medial lemniscus (right) on FA skeleton (from dMRI data)
202	25257	Mean L2 in medial lemniscus (left) on FA skeleton (from dMRI data)
203	25258	Mean L2 in inferior cerebellar peduncle (right) on FA skeleton (from dMRI data)
204	25259	Mean L2 in inferior cerebellar peduncle (left) on FA skeleton (from dMRI data)
205	25260	Mean L2 in superior cerebellar peduncle (right) on FA skeleton (from dMRI data)
206	25261	Mean L2 in superior cerebellar peduncle (left) on FA skeleton (from dMRI data)
207	25262	Mean L2 in cerebral peduncle (right) on FA skeleton (from dMRI data)
208	25263	Mean L2 in cerebral peduncle (left) on FA skeleton (from dMRI data)
209	25264	Mean L2 in anterior limb of internal capsule (right) on FA skeleton (from dMRI data)
210	25265	Mean L2 in anterior limb of internal capsule (left) on FA skeleton (from dMRI data)
211	25266	Mean L2 in posterior limb of internal capsule (right) on FA skeleton (from dMRI data)
212	25267	Mean L2 in posterior limb of internal capsule (left) on FA skeleton (from dMRI data)
213	25268	Mean L2 in retrolenticular part of internal capsule (right) on FA skeleton (from dMRI data)
214	25269	Mean L2 in retrolenticular part of internal capsule (left) on FA skeleton (from dMRI data)
215	25270	Mean L2 in anterior corona radiata (right) on FA skeleton (from dMRI data)
216	25271	Mean L2 in anterior corona radiata (left) on FA skeleton (from dMRI data)
217	25272	Mean L2 in superior corona radiata (right) on FA skeleton (from dMRI data)
218	25273	Mean L2 in superior corona radiata (left) on FA skeleton (from dMRI data)
219	25274	Mean L2 in posterior corona radiata (right) on FA skeleton (from dMRI data)
220	25275	Mean L2 in posterior corona radiata (left) on FA skeleton (from dMRI data)
221	25276	Mean L2 in posterior thalamic radiation (right) on FA skeleton (from dMRI data)
222	25277	Mean L2 in posterior thalamic radiation (left) on FA skeleton (from dMRI data)
223	25278	Mean L2 in sagittal stratum (right) on FA skeleton (from dMRI data)
224	25279	Mean L2 in sagittal stratum (left) on FA skeleton (from dMRI data)
225	25280	Mean L2 in external capsule (right) on FA skeleton (from dMRI data)
226	25281	Mean L2 in external capsule (left) on FA skeleton (from dMRI data)
227	25282	Mean L2 in cingulum cingulate gyrus (right) on FA skeleton (from dMRI data)
228	25283	Mean L2 in cingulum cingulate gyrus (left) on FA skeleton (from dMRI data)
229	25284	Mean L2 in cingulum hippocampus (right) on FA skeleton (from dMRI data)
230	25285	Mean L2 in cingulum hippocampus (left) on FA skeleton (from dMRI data)
231	25286	Mean L2 in fornix cres+stria terminalis (right) on FA skeleton (from dMRI data)
232	25287	Mean L2 in fornix cres+stria terminalis (left) on FA skeleton (from dMRI data)
233	25288	Mean L2 in superior longitudinal fasciculus (right) on FA skeleton (from dMRI data)
234	25289	Mean L2 in superior longitudinal fasciculus (left) on FA skeleton (from dMRI data)
235	25290	Mean L2 in superior fronto-occipital fasciculus (right) on FA skeleton (from dMRI data)
236	25291	Mean L2 in superior fronto-occipital fasciculus (left) on FA skeleton (from dMRI data)
237	25292	Mean L2 in uncinate fasciculus (right) on FA skeleton (from dMRI data)
238	25293	Mean L2 in uncinate fasciculus (left) on FA skeleton (from dMRI data)
239	25295	Mean L2 in tapetum (right) on FA skeleton (from dMRI data)
240	25294	Mean L2 in tapetum (left) on FA skeleton (from dMRI data)
241	25296	Mean L3 in middle cerebellar peduncle on FA skeleton (from dMRI data)
242	25297	Mean L3 in pontine crossing tract on FA skeleton (from dMRI data)
243	25298	Mean L3 in genu of corpus callosum on FA skeleton (from dMRI data)
244	25299	Mean L3 in body of corpus callosum on FA skeleton (from dMRI data)
245	25300	Mean L3 in splenium of corpus callosum on FA skeleton (from dMRI data)
246	25301	Mean L3 in fornix on FA skeleton (from dMRI data)
247	25302	Mean L3 in corticospinal tract (right) on FA skeleton (from dMRI data)
248	25303	Mean L3 in corticospinal tract (left) on FA skeleton (from dMRI data)
249	25304	Mean L3 in medial lemniscus (right) on FA skeleton (from dMRI data)
250	25305	Mean L3 in medial lemniscus (left) on FA skeleton (from dMRI data)
251	25306	Mean L3 in inferior cerebellar peduncle (right) on FA skeleton (from dMRI data)
252	25307	Mean L3 in inferior cerebellar peduncle (left) on FA skeleton (from dMRI data)
253	25308	Mean L3 in superior cerebellar peduncle (right) on FA skeleton (from dMRI data)
254	25309	Mean L3 in superior cerebellar peduncle (left) on FA skeleton (from dMRI data)
255	25310	Mean L3 in cerebral peduncle (right) on FA skeleton (from dMRI data)
256	25311	Mean L3 in cerebral peduncle (left) on FA skeleton (from dMRI data)
257	25312	Mean L3 in anterior limb of internal capsule (right) on FA skeleton (from dMRI data)
258	25313	Mean L3 in anterior limb of internal capsule (left) on FA skeleton (from dMRI data)
259	25314	Mean L3 in posterior limb of internal capsule (right) on FA skeleton (from dMRI data)
260	25315	Mean L3 in posterior limb of internal capsule (left) on FA skeleton (from dMRI data)
261	25316	Mean L3 in retrolenticular part of internal capsule (right) on FA skeleton (from dMRI data)
262	25317	Mean L3 in retrolenticular part of internal capsule (left) on FA skeleton (from dMRI data)

263	25318	Mean L3 in anterior corona radiata (right) on FA skeleton (from dMRI data)
264	25319	Mean L3 in anterior corona radiata (left) on FA skeleton (from dMRI data)
265	25320	Mean L3 in superior corona radiata (right) on FA skeleton (from dMRI data)
266	25321	Mean L3 in superior corona radiata (left) on FA skeleton (from dMRI data)
267	25322	Mean L3 in posterior corona radiata (right) on FA skeleton (from dMRI data)
268	25323	Mean L3 in posterior corona radiata (left) on FA skeleton (from dMRI data)
269	25324	Mean L3 in posterior thalamic radiation (right) on FA skeleton (from dMRI data)
270	25325	Mean L3 in posterior thalamic radiation (left) on FA skeleton (from dMRI data)
271	25326	Mean L3 in sagittal stratum (right) on FA skeleton (from dMRI data)
272	25327	Mean L3 in sagittal stratum (left) on FA skeleton (from dMRI data)
273	25328	Mean L3 in external capsule (right) on FA skeleton (from dMRI data)
274	25329	Mean L3 in external capsule (left) on FA skeleton (from dMRI data)
275	25330	Mean L3 in cingulum cingulate gyrus (right) on FA skeleton (from dMRI data)
276	25331	Mean L3 in cingulum cingulate gyrus (left) on FA skeleton (from dMRI data)
277	25332	Mean L3 in cingulum hippocampus (right) on FA skeleton (from dMRI data)
278	25333	Mean L3 in cingulum hippocampus (left) on FA skeleton (from dMRI data)
279	25334	Mean L3 in fornix cres+stria terminalis (right) on FA skeleton (from dMRI data)
280	25335	Mean L3 in fornix cres+stria terminalis (left) on FA skeleton (from dMRI data)
281	25336	Mean L3 in superior longitudinal fasciculus (right) on FA skeleton (from dMRI data)
282	25337	Mean L3 in superior longitudinal fasciculus (left) on FA skeleton (from dMRI data)
283	25338	Mean L3 in superior fronto-occipital fasciculus (right) on FA skeleton (from dMRI data)
284	25339	Mean L3 in superior fronto-occipital fasciculus (left) on FA skeleton (from dMRI data)
285	25340	Mean L3 in uncinate fasciculus (right) on FA skeleton (from dMRI data)
286	25341	Mean L3 in uncinate fasciculus (left) on FA skeleton (from dMRI data)
287	25342	Mean L3 in tapetum (right) on FA skeleton (from dMRI data)
288	25343	Mean L3 in tapetum (left) on FA skeleton (from dMRI data)
289	25344	Mean ICVF in middle cerebellar peduncle on FA skeleton (from dMRI data)
290	25345	Mean ICVF in pontine crossing tract on FA skeleton (from dMRI data)
291	25346	Mean ICVF in genu of corpus callosum on FA skeleton (from dMRI data)
292	25347	Mean ICVF in body of corpus callosum on FA skeleton (from dMRI data)
293	25348	Mean ICVF in splenium of corpus callosum on FA skeleton (from dMRI data)
294	25349	Mean ICVF in fornix on FA skeleton (from dMRI data)
295	25350	Mean ICVF in corticospinal tract (right) on FA skeleton (from dMRI data)
296	25351	Mean ICVF in corticospinal tract (left) on FA skeleton (from dMRI data)
297	25352	Mean ICVF in medial lemniscus (right) on FA skeleton (from dMRI data)
298	25353	Mean ICVF in medial lemniscus (left) on FA skeleton (from dMRI data)
299	25354	Mean ICVF in inferior cerebellar peduncle (right) on FA skeleton (from dMRI data)
300	25355	Mean ICVF in inferior cerebellar peduncle (left) on FA skeleton (from dMRI data)
301	25356	Mean ICVF in superior cerebellar peduncle (right) on FA skeleton (from dMRI data)
302	25357	Mean ICVF in superior cerebellar peduncle (left) on FA skeleton (from dMRI data)
303	25358	Mean ICVF in cerebral peduncle (right) on FA skeleton (from dMRI data)
304	25359	Mean ICVF in cerebral peduncle (left) on FA skeleton (from dMRI data)
305	25360	Mean ICVF in anterior limb of internal capsule (right) on FA skeleton (from dMRI data)
306	25361	Mean ICVF in anterior limb of internal capsule (left) on FA skeleton (from dMRI data)
307	25362	Mean ICVF in posterior limb of internal capsule (right) on FA skeleton (from dMRI data)
308	25363	Mean ICVF in posterior limb of internal capsule (left) on FA skeleton (from dMRI data)
309	25364	Mean ICVF in retrolenticular part of internal capsule (right) on FA skeleton (from dMRI data)
310	25365	Mean ICVF in retrolenticular part of internal capsule (left) on FA skeleton (from dMRI data)
311	25366	Mean ICVF in anterior corona radiata (right) on FA skeleton (from dMRI data)
312	25367	Mean ICVF in anterior corona radiata (left) on FA skeleton (from dMRI data)
313	25368	Mean ICVF in superior corona radiata (right) on FA skeleton (from dMRI data)
314	25369	Mean ICVF in superior corona radiata (left) on FA skeleton (from dMRI data)
315	25370	Mean ICVF in posterior corona radiata (right) on FA skeleton (from dMRI data)
316	25371	Mean ICVF in posterior corona radiata (left) on FA skeleton (from dMRI data)
317	25372	Mean ICVF in posterior thalamic radiation (right) on FA skeleton (from dMRI data)
318	25373	Mean ICVF in posterior thalamic radiation (left) on FA skeleton (from dMRI data)
319	25374	Mean ICVF in sagittal stratum (right) on FA skeleton (from dMRI data)
320	25375	Mean ICVF in sagittal stratum (left) on FA skeleton (from dMRI data)
321	25376	Mean ICVF in external capsule (right) on FA skeleton (from dMRI data)
322	25377	Mean ICVF in external capsule (left) on FA skeleton (from dMRI data)
323	25378	Mean ICVF in cingulum cingulate gyrus (right) on FA skeleton (from dMRI data)
324	25379	Mean ICVF in cingulum cingulate gyrus (left) on FA skeleton (from dMRI data)
325	25380	Mean ICVF in cingulum hippocampus (right) on FA skeleton (from dMRI data)
326	25381	Mean ICVF in cingulum hippocampus (left) on FA skeleton (from dMRI data)
327	25382	Mean ICVF in fornix cres+stria terminalis (right) on FA skeleton (from dMRI data)
328	25383	Mean ICVF in fornix cres+stria terminalis (left) on FA skeleton (from dMRI data)
329	25384	Mean ICVF in superior longitudinal fasciculus (right) on FA skeleton (from dMRI data)
330	25385	Mean ICVF in superior longitudinal fasciculus (left) on FA skeleton (from dMRI data)
331	25386	Mean ICVF in superior fronto-occipital fasciculus (right) on FA skeleton (from dMRI data)
332	25387	Mean ICVF in superior fronto-occipital fasciculus (left) on FA skeleton (from dMRI data)
333	25388	Mean ICVF in uncinate fasciculus (right) on FA skeleton (from dMRI data)
334	25389	Mean ICVF in uncinate fasciculus (left) on FA skeleton (from dMRI data)
335	25390	Mean ICVF in tapetum (right) on FA skeleton (from dMRI data)
336	25391	Mean ICVF in tapetum (left) on FA skeleton (from dMRI data)
337	25392	Mean OD in middle cerebellar peduncle on FA skeleton (from dMRI data)
338	25393	Mean OD in pontine crossing tract on FA skeleton (from dMRI data)
339	25394	Mean OD in genu of corpus callosum on FA skeleton (from dMRI data)
340	25395	Mean OD in body of corpus callosum on FA skeleton (from dMRI data)
341	25396	Mean OD in splenium of corpus callosum on FA skeleton (from dMRI data)
342	25397	Mean OD in fornix on FA skeleton (from dMRI data)
343	25398	Mean OD in corticospinal tract (right) on FA skeleton (from dMRI data)
344	25399	Mean OD in corticospinal tract (left) on FA skeleton (from dMRI data)
345	25400	Mean OD in medial lemniscus (right) on FA skeleton (from dMRI data)
346	25401	Mean OD in medial lemniscus (left) on FA skeleton (from dMRI data)
347	25402	Mean OD in inferior cerebellar peduncle (right) on FA skeleton (from dMRI data)
348	25403	Mean OD in inferior cerebellar peduncle (left) on FA skeleton (from dMRI data)
349	25404	Mean OD in superior cerebellar peduncle (right) on FA skeleton (from dMRI data)
350	25405	Mean OD in superior cerebellar peduncle (left) on FA skeleton (from dMRI data)
351	25406	Mean OD in cerebral peduncle (right) on FA skeleton (from dMRI data)

352	25407	Mean OD in cerebral peduncle (left) on FA skeleton (from dMRI data)
353	25408	Mean OD in anterior limb of internal capsule (right) on FA skeleton (from dMRI data)
354	25409	Mean OD in anterior limb of internal capsule (left) on FA skeleton (from dMRI data)
355	25410	Mean OD in posterior limb of internal capsule (right) on FA skeleton (from dMRI data)
356	25411	Mean OD in posterior limb of internal capsule (left) on FA skeleton (from dMRI data)
357	25412	Mean OD in retrolenticular part of internal capsule (right) on FA skeleton (from dMRI data)
358	25413	Mean OD in retrolenticular part of internal capsule (left) on FA skeleton (from dMRI data)
359	25414	Mean OD in anterior corona radiata (right) on FA skeleton (from dMRI data)
360	25415	Mean OD in anterior corona radiata (left) on FA skeleton (from dMRI data)
361	25416	Mean OD in superior corona radiata (right) on FA skeleton (from dMRI data)
362	25417	Mean OD in superior corona radiata (left) on FA skeleton (from dMRI data)
363	25418	Mean OD in posterior corona radiata (right) on FA skeleton (from dMRI data)
364	25419	Mean OD in posterior corona radiata (left) on FA skeleton (from dMRI data)
365	25420	Mean OD in posterior thalamic radiation (right) on FA skeleton (from dMRI data)
366	25421	Mean OD in posterior thalamic radiation (left) on FA skeleton (from dMRI data)
367	25422	Mean OD in sagittal stratum (right) on FA skeleton (from dMRI data)
368	25423	Mean OD in sagittal stratum (left) on FA skeleton (from dMRI data)
369	25424	Mean OD in external capsule (right) on FA skeleton (from dMRI data)
370	25425	Mean OD in external capsule (left) on FA skeleton (from dMRI data)
371	25426	Mean OD in cingulum cingulate gyrus (right) on FA skeleton (from dMRI data)
372	25427	Mean OD in cingulum cingulate gyrus (left) on FA skeleton (from dMRI data)
373	25428	Mean OD in cingulum hippocampus (right) on FA skeleton (from dMRI data)
374	25429	Mean OD in cingulum hippocampus (left) on FA skeleton (from dMRI data)
375	25430	Mean OD in fornix cres+stria terminalis (right) on FA skeleton (from dMRI data)
376	25431	Mean OD in fornix cres+stria terminalis (left) on FA skeleton (from dMRI data)
377	25432	Mean OD in superior longitudinal fasciculus (right) on FA skeleton (from dMRI data)
378	25433	Mean OD in superior longitudinal fasciculus (left) on FA skeleton (from dMRI data)
379	25434	Mean OD in superior fronto-occipital fasciculus (right) on FA skeleton (from dMRI data)
380	25435	Mean OD in superior fronto-occipital fasciculus (left) on FA skeleton (from dMRI data)
381	25436	Mean OD in uncinate fasciculus (right) on FA skeleton (from dMRI data)
382	25437	Mean OD in uncinate fasciculus (left) on FA skeleton (from dMRI data)
383	25438	Mean OD in tapetum (right) on FA skeleton (from dMRI data)
384	25439	Mean OD in tapetum (left) on FA skeleton (from dMRI data)
385	25440	Mean ISOVF in middle cerebellar peduncle on FA skeleton (from dMRI data)
386	25441	Mean ISOVF in pontine crossing tract on FA skeleton (from dMRI data)
387	25442	Mean ISOVF in genu of corpus callosum on FA skeleton (from dMRI data)
388	25443	Mean ISOVF in body of corpus callosum on FA skeleton (from dMRI data)
389	25444	Mean ISOVF in splenium of corpus callosum on FA skeleton (from dMRI data)
390	25445	Mean ISOVF in fornix on FA skeleton (from dMRI data)
391	25446	Mean ISOVF in corticospinal tract (right) on FA skeleton (from dMRI data)
392	25447	Mean ISOVF in corticospinal tract (left) on FA skeleton (from dMRI data)
393	25448	Mean ISOVF in medial lemniscus (right) on FA skeleton (from dMRI data)
394	25449	Mean ISOVF in medial lemniscus (left) on FA skeleton (from dMRI data)
395	25450	Mean ISOVF in inferior cerebellar peduncle (right) on FA skeleton (from dMRI data)
396	25451	Mean ISOVF in inferior cerebellar peduncle (left) on FA skeleton (from dMRI data)
397	25452	Mean ISOVF in superior cerebellar peduncle (right) on FA skeleton (from dMRI data)
398	25453	Mean ISOVF in superior cerebellar peduncle (left) on FA skeleton (from dMRI data)
399	25454	Mean ISOVF in cerebral peduncle (right) on FA skeleton (from dMRI data)
400	25455	Mean ISOVF in cerebral peduncle (left) on FA skeleton (from dMRI data)
401	25456	Mean ISOVF in anterior limb of internal capsule (right) on FA skeleton (from dMRI data)
402	25457	Mean ISOVF in anterior limb of internal capsule (left) on FA skeleton (from dMRI data)
403	25458	Mean ISOVF in posterior limb of internal capsule (right) on FA skeleton (from dMRI data)
404	25459	Mean ISOVF in posterior limb of internal capsule (left) on FA skeleton (from dMRI data)
405	25460	Mean ISOVF in retrolenticular part of internal capsule (right) on FA skeleton (from dMRI data)
406	25461	Mean ISOVF in retrolenticular part of internal capsule (left) on FA skeleton (from dMRI data)
407	25462	Mean ISOVF in anterior corona radiata (right) on FA skeleton (from dMRI data)
408	25463	Mean ISOVF in anterior corona radiata (left) on FA skeleton (from dMRI data)
409	25464	Mean ISOVF in superior corona radiata (right) on FA skeleton (from dMRI data)
410	25465	Mean ISOVF in superior corona radiata (left) on FA skeleton (from dMRI data)
411	25466	Mean ISOVF in posterior corona radiata (right) on FA skeleton (from dMRI data)
412	25467	Mean ISOVF in posterior corona radiata (left) on FA skeleton (from dMRI data)
413	25468	Mean ISOVF in posterior thalamic radiation (right) on FA skeleton (from dMRI data)
414	25469	Mean ISOVF in posterior thalamic radiation (left) on FA skeleton (from dMRI data)
415	25470	Mean ISOVF in sagittal stratum (right) on FA skeleton (from dMRI data)
416	25471	Mean ISOVF in sagittal stratum (left) on FA skeleton (from dMRI data)
417	25472	Mean ISOVF in external capsule (right) on FA skeleton (from dMRI data)
418	25473	Mean ISOVF in external capsule (left) on FA skeleton (from dMRI data)
419	25474	Mean ISOVF in cingulum cingulate gyrus (right) on FA skeleton (from dMRI data)
420	25475	Mean ISOVF in cingulum cingulate gyrus (left) on FA skeleton (from dMRI data)
421	25476	Mean ISOVF in cingulum hippocampus (right) on FA skeleton (from dMRI data)
422	25477	Mean ISOVF in cingulum hippocampus (left) on FA skeleton (from dMRI data)
423	25478	Mean ISOVF in fornix cres+stria terminalis (right) on FA skeleton (from dMRI data)
424	25479	Mean ISOVF in fornix cres+stria terminalis (left) on FA skeleton (from dMRI data)
425	25480	Mean ISOVF in superior longitudinal fasciculus (right) on FA skeleton (from dMRI data)
426	25481	Mean ISOVF in superior longitudinal fasciculus (left) on FA skeleton (from dMRI data)
427	25482	Mean ISOVF in superior fronto-occipital fasciculus (right) on FA skeleton (from dMRI data)
428	25483	Mean ISOVF in superior fronto-occipital fasciculus (left) on FA skeleton (from dMRI data)
429	25484	Mean ISOVF in uncinate fasciculus (right) on FA skeleton (from dMRI data)
430	25485	Mean ISOVF in uncinate fasciculus (left) on FA skeleton (from dMRI data)
431	25486	Mean ISOVF in tapetum (right) on FA skeleton (from dMRI data)
432	25487	Mean ISOVF in tapetum (left) on FA skeleton (from dMRI data)
433	25007	Volume of white matter (from T1 brain image, normalised for head size)
434	25008	Volume of white matter (from T1 brain image)
435	25781	Total volume of white matter hyperintensities (from T1 and T2_FLAIR images)
436	26506	Mean intensity of WM-hypointensities in the whole brain generated by subcortical volumetric segmentation (aseg)
437	26528	Volume of WM-hypointensities in the whole brain generated by subcortical volumetric segmentation (aseg)
438	26540	Mean intensity of Cerebellum-White-Matter in the left hemisphere generated by subcortical volumetric segmentation (aseg)
439	26553	Volume of CerebralWhiteMatter in the left hemisphere generated by subcortical volumetric segmentation (aseg)
440	26556	Volume of Cerebellum-White-Matter in the left hemisphere generated by subcortical volumetric segmentation (aseg)

441	26571	Mean intensity of Cerebellum-White-Matter in the right hemisphere generated by subcortical volumetric segmentation (aseg)
442	26584	Volume of CerebralWhiteMatter in the right hemisphere generated by subcortical volumetric segmentation (aseg)
443	26587	Volume of Cerebellum-White-Matter in the right hemisphere generated by subcortical volumetric segmentation (aseg)

Supplementary Table 5. List of 46 imaging confounds used for de-confounding of the imaging-derived phenotypes.

Data-Field	Variable Name	Original Variable	Squard Variable	Original Variable (Gaussianised)	Squard Variable (Gaussianised)	Total Number of Variables
25741	rfMRI head motion	1	1	1	---	3
25742	tfMRI head motion	1	1	1	---	3
25000	Head size scaling	1	---	1	---	2
25756 - 25759	Bed position in scanner (x, y, z, table)	4	4	4	4	16
53	Date-related drift	10	---	10	---	20
54	Imaging Centre	1	---	---	---	1
31	Sex	1	---	---	---	1

Supplementary Table 6. List of the cognitive variables used to study the effect of COVID-19 and its pandemic.

**Data-Field**	**Cognitive Variable**	**Main Test**
398	Number of correct matches in round	Pairs matching (online)
399	Number of incorrect matches in round	Pairs matching
400	Time to complete round	Pairs matching
4282	Maximum digits remembered correctly	Numeric memory
6348	Duration to complete numeric path (trail #1)	Trail making (online)
6350	Duration to complete alphanumeric path (trail #2)	Trail making
6351	Total errors traversing alphanumeric path (trail #2)	Trail making
20016	Fluid intelligence score	Fluid intelligence (online)
20023	Mean time to correctly identify matches	Reaction time
23324	Number of symbol digit matches made correctly	Symbol digit substitution (online)

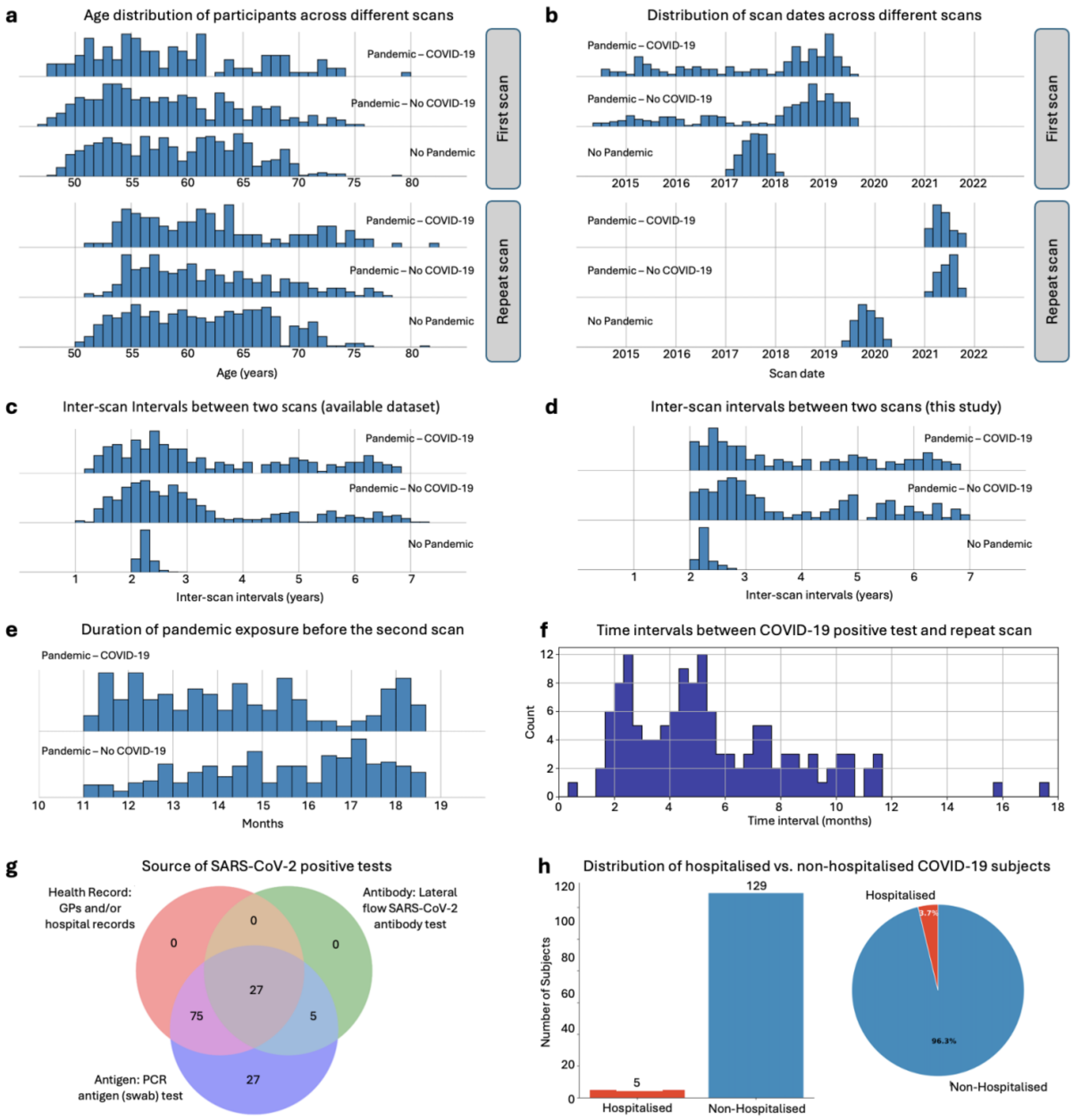

Supplementary Fig. 1. **Demographic and COVID-19-related characteristics of study participants.** a) Age distribution of participants at Timepoint 1 (top) and Timepoint 2 (bottom) for the Pandemic–COVID-19, Pandemic–No COVID-19, and No Pandemic groups. b) Distribution of brain imaging scan dates for Timepoint 1 (top) and Timepoint 2 (bottom) across different groups, illustrating the timing of brain scans. c) Distribution of inter-scan intervals (time between the first and second imaging scans) for the available dataset. d) Distribution of inter-scan intervals for the subset of participants included in the current study. e) Length of time between the onset of the COVID-19 pandemic and each participant's second brain imaging scan, reflecting the duration of pandemic exposure before follow-up. f) Time interval (in months) between the first confirmed positive COVID-19 test and the date of the repeat brain imaging scan. g) Source of evidence for COVID-19 infection within the COVID-19 group. "Antibody" refers to home-based lateral flow SARS-CoV-2 antibody tests; "Antigen" refers to PCR antigen (swab) tests; "Health records" indicate data from general practitioners (GPs) and/or hospital records. h) Distribution of hospitalised and non-hospitalised participants within the Pandemic–COVID-19 group.

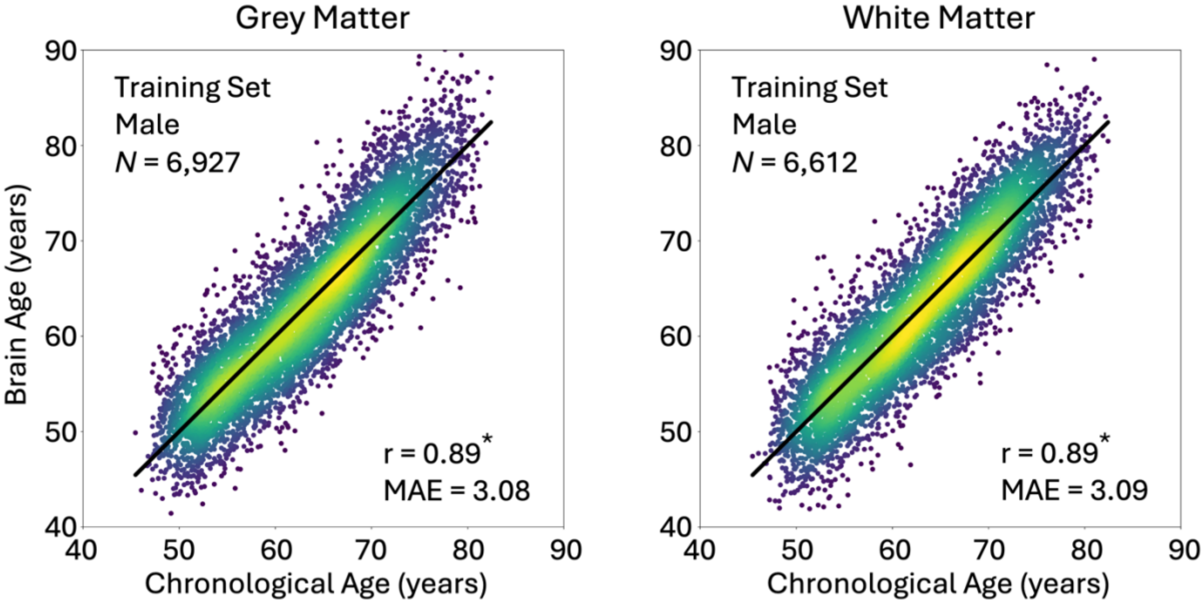

Supplementary Fig. 2. Predicted vs. chronological brain age in males using GM and WM models. The scatter plots show the relationship between chronological age (x-axis) and predicted brain age (y-axis) for GM and WM models, specifically for males.

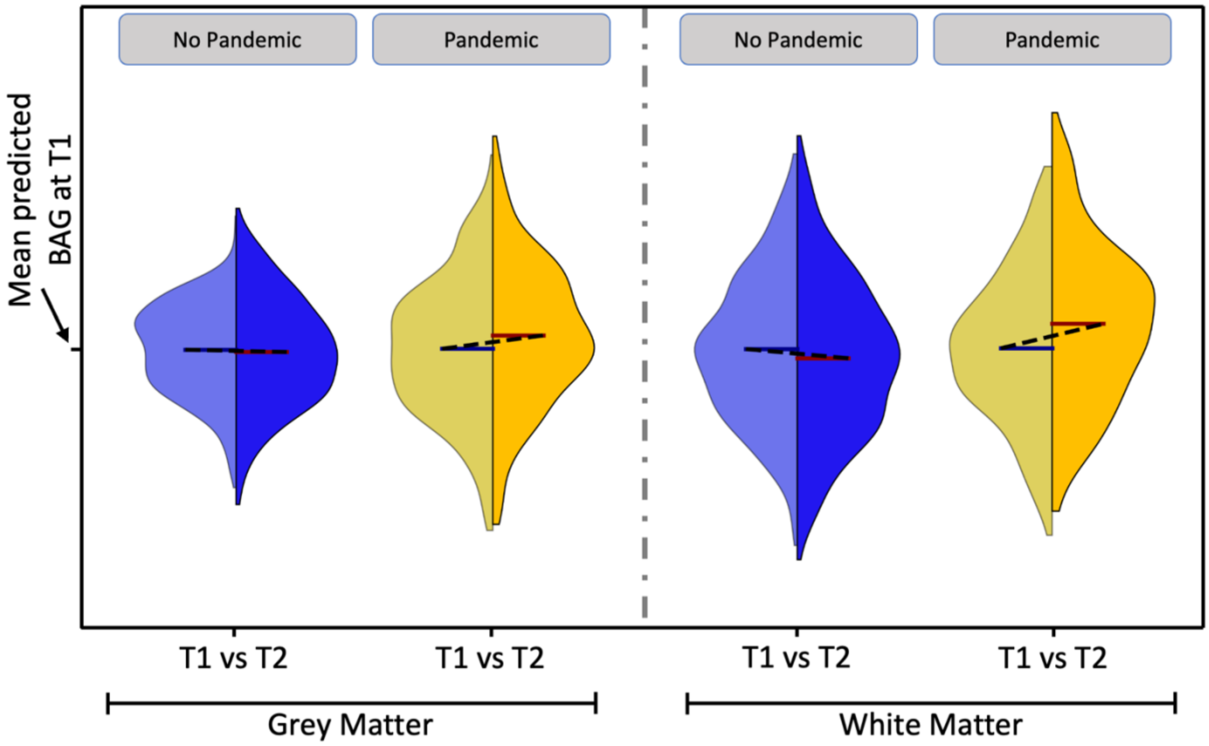

Supplementary Fig. 3. Longitudinal changes in brain age gap across timepoints in Pandemic and No Pandemic groups. Distribution of predicted brain age gaps (BAG) at Timepoint 1 (T1) and Timepoint 2 (T2) in both GM and WM models for the Pandemic and No Pandemic groups. Blue lines indicate the mean BAG at T1, while red lines indicate the mean at T2. Dashed lines connect the mean BAG values between T1 and T2, illustrating the longitudinal change in brain ageing across timepoints.

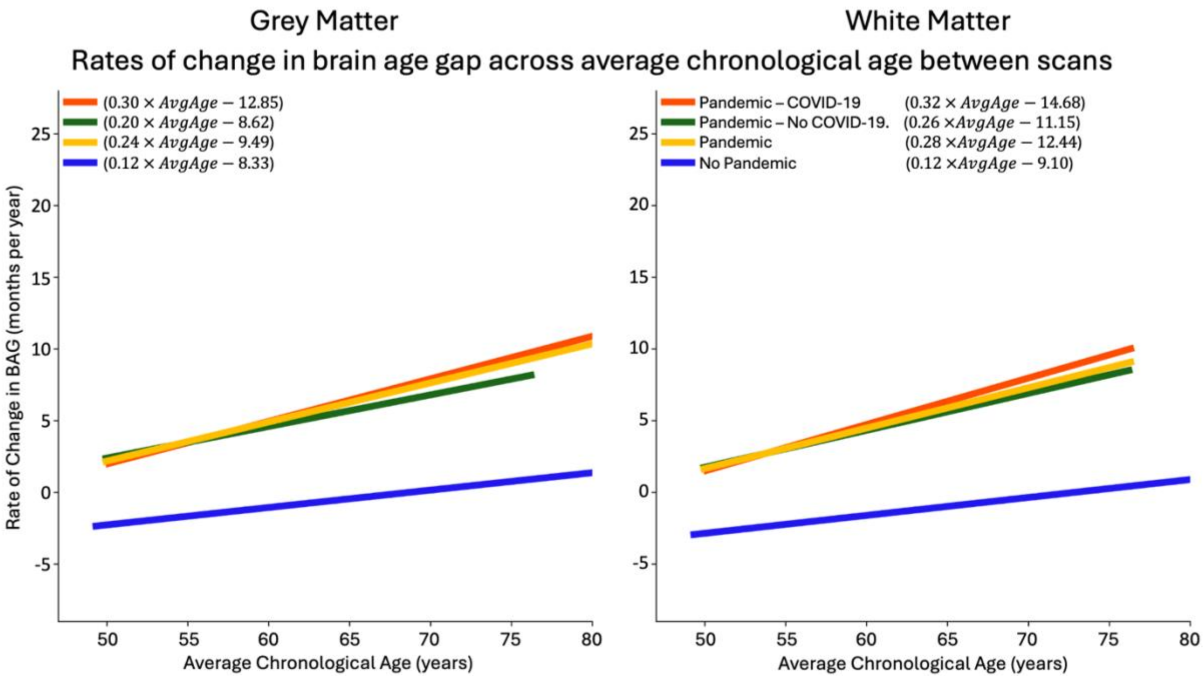

Supplementary Fig. 4. Association between brain age acceleration and chronological age in Pandemic and COVID-19 groups. Accelerated brain ageing observed in individuals with SARS-CoV-2 infection and those who experienced COVID-19 pandemic in both GM (left) and WM (right) models. The relationship between the rate of change in brain age gap (x-axis) and average chronological age between two scans (y-axis). The solid lines represent the best-fitted associations between the x-axis and y-axis variables for different participant groups.

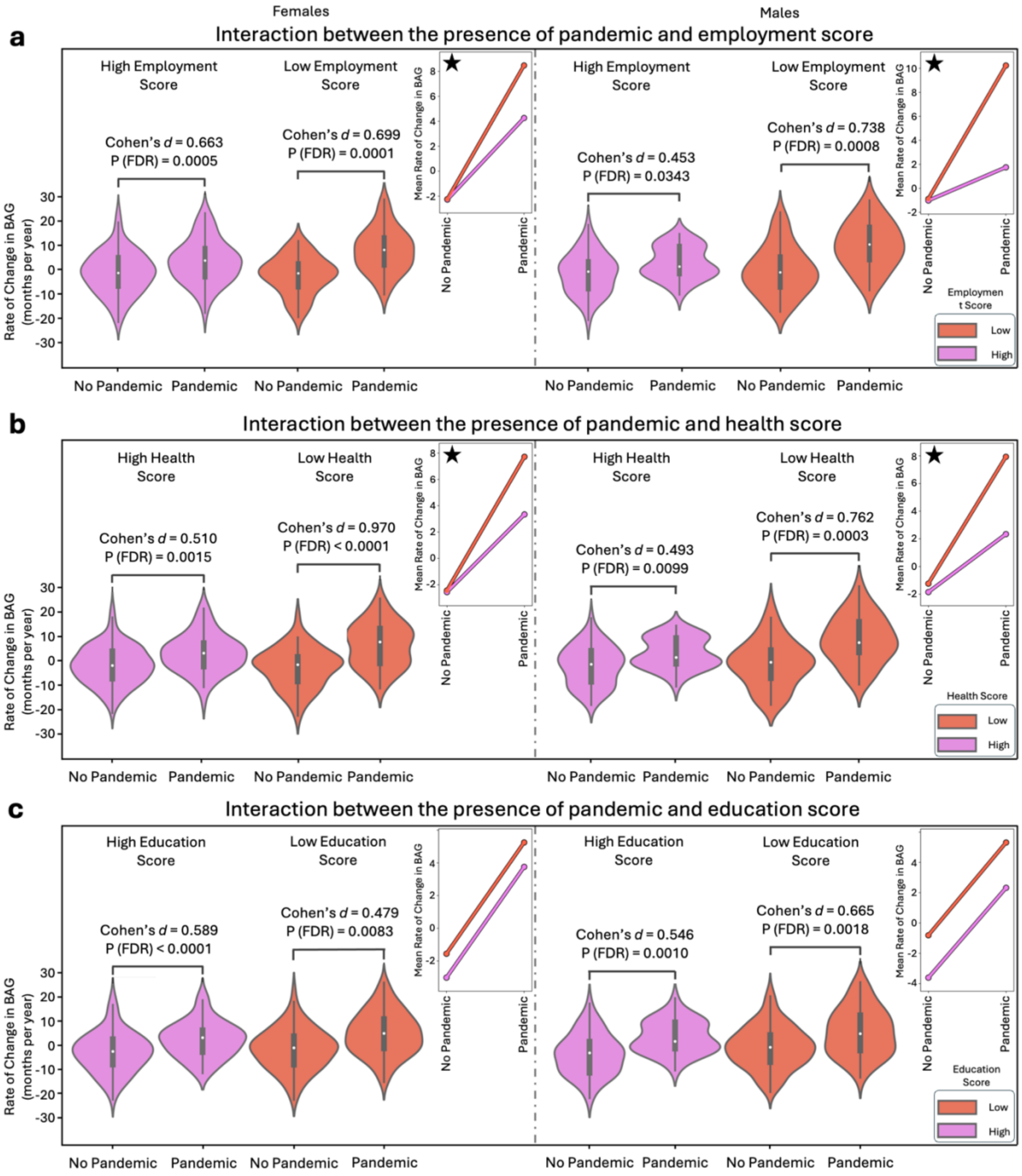

Supplementary Fig. 5. Sex-specific effects of deprivation and pandemic status on grey matter brain ageing. Interaction of deprivation indices and pandemic status in grey matter models for female and male participants. The figure illustrates the distribution of the rate of change in brain age gap (BAG) for the Pandemic and No Pandemic groups within the grey matter (GM) model, analysed separately for female and male participants. The analysis is presented for three deprivation indices: (a) Employment score, comparing participants with low (purple) vs. high (red) levels. (b) Health score, comparing participants with low (purple) vs. high (red) levels. (c) Education score, comparing participants with low (purple) vs. high (red) levels. Each subplot consists of two panels: the left panel represent female participants, while the right panel represents male participants. The results indicate significant differences in brain ageing patterns between the Pandemic and No Pandemic groups across all deprivation indices in both females and males within the GM model. Cohen's *d* and FDR-corrected p-values are annotated within the figure. A 2-factor, 2-level permutation test reveals significant

differences between low and high deprivation groups; Employment score: females (p(FDR) = 0.0090), males (p(FDR) = 0.0010); Health score: females (p(FDR) = 0.0206), males (p(FDR) = 0.0050); Education score: significant in males only (p(FDR) = 0.0066). Furthermore, significant interactions between pandemic status and deprivation factors were found; Employment score: females (p(FDR) = 0.0370), males (p(FDR) = 0.0010); Health score: females (p(FDR) = 0.0264), males (p(FDR) = 0.0210). All p-values fall within the 95% confidence interval (CI) [0.0443–0.0564].

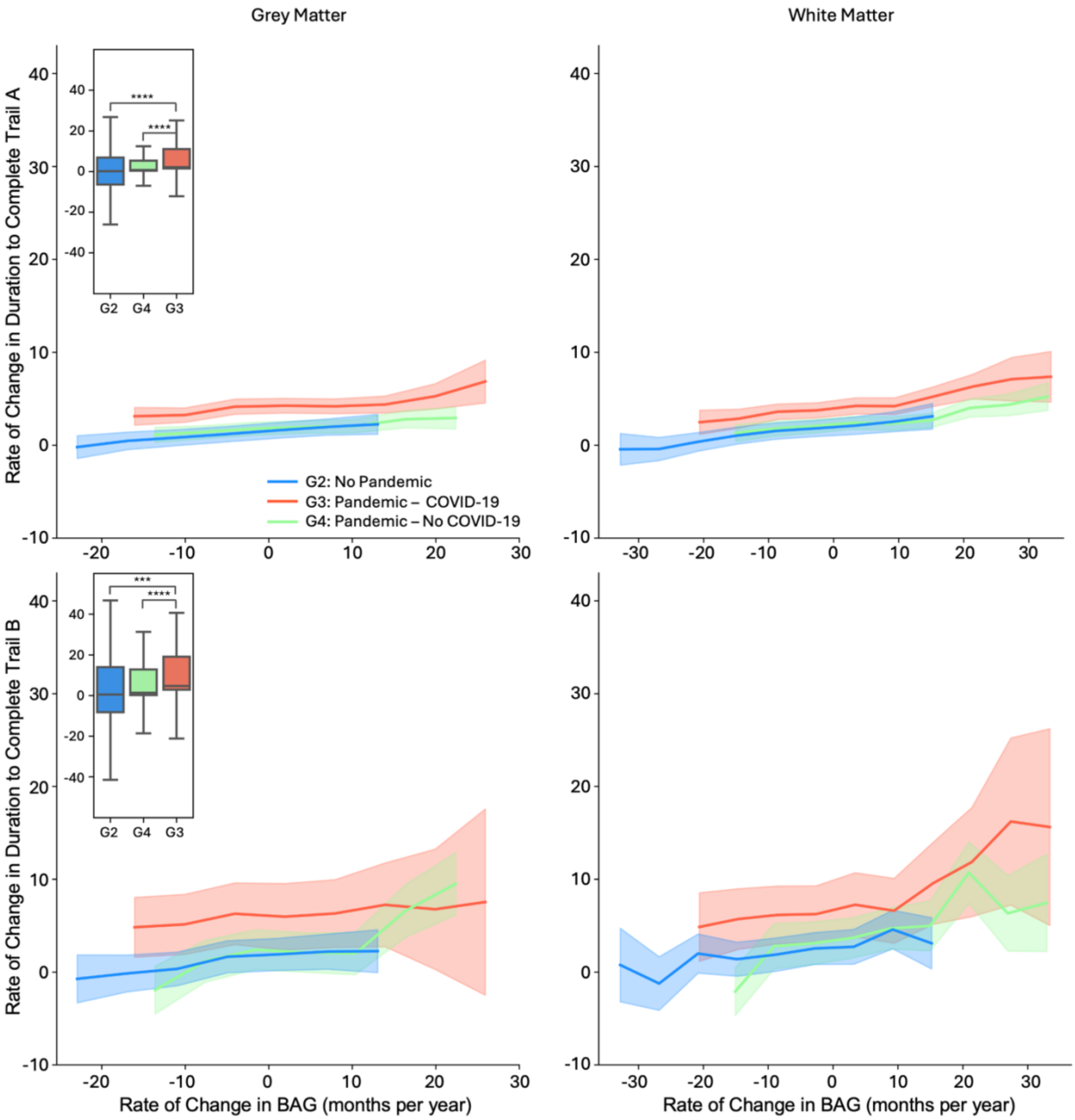

Supplementary Fig. 6. Impact of inter-scan interval (ISI) adjustment on cognitive performance changes. The figure illustrates the rate of change in duration to complete the (top) TMT-A and (bottom) TMT-B over two imaging scans across various rates of change in brain age gap, using an alternative normalisation method that accounts for inter-scan interval (ISI) differences. Results are shown for participants in the Pandemic – COVID-19 (G3, red), Pandemic – No COVID-19 (G4, green), and No Pandemic (blue, blue) groups, using both GM (left) and WM (right) models. A three-year sliding window was applied to create these curves. The boxplots in the top left of each plot represent the distribution of percentage change in TMT completion time across groups without a sliding window. Asterisks indicate the significance levels (*** signifies FDR-corrected p-values ≤ 0.001 and **** signifies FDR-corrected p-values ≤ 0.0001) calculated between different groups using two-sample t-tests.

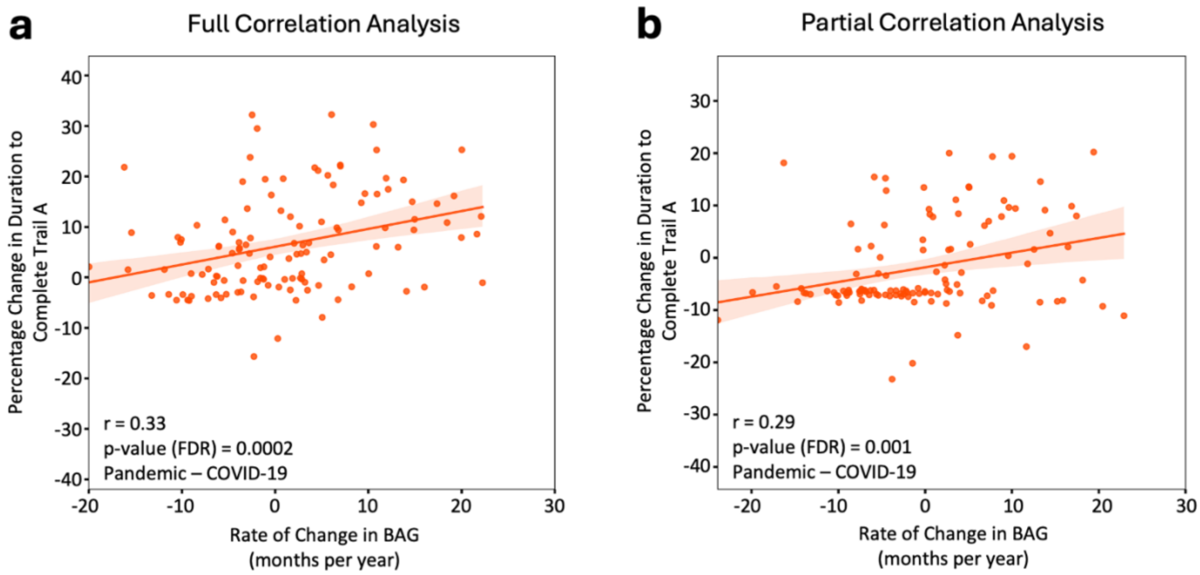

Supplementary Fig. 7. The association between the rate of change in brain age gap (BAG) and cognitive performance in the Pandemic-COVID-19 group. Regression lines indicate a significant correlation between the rate of change in BAG and the percentage change in completion time for the Trail Making Test Part A (TMT-A) across different scans. (a) Full correlation analysis, and (b) Partial correlation analysis with the effect of age regressed out.

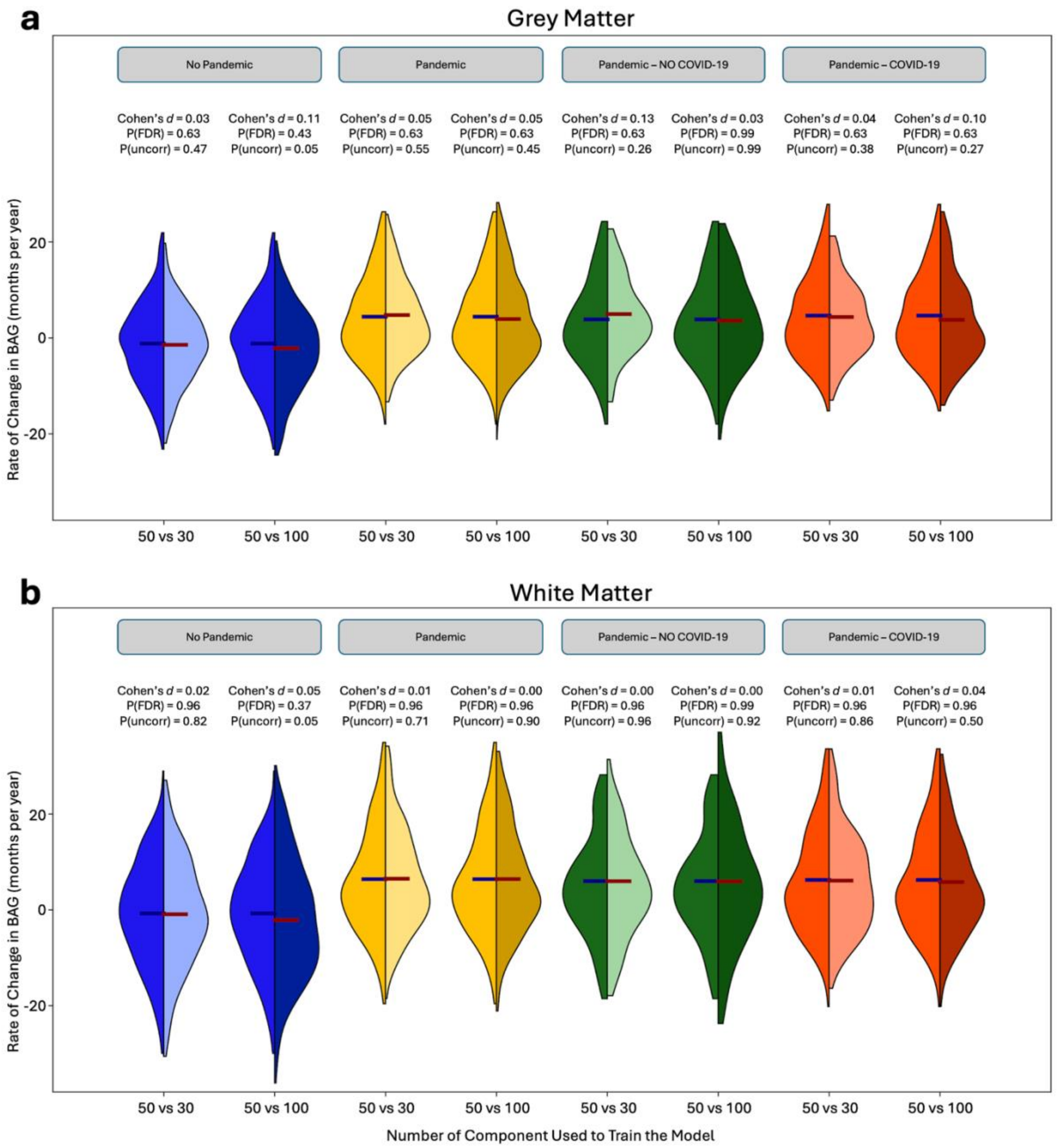

Supplementary Fig. 8. Effect of the number of retained principal components on the rate of change in brain age gap across different groups. Results are shown for (a) grey matter and (b) white matter models, comparing BAG rate of change when using 30, 50 (main model), and 100 principal components.

REFERENCES

1. Tian, Y. E. *et al.* Heterogeneous aging across multiple organ systems and prediction of chronic disease and mortality. *Nat Med* **29**, 1221–1231 (2023).
2. Massen, G. M. *et al.* Classifying the unclassifiable-a Delphi study to reach consensus on the fibrotic nature of diseases. *QJM* **116**, 429–435 (2023).
3. Smith, S. M., Vidaurre, D., Alfaro-Almagro, F., Nichols, T. E. & Miller, K. L. Estimation of brain age delta from brain imaging. *Neuroimage* **200**, 528–539 (2019).
