# Supplementary material for: Accelerated brain ageing during the COVID-19 pandemic
Source: Nat Commun. 2025 Jul 22;16:6411. doi: 10.1038/s41467-025-61033-4 (PMC12284169; doi:10.1038/s41467-025-61033-4)
Supplement: Supplementary file 2 — Reporting Summary [file 41467_2025_61033_MOESM2_ESM.pdf]

nature portfolio

Corresponding author(s): Stamatios N. Sotiropoulos
Dorothee P. Auer

Last updated by author(s): 02/06/2025

Reporting Summary

Nature Portfolio wishes to improve the reproducibility of the work that we publish. This form provides structure for consistency and transparency in reporting. For further information on Nature Portfolio policies, see our Editorial Policies and the Editorial Policy Checklist.

Statistics

For all statistical analyses, confirm that the following items are present in the figure legend, table legend, main text, or Methods section.

n/a	Confirmed	
☐	☒	The exact sample size (*n*) for each experimental group/condition, given as a discrete number and unit of measurement
☐	☒	A statement on whether measurements were taken from distinct samples or whether the same sample was measured repeatedly
☐	☒	The statistical test(s) used AND whether they are one- or two-sided<br>*Only common tests should be described solely by name; describe more complex techniques in the Methods section.*
☐	☒	A description of all covariates tested
☐	☒	A description of any assumptions or corrections, such as tests of normality and adjustment for multiple comparisons
☐	☒	A full description of the statistical parameters including central tendency (e.g. means) or other basic estimates (e.g. regression coefficient) AND variation (e.g. standard deviation) or associated estimates of uncertainty (e.g. confidence intervals)
☐	☒	For null hypothesis testing, the test statistic (e.g. F, t, r) with confidence intervals, effect sizes, degrees of freedom and P value noted<br>*Give P values as exact values whenever suitable.*
☒	☐	For Bayesian analysis, information on the choice of priors and Markov chain Monte Carlo settings
☒	☐	For hierarchical and complex designs, identification of the appropriate level for tests and full reporting of outcomes
☐	☒	Estimates of effect sizes (e.g. Cohen's *d*, Pearson's *r*), indicating how they were calculated

Our web collection on statistics for biologists contains articles on many of the points above.

Software and code

Policy information about availability of computer code

Data collection	Data were obtained from UK Biobank (available from UK Biobank upon data access application)
Data analysis	Python (v3.10) code for conducting the core analyses is available on GitHub (https://github.com/SPMIC-UoN/BrainAge_COVID-19).

For manuscripts utilizing custom algorithms or software that are central to the research but not yet described in published literature, software must be made available to editors and reviewers. We strongly encourage code deposition in a community repository (e.g. GitHub). See the Nature Portfolio guidelines for submitting code & software for further information.

Data

Policy information about availability of data

All manuscripts must include a data availability statement. This statement should provide the following information, where applicable:

- Accession codes, unique identifiers, or web links for publicly available datasets
- A description of any restrictions on data availability
- For clinical datasets or third party data, please ensure that the statement adheres to our policy

This project corresponds to UK Biobank application ID 43822. All source data is publicly available (upon data access application) from UK Biobank (https://biobank.ndph.ox.ac.uk/).

Research involving human participants, their data, or biological material

Policy information about studies with human participants or human data. See also policy information about sex, gender (identity/presentation), and sexual orientation and race, ethnicity and racism.

Reporting on sex and gender	The term sex was consistently used in this study to indicate biological attribute. Sex information for participant was based on self-report. Predictive models of biological age were established for males and females separately. Brain phenotypes were available for 16,330 individuals 8,959 females, 7,371 males). Sex was included in statistical analyses as covariate where applicable.
Reporting on race, ethnicity, or other socially relevant groupings	No socially relevant categorization variables were included in the present study.
Population characteristics	We selected participants from the UK (United Kingdom) Biobank imaging study, which provides multi-modal brain imaging data from over 42,677 individuals (released in April 2023). The first brain imaging scans began in 2014, with follow-up scans conducted from 2019 onwards. Detailed population characteristics of the cohort of the cohort of the present are provided in Supplementary Table S2.:<br>Total number: 16,330 (8,959 females)<br>Age range: 45.1 - 82.4 (mean: 62.4, std: 7.4)<br>Sex:8,959 females<br>Groups: 15,334 healthy with single brain scan for training of the predictive models. Pandemic-COVID-19: 134 participants, Pandemic-No COVID-19: 298 participants, No Pandemic: 564 participants.
Recruitment	Participants were recruited by the UK Biobank.
Ethics oversight	The UKBB has approval from the North West Multi-Centre Research Ethics Committee (MREC) to obtain and disseminate data and samples from the participants (http://www.ukbiobank.ac.uk/ethics/), and these ethical regulations cover the work in this study. Written informed consent was obtained from all participants.

Note that full information on the approval of the study protocol must also be provided in the manuscript.

Field-specific reporting

Please select the one below that is the best fit for your research. If you are not sure, read the appropriate sections before making your selection.

☒ Life sciences ☐ Behavioural & social sciences ☐ Ecological, evolutionary & environmental sciences

For a reference copy of the document with all sections, see nature.com/documents/nr-reporting-summary-flat.pdf

Life sciences study design

All studies must disclose on these points even when the disclosure is negative.

Sample size	Sample size was determined by data availability from UK Biobank.
Data exclusions	To minimise confounding factors in brain age predictions, we excluded participants with chronic disorders such as dementia, diabetes, heart and kidney disease, depression (see full list in Suppl. Table S1), before both their first and second scans. This ensured a focus on healthy individuals and reduced potential biases associated with disease-related brain changes. Additionally, participants with low-quality anatomical MRI data or unreliable brain imaging-derived phenotypes (IDPs) were removed. Technical outliers—defined as IDP values exceeding five standard deviations from the cohort mean—and participants with substantial missing or unreliable IDPs in any session were also excluded.
Replication	We trained the models in one-sub-cohort and tested them in a different (unseen) sub-cohort.
Randomization	N/A
Blinding	N/A

Reporting for specific materials, systems and methods

We require information from authors about some types of materials, experimental systems and methods used in many studies. Here, indicate whether each material, system or method listed is relevant to your study. If you are not sure if a list item applies to your research, read the appropriate section before selecting a response.

Materials & experimental systems

n/a	Involved in the study
☒	☐ Antibodies
☒	☐ Eukaryotic cell lines
☒	☐ Palaeontology and archaeology
☒	☐ Animals and other organisms
☒	☐ Clinical data
☒	☐ Dual use research of concern
☒	☐ Plants

Methods

n/a	Involved in the study
☒	☐ ChIP-seq
☒	☐ Flow cytometry
☐	☒ MRI-based neuroimaging

Plants

Seed stocks

Report on the source of all seed stocks or other plant material used. If applicable, state the seed stock centre and catalogue number. If plant specimens were collected from the field, describe the collection location, date and sampling procedures.

Novel plant genotypes

Describe the methods by which all novel plant genotypes were produced. This includes those generated by transgenic approaches, gene editing, chemical/radiation-based mutagenesis and hybridization. For transgenic lines, describe the transformation method, the number of independent lines analyzed and the generation upon which experiments were performed. For gene-edited lines, describe the editor used, the endogenous sequence targeted for editing, the targeting guide RNA sequence (if applicable) and how the editor was applied.

Authentication

Describe any authentication procedures for each seed stock used or novel genotype generated. Describe any experiments used to assess the effect of a mutation and, where applicable, how potential secondary effects (e.g. second site T-DNA insertions, mosiacism, off-target gene editing) were examined.

Magnetic resonance imaging

Experimental design

Design type

Structural and diffusion MRI

Design specifications

Design and data collection were coordinated by UK Biobank (K. Miller et al. Nature Neuroscience 2016)

Behavioral performance measures

No behavioral performance conducted during the scan, not applicable.

Acquisition

Imaging type(s)

T1-weighted, T2 weighted, and diffusion MRI

Field strength

3T

Sequence & imaging parameters

Imaging acquisition details were described by K. Miller et al. Nature Neuroscience 2016.

Area of acquisition

Whole Brain

Diffusion MRI ☒ Used ☐ Not used

Parameters

Diffusion data are acquired with two b-values (b=1,000 and 2,000 s/mm2) at 2-mm spatial resolution, with multiband acceleration factor of 3 (three slices are acquired simultaneously instead of just one). For each diffusion-weighted shell, 50 distinct diffusion encoding directions were acquired (covering 100 distinct directions over the two b-values). (No cardiac gating.)

Preprocessing

Preprocessing software

FSL 5.0.9, Freesurfer 6 and Freesurfer 7 for subcortical segmentation.

Normalization

Spatial normalisation was performed whenever applicable as described in detail by K. Miller et al. Nature Neuroscience 2016 and Alfaro-Almagro et al. NeuroImage 2018. Non-linear registration performed for diffusion MRI brain images based on T1w structural images.

Normalization template

MNI152 and UK Biobank.

Noise and artifact removal

Please see full details in K. Miller et al. Nature Neuroscience 2016.

Volume censoring

N/A

Statistical modeling & inference

Model type and settings	Linear regression, Correlation, non-parametric two-factor, two-level permutation test. See Methods for full details.
Effect(s) tested	No task or stimulation conditions involved in this study.

Specify type of analysis: ☐ Whole brain ☐ ROI-based ☒ Both

Anatomical location(s)	ROI ("imaging-derived phenotypes", IDPs) cpver the entire brain, and the entire cortical surface for Freesurfer generated ROIs.
Statistic type for inference (See Eklund et al. 2016)	N/A
Correction	The false discovery rate (FDR) was controlled at 5% using the Benjamini-Hockberg procedure.

Models & analysis

n/a	Involved in the study
☐	☐ Functional and/or effective connectivity
☐	☐ Graph analysis
☐	☐ Multivariate modeling or predictive analysis

Functional and/or effective connectivity	N/A
Graph analysis	N/A
Multivariate modeling and predictive analysis	N/A
